# Precipitants and clinical features of serotonin syndrome: a systematic review with patient-level analysis of published case reports and series

**DOI:** 10.1007/s00228-026-04118-3

**Published:** 2026-07-17

**Authors:** Bohdan Blyzniuk, Maksym Danukalo, Chiara Gastaldon, Corrado Barbui, Sermin Toto, Emanuel Raschi, Erich Seifritz, Nazar Kuzo, Georgios Schoretsanitis

**Affiliations:** 1 Clinic for Psychiatry and Psychotherapy, Psychiatric Services Aargau, Windisch, Switzerland; 2 Department of Pathological Physiology With Course in Normal Physiology, Zaporizhzhia State Medical and Pharmaceutical University, Zaporizhzhia, Ukraine; 3 Institute of Social and Preventive Medicine, Faculty of Medicine, University of Bern, Bern, Switzerland; 4 WHO Collaborating Center for Research and Training in Mental Health and Service Evaluation, Department of Neurosciences, Biomedicine and Movement Sciences, Section of Psychiatry, University of Verona, Verona, Italy; 5 Department of Psychiatry, Social Psychiatry and Psychotherapy, Hannover Medical School, Hanover, Germany; 6 Department of Medical and Surgical Sciences, University of Bologna, Bologna, Italy; 7 Department of Adult Psychiatry and Psychotherapy, University Hospital of Psychiatry Zurich, Lenggstrasse 31, 8008 Zurich, Switzerland; 8 Unit of Pharmacogenetics and Clinical Psychopharmacology, Center for Psychiatric Neurosciences, Lausanne University Hospital, Lausanne, Switzerland; 9 The Zucker Hillside Hospital, Department of Psychiatry Research, Northwell Health, Glen Oaks, NY USA; 10 Department of Psychiatry, Zucker School of Medicine at Northwell/Hofstra, Hempstead, NY USA

**Keywords:** Serotonin syndrome, Overdose, Polypharmacy, Antidepressants, Opioids

## Abstract

**Background:**

Serotonin syndrome (SS) is a concern for prescribers of serotonergic acting agents and mainly antidepressants, yet the implicated drug combinations and associated outcomes across clinical settings remain incompletely characterized.

**Methods:**

We performed a systematic review in PubMed/Embase, searching for SS cases in adults from the database’s inception until June 2024, and conducted a patient-level and network analysis of drug co-occurrence. The quality of SS diagnoses was assessed using the Hunter Serotonin Toxicity and the Sternbach Criteria.

**Results:**

A total of 764 cases were included; 653 (85.6%) and 496 (65.0%) met the Sternbach and the Hunter Criteria, respectively. Patients with SS following suicide attempts were more frequently admitted to intensive care units with higher mortality rates than patients with SS related to regular prescriptions (79.4% vs 35.6% and 18.0% vs 5.1%, respectively, both *p* < 0.001). Of 645 regular prescription cases, 92.9% were drug combinations (≥ 2 agents). Monotherapy cases were milder and often occurred after initiation of a new antidepressant in younger patients. Drug combinations involved non-antidepressants in 90.7%. Network analysis identified trazodone as the most connected antidepressant, and fentanyl and tramadol as the most connected non-antidepressant nodes. We identified five SS cases following antipsychotic discontinuation while maintaining serotonergic agents.

**Conclusions:**

Non-suicidal cases of SS during regular prescription were less serious than SS cases associated with intentional overdose. The emerging role of non-antidepressant agents (e.g., several opioids and antiparkinsonian drugs) as potential precipitants support tailored interprofessional medication review in poly-medicated subjects.

**Supplementary Information:**

The online version contains supplementary material available at 10.1007/s00228-026-04118-3.

## Introduction

Serotonin syndrome (SS) is a potentially life-threatening adverse drug reaction resulting from excessive serotonergic activity in the central nervous system [1]. Although most frequently diagnosed by exclusion, some typical symptoms may include altered mental status, autonomic dysfunction, and neuromuscular abnormalities, occurring in the context of serotonergic drug exposure [2]. Although the incidence is rather low, ranging from 0.07% to 0.23% among patients exposed to serotonergic agents, it is because of its severity that SS represents a major clinical challenge in the prescription of antidepressants [3–5]. Specifically, the mortality rate varies considerably, reaching 18% in overdose cases, while approximately 40% of all cases require intensive care unit admission (ICU) [6].The burden of SS deserves more attention when considering epidemiological data suggesting increasing polypharmacy, as the SS risk may exponentially increase with the number of serotonergic agents; for instance, there are estimates of incidence rate ratio around 5.5 in patients being co-prescribed five or more serotonergic medications compared to those on a single agent [3]. Attention regarding culprit drugs of SS may have centered around serotonergic antidepressants, although non-antidepressant medications, including opioids, antibiotics, and over-the-counter compounds, may also be implicated, especially in the context of drug-drug interactions [7–9].

Regarding the diagnosis of SS, several diagnostic criteria have been proposed [1, 2, 10], with Sternbach and Hunter Serotonin Toxicity Criteria being the most widely clinically applied [11]: Sternbach criteria, which include at least three of 10 clinical features (mental status changes, agitation, myoclonus, hyperreflexia, diaphoresis, shivering, tremor, diarrhea, incoordination, or fever) in the context of serotonergic drug combination or dose increase, with exclusion of other causes [1], and the Hunter Serotonin Toxicity Criteria, including the presence of at least one of the following: spontaneous clonus; inducible or ocular clonus with agitation or diaphoresis; tremor and hyperreflexia; or hypertonia with temperature above 38 °C and ocular or inducible clonus [2]. Although both criteria show considerable sensitivity and specificity, evidence suggests considerable shortcomings for both systems, with only 48.8% of cases meeting all Sternbach criteria, whereas applying the Hunter criteria may lead to missing of 35.7% of cases with rare presentations, such as rhabdomyolysis [11]. The diagnosis may be challenging due to the clinical presentation overlapping with other complex medication-induced adverse reactions, such as neuroleptic malignant syndrome, malignant hyperthermia, and anticholinergic toxicity [5].

Further, a ICU study reported that 7.8% of patients met Hunter criteria, suggesting that it remains a highly underdiagnosed syndrome [12]. The issue of the SS underdiagnosis may derive from the limited awareness among clinicians; data from general practice in the late 1990 s suggested that up to 85% of general practitioners were unfamiliar with the SS diagnostic criteria [13]. Moreover, a 2020 survey of neurophysicians in India found that only one third correctly recognized SS diagnostic criteria, whereas only 17% identified clonus as its most specific feature [14]. The diagnostic challenge is further complicated in multi-drug overdose settings, where prescription of antipsychotics and antidepressants may obscure the distinction between SS and neuroleptic malignant syndrome [11], and concurrent opioid-antidepressant overdose may complicate recognition of serotonergic toxicity against the backdrop of opioid-induced CNS and respiratory depression [15]. Therefore, despite decades of literature and increasing prevalence of serotonergic prescriptions, physician awareness of SS remains poor.

The SS pathophysiology involves complex interactions at multiple receptor subtypes: while the 5-HT2A receptor seems to be the primary mediator of life-threatening manifestations, including rigidity and hyperthermia, the 5-HT1A receptors may contribute to milder SS symptoms [5]. Possible pharmacodynamic mechanisms for the precipitation of SS include: a) inhibition of serotonin reuptake, b) decreased serotonin metabolism through monoamine oxidase inhibition, c) increased serotonin synthesis, d) enhanced serotonin release, and e) direct serotonin receptor agonism [7]. Apart from pharmacodynamics, pharmacokinetic interactions, particularly CYP450-mediated inhibition leading to elevated plasma concentrations of serotonergic agents [7, 16] or genetic polymorphisms in drug-metabolizing enzymes [17] resulting in higher levels of serotoninergic drugs may facilitate the SS precipitation.

Current understanding of SS remains limited by several critical knowledge gaps. SS exists on a clinical spectrum, ranging from mild neuromuscular findings to life-threatening toxicity with severe hyperthermia and rigidity [7], with clinical features and sequelae differing meaningfully across this range. Population-based and claims-based studies document escalating use of psychotropic medications and high symptom burden with polypharmacy in youth and adults, underscoring the importance of recognizing combinations that may increase SS risk [18–20]. Existing diagnostic criteria were derived primarily from overdose cases and may not accurately identify cases where standard doses are prescribed [2]. However, overdose cases usually implicate different drug exposure patterns and, thus, potentially different pharmacokinetic as well as pharmacodynamic mechanisms underlying SS, than cases with regular antidepressant doses. Therefore, comparatively studying clinical outcomes for SS in patients with regular antidepressant doses vs, overdoses is crucial. Besides, identifying co-prescription patterns in cases of SS related to either therapeutic regimens with regular antidepressant doses or overdose is of main clinical value. Our systematic analysis of published SS cases with patient-level data evaluation aimed to provide a descriptive analysis of demographic characteristics and clinical outcomes for published SS cases, including a subgroup analysis of overdoses vs. regular antidepressants prescriptions and a sensitivity analysis with monotherapy vs. drug combination cases. Last, we also aimed to identify drug-drug combinations associated with SS.

## Materials and methods

The systematic review was conducted according to a protocol prospectively registered and published on the Open Science Framework prior to the initiation of data collection and analysis [21] and reported using the Preferred Reporting Items for Systematic Reviews and Meta-Analyses (PRISMA) guidelines [22] (Supplementary table S1), modified for systematic review of case reports [23, 24].

### Search strategy

We searched MEDLINE and EMBASE to identify case reports and case series describing serotonin syndrome published in English. The search strategy included terms related to "serotonin syndrome," "serotonin toxicity," and “serotonergic syndrome” combined with publication type filters for case reports and series (Supplementary material S7). Databases were searched last in 06/2024, for publications without restrictions since database inception. Reference lists of included articles were manually searched to identify additional relevant publications not captured by the database searches.

### Study selection

After removing duplicates from both databases, two authors (BB and NK) independently screened titles and abstracts using a non-AI version of Rayyan, a web-based platform allowing independent parallel paper screening by multiple reviewers with blinded comparison of decisions and automated detection of conflicts [25]. As consensus was reached in all cases, no additional co-authors were involved. Screening was performed by inspecting titles and abstracts. When titles and abstracts did not provide information on the inclusion and exclusion criteria, full articles were obtained to verify eligibility. Cases from the same authors reporting patients with identical demographics (sex, age) and medication profiles (drugs and doses) were also considered duplicates and excluded. When the same patient experienced multiple episodes of SS within a single report, each episode was analyzed as a separate case.

### Inclusion and exclusion criteria

*Study types:* Case reports or case series reporting SS were included. Studies published on languages other than English were excluded.

*Participants:* Adults (≥ 18 years) with reported SS regardless of type of compounds were included. Pediatric cases (< 18 years) were excluded given possible substantial age-related differences in pharmacokinetics, pharmacodynamics, and prescribing patterns, as well as the fact that both applied diagnostic criteria were validated primarily in adult populations.

*Exposure:* Pharmacological treatment.

*Comparator:* Not applicable.

*Outcome:* We summarized demographic and clinical characteristics of patients with SS.

### Data extraction

Two authors (BB and MD) independently extracted demographic and clinical data using a standardized extraction form. Data extraction files were cross-checked for inconsistencies, with verification performed by a third author (NK) on a case-by-case basis. Discrepancies were resolved through discussion until consensus was reached. Authors of original reports were contacted via email for clarification of missing or ambiguous data, with a two-week waiting period for responses. Extracted data included: age, sex, diagnoses, all medications (including doses and duration), comorbidities, clinical symptoms, hospitalization, ICU admission, and clinical outcomes. Cases were classified as overdose when the reported ingested dose of at least one substance exceeded the recommended maximum daily dose; when dose information was not reported, the precipitation context was recorded as unknown; in other cases the precipitation context was assigned based on explicit reporting in the source case report as one of the following categories: introduction of a new antidepressant, antidepressant dose increase, non-antidepressant introduction or dose increase, or other reasons. The clinical context of SS precipitation within the suicide attempt or suicide was recorded as explicitly reported by the original authors.

Drugs were classified according to Anatomical Therapeutic Chemical (ATC) codes; antidepressants were defined as substances with an ATC code within the N06A class; all other substances were classified as non-antidepressants. Substances were classified as serotonergic if they exhibited one or more of the following mechanisms: serotonin reuptake inhibition, serotonin receptor agonism, serotonin release, or monoamine oxidase inhibition [7]. Pharmacological properties, including recommended maximum daily dose, serotonergic activity, and blood–brain barrier penetration, were obtained from the Database of Approved Drugs (Drugs@FDA) of the U.S. Food and Drug Administration (FDA), European Medicines Agency (EMA) Database of Approved Drugs, and DrugBank (drugbank.com). For substances not listed in these databases, we conducted targeted literature searches in MEDLINE/PubMed for pharmacodynamic data. Given the exploratory nature of our study, we adopted following criteria: A compound was classified as blood–brain barrier permeable if at least one study reported a detectable concentration of the parent drug or an active metabolite within the brain parenchyma or cerebrospinal fluid after systemic administration under non-pathological conditions in any species; A compound was classified as serotonergic if at least one study demonstrated its direct agonistic properties on any 5-HT receptor subtype, or inhibition of serotonin transporter or monoamine oxidase (MAO). We accepted any measurable affinity or the nearest reported potency metric, such as IC_50_ or pK_i_. Pharmacological agents with mixed serotonergic properties, such as second-generation antipsychotics, were classified as non-serotonergic when their dominant serotonergic action was 5-HT2A antagonism, considering it being potentially protective against SS rather than precipitating.

### Quality of studies

The quality of studies was assessed using PHARMA checklist of items for inclusion in an anecdotal report of a suspected adverse drug reaction [26]. The quality of the SS diagnosis was assessed using Hunter Serotonin Toxicity Criteria [2] and Sternbach Criteria [1]; both criteria were retrospectively applied by two authors (BB and MD), independently of the diagnostic terminology or criteria reported in the original publications. Data that the authors did not specify and could not obtain through correspondence were reported as missing.

### Statistical analysis

#### Patient-level analysis

All extracted data were pooled into a single dataset. Normality was assessed using the Shapiro–Wilk test, and homogeneity of variance using Levene’s test. Continuous variables are presented as median with interquartile range (IQR), and categorical variables as frequencies with percentages, as well as odds ratios (OR) with 95% confidence interval (95% CI). Benjamini–Hochberg multiple comparison correction procedure was performed; adjusted *p*-values were reported. A 2-sided *p*-value of 0.05 was considered statistically significant. All analyses were performed with R [27]. Parameters included in the analysis are listed in Tables 1 and 2.

**Table 1:** Bibliographic, demographic, and clinical characteristics of serotonin syndrome cases precipitated within suicide attempt/suicide vs. regular prescription

Variable	Total sample (*n* = 764)	Suicide attempt (*n* = 103)	Regular prescription (*n* = 661)	Adjusted *p*-value
Bibliographic data:				
*Region*				0.1
Asia, n (%)	159 (20.8)	16 (20.8)	143 (21.6)	
Europe, n (%)	184 (24.1)	33 (32.0)	151 (22.8)	
North America, n (%)	379 (49.6)	46 (44.7)	333 (50.4)	
Oceania, n (%)	40 (5.2)	8 (7.8)	32 (4.8)	
South America, n (%)	2 (0.3)	0 (0.0)	2 (0.3)	
Demographic and clinical data:				
^a^ Age, median (IQR)	46.0 (32.0–62.0)	35.5 (23.0–45.3)	48.0 (35.0–64.0)	** < 0.001**
^b^ Male sex, n (%)	339 (44.5)	45 (44.1)	294 (44.6)	1.0
*Psychiatric diagnoses (disorders)*				
Organic (ICD-10: F0), n (%)	16 (2.1)	0 (0.0)	16 (2.4)	0.2
Substance use (ICD-10: F1), n (%)	79 (10.3)	16 (15.5)	63 (9.5)	0.1
SCZ spectrum (ICD-10: F2), n (%)	25 (3.3)	1 (1.0)	24 (3.6)	0.3
Mood (ICD-10: F3), n (%)	510 (66.8)	70 (68.0)	440 (66.6)	0.9
Stress-related (ICD-10: F4), n (%)	145 (19.0)	12 (11.7)	133 (20.1)	0.1
Behavioral (ICD-10: F5), n (%)	22 (2.9)	2 (1.9)	20 (3.0)	0.9
Personality (ICD-10: F6), n (%)	15 (2.0)	3 (2.9)	12 (1.8)	0.6
Mental retardation (ICD-10: F7), n (%)	7 (0.9)	0 (0.0)	7 (1.1)	0.8
Developmental (ICD-10: F8), n (%)	6 (0.9)	0 (0.0)	6 (0.9)	1.0
With onset in childhood (ICD-10: F9), n (%)	7 (0.9)	2 (1.9)	5 (0.8)	0.4
Unknown/not reported, n (%)	70 (9.2)	5 (4.9)	65 (9.8)	0.2
*Physical comorbidities*				
Endocrine (ICD-10: Ex), n (%)	106 (13.9)	2 (1.9)	104 (15.7)	** < 0.001**
Neurologic (ICD-10: Gx), n (%)	103 (13.5)	6 (5.8)	97 (14.7)	**0.05**
Cardiovascular (ICD-10: Ix), n (%)	146 (19.1)	6 (5.8)	140 (21.2)	** < 0.001**
Respiratory (ICD-10: Jx), n (%)	38 (5.0)	0 (0.0)	38 (5.7)	0.05
Gastrointestinal (ICD-10: Kx), n (%)	44 (5.8)	1 (1.0)	43 (6.5)	0.1
Urogenital (ICD-10: Nx), n (%)	49 (6.4)	2 (1.9)	47 (7.1)	0.1
*Serotonin syndrome diagnosis*				
^***c***^ Hunter’s criteria fulfilled, n (%)	496 (65.0)	65 (63.1)	431 (65.3)	0.8
^***c***^ Sternbach’s criteria fulfilled, n (%)	653 (85.6)	91 (88.3)	562 (85.2)	0.6
^***c***^ Both criteria fulfilled, n (%)	489 (64.1)	65 (63.1)	424 (64.2)	0.9
^***c***^ Neither criterion fulfilled, n (%)	103 (13.5)	12 (11.7)	91 (13.8)	0.8
*Clinical outcomes*				
^b^ Hospitalization, n (%)	676 (88.8)	102 (99.0)	574 (87.2)	** < 0.001**
^d^ Intensive care unit, n (%)	315 (41.5)	81 (79.4)	234 (35.6)	** < 0.001**
^e^ Death, n (%)	51 (6.8)	18 (18.0)	33 (5.1)	** < 0.001**
*Serotonin syndrome onset after*				
New AD introduction, n (%)	205 (26.8)	5 (4.9)	200 (30.3)	** < 0.001**
AD dose increase, n (%)	53 (6.9)	0 (0.0)	53 (8.0)	**0.002**
Non-AD introduction or dose increase, n (%)	370 (48.4)	0 (0.0)	370 (56.0)	** < 0.001**
Overdose, n (%)	115 (15.1)	102 (99.0)	13 (2.0)	** < 0.001**
Other reasons, n (%)	17 (2.2)	1 (1.0)	16 (2.4)	1.0
Unknown/not described, n (%)	31 (4.1)	0 (0.0)	31 (4.7)	**0.04**

Bold font indicates significant differences after Benjamini–Hochberg multiple comparison correction

*AD* antidepressant; *SCZ* schizophrenia; *ICD-10* 10th revision of the international classification of diseases

a Data was not reported for 6 (0.8%) patients

b Data was not reported for 3 (0.4%) patients

c Data was not reported for 1 (0.1%) patients

d Data was not reported for 5 (0.7%) patients

e Data was not reported for 11 (1.4%) patients

**Table 2:** Pharmacological characteristics of serotonin syndrome cases precipitated within suicide attempt/suicide vs. regular prescription

Variable	Total sample (*n* = 764)	Suicide attempt (*n* = 103)	Regular prescription (*n* = 661)	Adjusted *p*-value
N of drugs, median (IQR)	3 (2–4)	2 (1–4)	3 (2–4)	**0.002**
N of drugs penetrating BBB, median (IQR)	3 (2–4)	2 (1–4)	3 (2–4)	**0.01**
N of serotoninergic drugs, median (IQR)	2 (1–3)	1 (1–2)	2 (1–3)	** < 0.001**
*Serotoninergic mechanism*				
SRI, n (%)	699 (91.5)	91 (88.3)	608 (92.0)	0.3
Serotonin receptor agonists, n (%)	340 (44.5)	34 (33.0)	306 (46.3)	**0.02**
Serotonin releasers, n (%)	106 (13.9)	15 (14.6)	91 (13.8)	0.8
MAO inhibitors, n (%)	209 (27.4)	20 (19.4)	189 (28.6)	0.1
*AD, n (%)*	687 (89.9)	89 (86.4)	598 (90.5)	0.2
N of AD, median (IQR)	1 (1–2)	1 (1–2)	1 (1–2)	0.2
NSMRI, n (%)	89 (11.6)	6 (5.8)	83 (12.6)	0.1
SSRI, n (%)	428 (56.0)	48 (46.6)	380 (57.5)	0.1
SSNRI, n (%)	181 (23.7)	30 (29.1)	151 (22.8)	0.2
Non-selective MAO inhibitors, n (%)	35 (4.6)	2 (1.9)	33 (5.0)	0.3
MAO-A inhibitors, n (%)	29 (4.6)	10 (9.7)	19 (2.9)	**0.002**
Other AD, n (%)	148 (19.4)	19 (18.4)	129 (19.5)	0.8
*Non-AD, n (%)*	631 (82.6)	70 (68.0)	561 (84.9)	** < 0.001**
N of non-AD, median (IQR)	2 (1–3)	1 (0–3)	2 (1–3)	**0.005**
N of non-AD penetrating BBB, median (IQR)	1 (1–2)	1 (0–2)	1 (1–3)	0.1

Bold font indicates significant differences after Holm-Bonferroni multiple comparison correction

*AD* antidepressant; *BBB* blood brain barrier; *MAO* Monoamine oxidase; *NSMRI* Non-selective monoamine reuptake inhibitor; *SRI* serotonin reuptake inhibitor; *SSNRI* selective serotonin-noradrenalin reuptake inhibitor; *SSRI* selective serotonin reuptake inhibitor

#### Suicide attempts versus regular prescription use (referred as “regular prescription”)

As intentional overdoses typically involve higher-than-recommended doses and different drug combinations than therapeutic use, we compared patients without vs. with suicide attempts/suicides (reported as such). Group comparisons used the Mann–Whitney U test for continuous variables and Pearson’s chi-square or Fisher’s exact test for categorical variables.

#### Sensitivity analysis: monotherapy versus combinations

To focus on standard clinical practice, we performed a sensitivity analysis excluding: suicide attempts/suicides, cases with the diagnosis of SS during discontinuation of a medication, and cases with the diagnosis of SS related to non-pharmacological interventions. This sensitivity analysis was performed to reduce heterogeneity considering distinct mechanisms for SS during medication discontinuation or for SS related to non-pharmacological agents; given the small number of cases excluded in sensitivity analysis, we summarized these cases within narrative synthesis. The remaining cases were categorized as monotherapy or drug combinations and compared. Comparisons of continuous variables were performed using Mann–Whitney *U*-test. Categorical variables were compared using Pearson’s Chi-square test or Fisher’s Exact, when appropriate.

#### Network analysis of drug combinations

For patients receiving ≥ 2 medications we conducted a network analysis using Gephi version 0.10.1 [28] to identify drug-drug interaction patterns. Network analysis was employed to capture the relational structure of drug co-occurrences across all cases simultaneously, identifying central and bridging medications that conventional frequency tables or pairwise comparisons cannot detect. In the network, nodes represented medications and edges represented co-occurrence within the same patient. Edge weights reflected co-occurrence frequency. We applied a minimum threshold of five cases per medication for network inclusion. All pairwise drug combinations within each case were analyzed. We calculated three well-established centrality measures: i) the degree centrality, which refers to the number of direct connections, ii) the betweenness centrality, which refers to the role in connecting different medication groups, and iii) the eigenvector centrality, which reflects the connections to other highly connected drugs.

## Results

**Fig. 1:**
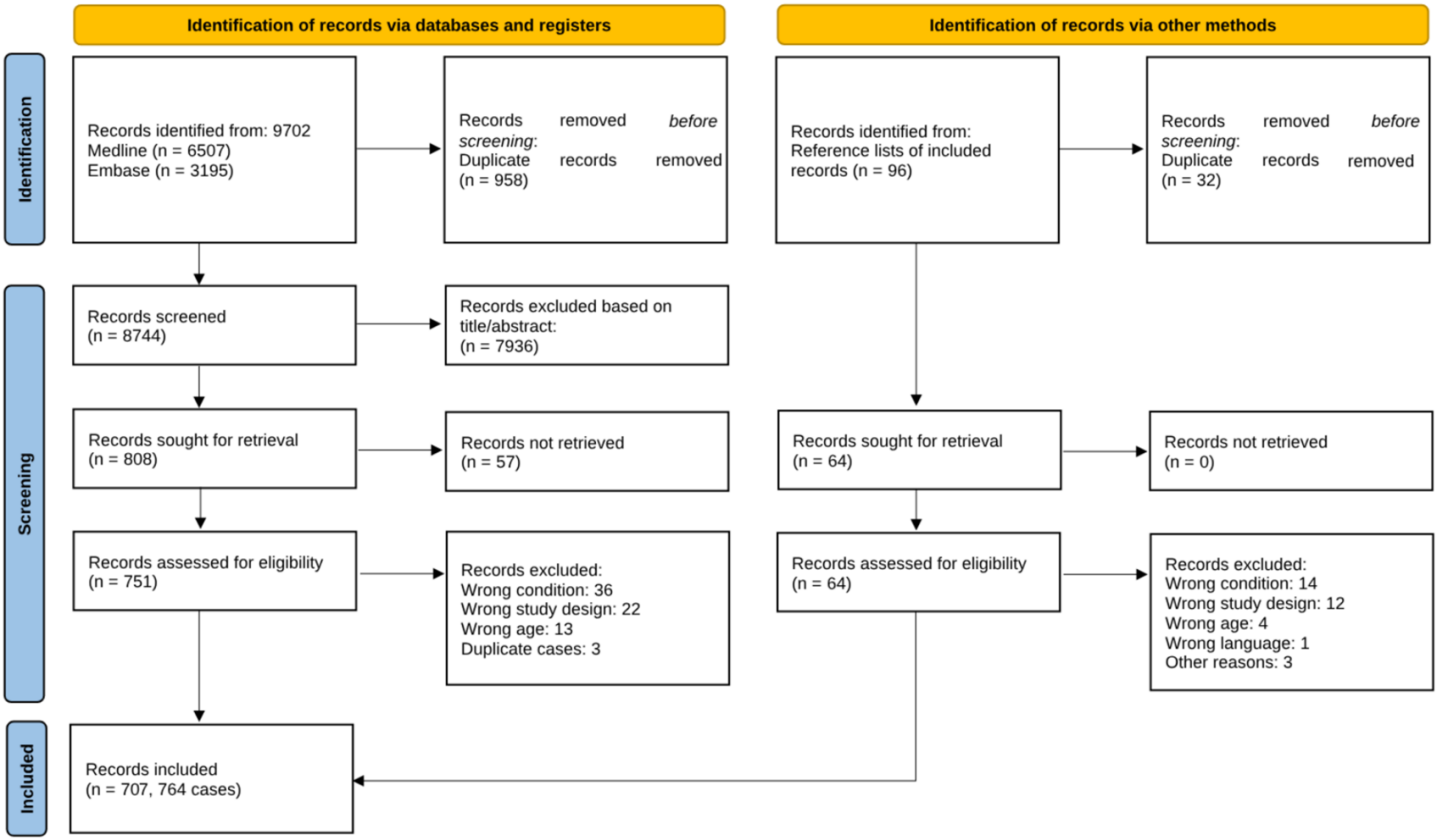
Prisma Flowchart. *From*: Page MJ, McKenzie JE, Bossuyt PM, Boutron I, Hoffmann TC, Mulrow CD, et al. The PRISMA 2020 statement: an updated guideline for reporting systematic reviews. BMJ 2021;372:n71. 10.1136/bmj.n71

The systematic review process yielded 707 publications containing 764 eligible cases (Fig. 1; Supplementary table S5). An additional search in PsycINFO and CINAHL (from inception to 5/2025) during manuscript review did not identify any further studies of interest. Most publications fulfilled the PHARMA checklist criteria (Supplementary Table S6); missing data are reported in Table 1. The median publication year was 2012 (IQR: 2004–2018). Geographic distribution showed North America as the primary source (49.6%), followed by Europe (24.1%). Descriptive data are summarized in Table 1.

### Demographic and clinical characteristics

The 764 patients had a median age of 46.0 years (IQR: 32.0–62.0), with 339 (44.5%) being male. Mood disorders were the predominant psychiatric diagnosis (*n* = 510, 66.8%), followed by stress-related disorders (*n* = 145, 19.0%) and substance use disorders (*n* = 79, 10.3%). Common medical comorbidities included cardiovascular diseases (*n* = 146, 19.1%), endocrine disorders (*n* = 106, 13.9%), and neurological conditions (*n* = 103, 13.5%). Diagnostic criteria fulfilment varied considerably: 653 cases (85.6%) met Sternbach criteria, 496 cases (65.0%) met Hunter criteria, and 489 cases (64.1%) satisfied both. Notably, 103 reported cases (13.5%) failed to meet either of the diagnostic criteria. Most patients (*n* = 676, 88.8%) required hospitalization, with 315 (41.5%) requiring ICU admission. The overall mortality rate was 6.8% (*n* = 51).

### Suicide attempts versus regular prescription

Among the 764 cases, 103 (13.5%) involved suicide attempts or completed suicides, while 661 (86.5%) occurred during regular prescription (Table 1). Suicide attempt patients were significantly younger (median 35.5 vs. 48.0 years, *p* < 0.001) and had lower rates of medical comorbidities, including endocrine (1.9% vs. 15.7%, OR = 0.10, 95% CI: 0.02–0.42, *p* < 0.001), neurologic (5.8% vs. 14.7%, OR = 0.36, 95% CI: 0.15–0.84, *p* = 0.05), and cardiovascular diseases (5.8% vs. 21.2%, OR = 0.23, 95% CI: 0.10–0.53, *p* < 0.001). The distribution of psychiatric diagnoses between suicide attempts and regular prescription patients showed no differences (*p* > 0.05). However, suicide attempts resulted in markedly worse outcomes than regular prescriptions: nearly all suicide attempt cases required hospitalization (99.0% vs. 87.2%, OR = 14.29, 95% CI: 1.97–103.65, *p* < 0.001), with the majority requiring ICU admission (79.4% vs. 35.6%, OR = 6.96, 95% CI: 4.14–11.69, *p* < 0.001). Mortality was over three times higher in suicide attempts (18.0% vs. 5.1%, OR = 4.08, 95% CI: 2.15–7.73, *p* < 0.001). Overdose precipitated SS in 99.0% of suicide attempt cases versus 2.0% of regular prescription cases (*p* < 0.001). In contrast, in regular prescription cases, SS was more frequently triggered by new antidepressant initiation (30.3% vs. 4.9%, *p* < 0.001), antidepressant dose increase (8.0% vs. 0.0%, *p* = 0.002), or non-antidepressant medication addition/dose increase, when compared to suicide attempts (56.0% vs. 0.0%, *p* < 0.001). Higher number of substances (median 3 vs. 2, *p* = 0.002), blood–brain barrier penetrating substances (median 3 vs. 2, *p* = 0.01), serotonergic drugs (median 2 vs. 1, *p* < 0.001), and non-antidepressants (median 2 vs. 1, *p* = 0.005) were involved in regular prescription cases compared to suicide attempt cases. Among antidepressants, only MAO-A inhibitors were more frequently involved in suicide attempts (9.7% vs. 2.9%, OR = 3.62, 95% CI: 1.60–8.21, *p* = 0.002), while there were no differences for other antidepressant classes, when compared to the cases with regular prescription (Table 2).

### Sensitivity analysis: monotherapy versus drug combinations

After excluding suicide attempts (*n* = 103), drug discontinuation cases (*n* = 5), and non-pharmacological precipitants (*n* = 12), 645 cases remained for analysis. Of these, 46 (7.1%) involved monotherapy and 599 (92.9%) involved drug combinations (Supplementary Table S2). Monotherapy patients were younger (median 34.0 vs. 49.0 years, *p* = 0.02) with lower rates of cardiovascular comorbidities (4.3% vs. 22.9%, OR = 0.15, 95% CI: 0.04–0.61, *p* = 0.01). No significant differences emerged between psychiatric diagnoses or SS diagnostic criteria fulfilment. However, monotherapy cases demonstrated milder clinical courses, with lower hospitalization rates (73.9% vs. 87.9%, OR = 0.39, 95% CI: 0.19–0.82, *p* = 0.02) and a trend toward fewer ICU admissions (21.7% vs. 36.6%, OR = 0.48, 95% CI: 0.23–1.00, *p* = 0.09). Mortality rates were similar between groups (4.3% vs. 5.2%, *p* = 1.0). The initiation of new antidepressant(s) was reported in 65.2% of monotherapy cases compared to 28.4% of combination cases (OR = 4.71, 95% CI: 2.52–8.79, *p* < 0.001). Conversely, a dose increase of a non-antidepressant medication precipitated 61.1% of drug combination cases versus 8.7% of monotherapy cases (OR = 0.06, 95% CI: 0.02–0.17, *p* < 0.001). Monotherapy cases less frequently involved serotonin receptor agonists (13.0% vs. 48.4%, OR = 0.16, 95% CI: 0.07–0.39, *p* < 0.001), serotonin releasing agents (2.2% vs. 15.0%, OR = 0.13, 95% CI: 0.02–0.93, *p* < 0.001), and MAO inhibitors (6.5% vs. 30.9%, OR = 0.16, 95% CI: 0.05–0.52, *p* < 0.001). Strikingly, 90.7% of drug combination cases involved at least one non-antidepressant medication versus only 4.3% of monotherapy cases (OR = 0.004, 95% CI: 0.001–0.02, *p* < 0.001; Supplementary Table S3).

### Network analysis of drug combinations

**Fig. 2:**
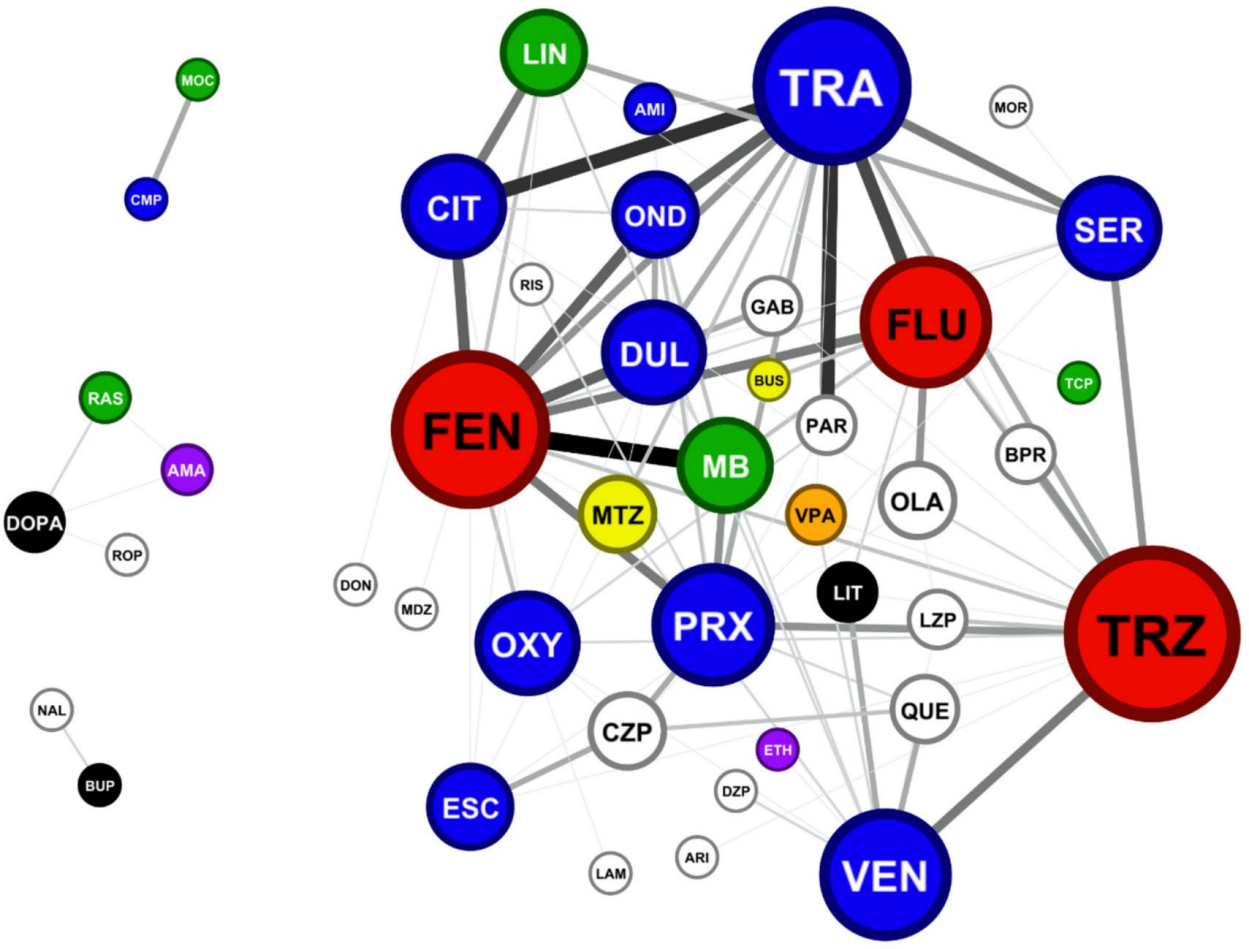
Network of co-occurring drug combinations in serotonin syndrome cases. A minimum inclusion threshold of ≥ 5 co-occurrences was applied. Nodes represent drugs; edges indicate co-occurrence within the same case. Edge thickness scales with co-occurrence frequency. Node size scales with degree centrality. Node color denotes drug mechanism: blue: SRI; red: SRI and SRA; yellow: SRA; black: SR, orange: SRA and MAOi; violet: SRA and SR; green: MAOi; white: not serotonergic. *AMA* amantadine; *AMI* amitriptyline; *ARI* aripiprazole; *BPR* bupropion; *BUP* buprenorphine; *BUS* buspirone; *CIT* citalopram; *CMP* clomipramine; *CZP* clonazepam; *DON* donepezil; *DOPA* levodopa; *DUL* duloxetine; *DZP* diazepam; *ESC* escitalopram; *ETH* ethanol; *FEN* fentanyl; *FLU* fluoxetine; *GAB* gabapentin; *LAM* lamotrigine; *LIN* linezolid; *LIT* lithium; *LZP* lorazepam; *MAOi* monoamine oxidase inhibitor; *MB* methylene blue; *MDZ* midazolam; *MOC* moclobemide; *MOR* morphine; *MTZ* mirtazapine; *NAL* naloxone; *OLA* olanzapine; *OND* ondansetron; *OXY* oxycodone; *PAR* paracetamol; *PRX* paroxetine; *QUE* quetiapine; *RAS* rasagiline; *RIS* risperidone; *ROP* ropinirole; *SER* sertraline; *SR* serotonin releaser; *SRA* serotonin receptor agonist; *SRI* serotonin reuptake inhibitor; *TCP* tranylcypromine; *TRA* tramadol; *TRZ* trazodone; *VEN* venlafaxine; *VPA* valproate

We included 599 SS cases with drug combinations in our network analysis, which revealed four clusters of drug co-occurrence patterns (Fig. 2, Supplementary Table S4). The main cluster encompassed most medications and represented the core drug interaction network. The degree centrality analysis identified trazodone (16 connections), fentanyl (14 connections), and tramadol (14 connections) as the most frequently co-prescribed medications. SSRIs and SNRIs showed intermediate connectivity: fluoxetine and venlafaxine (11 connections each), paroxetine (10 connections), citalopram, duloxetine, and sertraline (8 connections each; Supp. Table 2). Betweenness centrality, indicating a drug’s role in bridging different medication groups, revealed trazodone as the primary connector, followed by venlafaxine, fluoxetine, and tramadol. Despite having fewer connections than trazodone, fentanyl demonstrated the highest eigenvector centrality, indicating its connections to other highly connected drugs.

Cluster 2 comprised antiparkinsonian medications (amantadine, levodopa, rasagiline, ropinirole), forming an isolated subnetwork with minimal connections to the main cluster. Cluster 3 contained only clomipramine and moclobemide, while Cluster 4 included buprenorphine and naloxone (Supplementary Table S2).

### Cases excluded from the sensitivity analysis

Five patients developed SS following antipsychotic discontinuation (clozapine *n* = 3, olanzapine *n* = 2) while continuing serotonergic medications [29–33]. Patients’ age ranged from 22–62 years, with symptom onset occurring from < 24 h to 15 days post-discontinuation. Two met Hunter criteria, all met Sternbach criteria, required hospitalization, and recovered completely after discontinuing serotonergic agent(s) with supportive care and treatment with cyproheptadine (*n* = 3). In four patients, antipsychotic treatment was restarted.

Seven patients (six females, age range 31–70 years) developed SS during electroconvulsive therapy (ECT) while on stable serotonergic medications. Symptom onset varied from immediately after the first ECT to the thirteenth ECT session. Five patients met Sternbach criteria, and three met Hunter criteria. Additional features included rigidity in four cases, while one patient presented with hypersalivation and verbigeration consistent with catatonic features. Further unique cases were precipitated by repetitive transcranial magnetic stimulation (TMS; *n* = 1), spinal cord stimulation (SCS; *n* = 2), hemodialysis (*n* = 1), and extreme heat exposure (*n* = 1). All patients were on stable serotonergic regimens prior to the precipitating event and recovered with the discontinuation of serotonergic medications and supportive care.

## Discussion

Our systematic review of 764 SS cases reveals distinct patterns challenging traditional considerations. Suicide attempt cases with intentional overdoses led to markedly severe outcomes with 79.4% requiring ICU admission and 18.0% mortality, compared to only 35.6% ICU admission and 5.1% mortality in cases with regular prescription. This practically implies that SS may be less serious when occurring in patients receiving regular doses of antidepressants. These findings suggest that SS occurring in the context of therapeutic dosing may generally follow a less severe course than SS associated with intentional overdose, though individual patient factors, including suicide risk, should always be considered. Importantly, the 94.9% survival rate in regular prescription cases suggests that SS, when occurring at therapeutic doses, may follow a benign course with appropriate supportive care [2]. Interestingly, MAO-A inhibitors were disproportionately represented in overdose cases (9.7% vs. 2.9%), which may partly reflect their known potential for severe toxicity due to irreversible enzyme inhibition and prolonged duration of action [34], though prescribing pattern differences between groups cannot be entirely excluded. Further, the younger age of overdose patients and their lower burden of medical comorbidities also suggest that the outcome of SS may depend on the serotonergic excess and is less affected by patient vulnerability factors.

In patients developing SS within regular prescription, our analysis revealed distinct patterns between monotherapy- and drug combination-induced syndromes; monotherapy cases represented a small proportion, 7.1% of regular prescription cases; these cases occurred predominantly in younger patients following antidepressant initiation, suggesting an idiosyncratic reaction. The milder clinical course with lower hospitalization rates and ICU admissions observed in monotherapy cases supports the hypothesis that single-mechanism serotonergic excess may produce less severe toxicity than multi-mechanism synergistic effects [35]. By contrast, cases involving drug combinations were usually the result of adding non-antidepressants to regimens that included multiple serotonergic mechanisms, such as receptor agonism (48.4%), serotonin release (15.0%), and MAO inhibition (30.9%). This mechanistic diversity in drug combination cases may explain their greater clinical severity; thus, we assume a simultaneous activation of multiple pathways, which may produce synergistic rather than additive serotonergic effects [36]. The striking finding that 90.7% of drug combination cases involved non-antidepressant medications, i.e. medications licensed for the treatment of major depression, challenges the traditional conceptualization of SS as primarily an antidepressant-related phenomenon and highlights the need for an interprofessional approach, including team-based medication review [37].

Our network analysis provides the first systematic mapping of drug-drug interaction patterns in SS, revealing central roles for medications beyond traditional antidepressants. While SSRIs and SNRIs were predictably common, trazodone emerged as the most highly connected node among interactions. This likely reflects its widespread off-label use for insomnia in patients already receiving other antidepressants, with prescriptions as a sleep aid exceeding those for depression [38, 39]. This finding has clinical implications, as trazodone’s complex pharmacology, with its 5-HT2A antagonism at low doses, but essential serotonin reuptake inhibition at higher doses, as well as the active metabolite m-chlorophenylpiperazine, which acts as a non-selective 5-HT receptor agonist, may have been underappreciated as a SS risk factor, when combined with other serotonergic substances [39, 40].

The central role of several opioids in our network, with fentanyl showing the highest eigenvector centrality despite fewer direct connections, highlights that patients receiving opioids may be particularly exposed to the SS risk. This observation is worrisome given the increasing co-prescription of opioids with antidepressants for chronic pain; in fact, epidemiological studies suggest that up to 60% of chronic opioid users receive concurrent antidepressant therapy [41]. The serotonergic properties of opioids vary considerably; oxycodone may inhibit the serotonin reuptake [42], whereas tramadol is a serotonin and noradrenaline reuptake inhibitor [43, 44]. In vitro data suggest that fentanyl may inhibit serotonin reuptake at clinically relevant concentrations and may also indirectly increase serotonin release through complex receptor interactions [45, 46]. The high centrality of fentanyl in our network may be due to use in perioperative and critical care settings. Additionally, the opioid crisis, with fentanyl now involved in over 70% of opioid overdose deaths in the United States [47], points out another dimension of SS risk: the concurrent use of fentanyl with recreational drugs possessing serotonergic properties, such as MDMA, amphetamines, and cocaine, has been increasingly documented, with polysubstance abuse present in approximately half of fentanyl-related fatalities [48]. In our dataset, such co-occurrences were observed below the predefined threshold of five cases, likely because fentanyl overdoses often result in fatal respiratory depression before SS can develop [49]. Nevertheless, given the high prevalence of antidepressant prescriptions in individuals with substance use [50, 51], the SS risk may be elevated in both emergency and addiction medicine settings. Our finding that some opioids were reported as part of interactions between diverse medication classes suggests they may act as pharmacological amplifiers, increasing the risk of SS even in combinations generally considered safe.

However, in contrast to tramadol and fentanyl, buprenorphine appeared in an isolated cluster alongside its combination partner naloxone. This pattern is consistent with buprenorphine’s limited serotonergic profile: unlike other common opioids, which demonstrate clearer serotonergic mechanisms or stronger clinical associations with SS, buprenorphine is generally considered to carry lower serotonergic toxicity risk [52, 53]. Rare cases involving buprenorphine with serotonergic co-medications indicate that this risk is not absent, but its network isolation supports it being a less prominent driver of SS compared to other opioids.

Besides, the network structure revealed that medications with MAOI properties, including linezolid and methylene blue, showed high interconnectivity, consistent with their well-documented potential to precipitate severe SS when combined with any serotonergic agent [54, 55].

Of note, antiparkinsonian medications may be involved in SS in a distinct network cluster, disconnected from antidepressant nodes. This pattern highlights a previously underrecognized risk group. While antiparkinsonian drugs primarily target dopaminergic pathways, they possess inherent serotonergic properties: rasagiline and selegiline can inhibit MAO-A at higher doses or in poor metabolizers [56], and amantadine inhibits serotonin reuptake and enhances release [57] Levodopa may increase acute serotonin release [58], and serotonergic neurons may decarboxylate levodopa to dopamine [59], while experimental data also report levodopa-associated chronic serotonergic depletion [60], suggesting it may contribute to SS through complex perturbation of serotonergic-dopaminergic networks. These properties become clinically relevant given that depression affects 35–40% of Parkinson’s disease patients, often requiring antidepressant therapy [61]. Furthermore, the SS diagnosis in this patient population may be complicated by symptom overlap: parkinsonian tremor, rigidity, and autonomic dysfunction may mimic SS features, with only inducible clonus being a reliable distinguisher [12]. These findings emphasize that several antiparkinsonian medications, such as levodopa, rasagiline and ropinirole, should be considered potentially serotonergic, particularly when initiating antidepressant therapy in this population.

Last, the identification of SS following antipsychotic discontinuation represents a potentially novel mechanism that expands the traditional paradigm [7]. We identified five SS cases after stopping olanzapine or clozapine while continuing stable serotonergic medications. This phenomenon likely reflects the potent 5-HT2A receptor antagonism of these antipsychotics, which may have “protected” from the serotonergic toxicity during co-administration with antidepressants [62]. The pathophysiology seems to involve receptor adaptation, as chronic 5-HT2A blockade may lead to receptor upregulation and hypersensitivity, such that removal of the antagonist unmasks latent serotonergic excess from ongoing antidepressant therapy [63, 64]. The clinical implications are substantial, as the combination of antipsychotics with antidepressants occurs in up to 50% of patients with treatment-resistant depression or bipolar disorder [65]. This highlights the need for monitoring when discontinuing antipsychotics with strong 5-HT2A antagonism in patients prescribed serotonergic medications, with consideration for gradual tapering over abrupt discontinuation.

We also observed that non-pharmacologic interventions such as ECT, TMS and SCS may be associated with SS if the patient’s serotonergic system is primed by medication. While the mechanisms remain unclear, all three interventions are known to acutely alter neurotransmitter dynamics and may increase serotonergic activity acting in synergy [66–68].

The 13.5% of cases fulfilling neither the Hunter nor the Sternbach criteria likely reflect multiple factors such as known shortcomings of both diagnostic systems [69]; for instance, the Sternbach criteria are more prone to false positives through its broader thresholds [2], whereas the Hunter criteria may miss atypical presentations [70]. Moreover, incomplete clinical documentation may have also played a role, given that unreported symptoms were treated as absent. A minority of these cases may also represent genuine misdiagnoses, further underscoring that SS remains a clinical diagnosis of exclusion highly dependent on documentation quality and clinician awareness [7, 69].

We acknowledge several limitations inherent to analyzing case reports. Publication bias may skew data toward severe cases, potentially overestimating SS severity and mortality; on the other hand, mild SS cases that resolve without intervention or remain unrecognized may be underrepresented in our analysis. The retrospective, descriptive design precluded determination of incidence rates or prospective risk assessment. Incomplete reporting was common, with missing data on doses, symptom onset timing, and the application of diagnostic criteria. Furthermore, the comparison between suicide attempt and regular prescription cases may be subject to residual confounding by unmeasured baseline differences, including psychiatric symptom severity, which cannot be systematically captured by case report data. We assumed unreported symptoms as being absent, which may not precisely reflect clinical reality. Diagnostic criteria varied across the decades spanned by our review, potentially affecting case inclusion. Our network analysis cannot establish causality. High centrality of drugs may reflect prescription frequency rather than inherent SS risk. Further, collinearity presents another challenge, as drugs may co-occur due to common prescribing patterns rather than specific pharmacodynamic/pharmacokinetic interactions, which could not be systematically assessed due to insufficient data in the source case reports. Particularly, for pharmacokinetic interactions, the lack of data regarding medication concentrations as well as cytochrome P450 metabolism and pharmacogenetic variability was a main limitation. Lastly, as this topic continues to attract clinical research interest, future reviews will need to update patient-level analyses, given that approximately 20 new SS-related papers are published annually.

Thus, our network findings should be considered as hypothesis-generating and not as proof of drug-drug interactions. Finally, restriction to English-language reports and exclusion of pediatric cases limits generalizability. Despite these caveats, the large number of cases provides robust descriptive data.

In conclusion, our analysis suggests that SS arising during the standard prescription may be substantially less worrisome than in the context of the suicidal overdose. Prescription of at least two medications was dominant among SS reports and led to more severe clinical courses with higher hospitalization rates compared to monotherapy-related SS. The predominance of non-antidepressant medications, particularly several opioids, challenges traditional conceptualizations of SS as primarily an antidepressant phenomenon. Additionally, the involvement of non-antidepressants suggests the need for interprofessional medication review. A novel precipitation mechanism, including the discontinuation of antipsychotics, expands the clinical scenarios related to SS. Despite limitations inherent to case report analysis, our comprehensive characterization provides an evidence-based framework for preventing this potentially fatal but largely avoidable adverse drug reaction.

## Supplementary Information

Below is the link to the electronic supplementary material.

Supplementary file1 (PDF 3055 KB)

## Data Availability

There is no original data reported in this manuscript.

## Declarations

### Generative artificial intelligence

During the preparation of this manuscript, authors used Microsoft Copilot integrated into Microsoft Word to assist with language editing such as improving clarity, style, and grammar. No generative AI tool was used to generate scientific content. Authors reviewed the text and take full responsibility for the content of the manuscript.
