# Supplementary material for: Precipitants and clinical features of serotonin syndrome: a systematic review with patient-level analysis of published case reports and series
Source: Eur J Clin Pharmacol. 2026 Jul 17;82(8):210. doi: 10.1007/s00228-026-04118-3 (PMC13375959; doi:10.1007/s00228-026-04118-3)
Supplement: Supplementary file 1 — Supplementary file1 (PDF 3055 KB) [file 228_2026_4118_MOESM1_ESM.pdf]

Supplementarty Table S1. PRISMA checklist

Section and Topic	Item #	Checklist item	Location where item is reported
**TITLE**			
Title	1	Identify the report as a systematic review.	Title
**ABSTRACT**			
Abstract	2	See the PRISMA 2020 for Abstracts checklist.	Abstract
**INTRODUCTION**			
Rationale	3	Describe the rationale for the review in the context of existing knowledge.	Page 7
Objectives	4	Provide an explicit statement of the objective(s) or question(s) the review addresses.	Page 9
**METHODS**			
Eligibility criteria	5	Specify the inclusion and exclusion criteria for the review and how studies were grouped for the syntheses.	Page 10
Information sources	6	Specify all databases, registers, websites, organisations, reference lists and other sources searched or consulted to identify studies. Specify the date when each source was last searched or consulted.	Page 9
Search strategy	7	Present the full search strategies for all databases, registers and websites, including any filters and limits used.	Page 9
Selection process	8	Specify the methods used to decide whether a study met the inclusion criteria of the review, including how many reviewers screened each record and each report retrieved, whether they worked independently, and if applicable, details of automation tools used in the process.	Page 10
Data collection process	9	Specify the methods used to collect data from reports, including how many reviewers collected data from each report, whether they worked independently, any processes for obtaining or confirming data from study investigators, and if applicable, details of automation tools used in the process.	Page 10
Data items	10a	List and define all outcomes for which data were sought. Specify whether all results that were compatible with each outcome domain in each study were sought (e.g. for all measures, time points, analyses), and if not, the methods used to decide which results to collect.	Page 10
	10b	List and define all other variables for which data were sought (e.g. participant and intervention characteristics, funding sources). Describe any assumptions made about any missing or unclear information.	Page 11
Study risk of bias assessment	11	Specify the methods used to assess risk of bias in the included studies, including details of the tool(s) used, how many reviewers assessed each study and whether they worked independently, and if applicable, details of automation tools used in the process.	Not applicable
Effect measures	12	Specify for each outcome the effect measure(s) (e.g. risk ratio, mean difference) used in the synthesis or presentation of results.	Page 12
Synthesis methods	13a	Describe the processes used to decide which studies were eligible for each synthesis (e.g. tabulating the study intervention characteristics and comparing against the planned groups for each synthesis (item #5)).	Page 10
	13b	Describe any methods required to prepare the data for presentation or synthesis, such as handling of missing summary statistics, or data conversions.	Page 11
	13c	Describe any methods used to tabulate or visually display results of individual studies and syntheses.	Page 11
	13d	Describe any methods used to synthesize results and provide a rationale for the choice(s). If meta-analysis was performed, describe the model(s), method(s) to identify the presence and extent of statistical heterogeneity, and software package(s) used.	Page 12
	13e	Describe any methods used to explore possible causes of heterogeneity among study results (e.g. subgroup analysis, meta-regression).	Not applicable
	13f	Describe any sensitivity analyses conducted to assess robustness of the synthesized results.	Page 12
Reporting bias assessment	14	Describe any methods used to assess risk of bias due to missing results in a synthesis (arising from reporting biases).	Page 11
Certainty assessment	15	Describe any methods used to assess certainty (or confidence) in the body of evidence for an outcome.	Page 11
**RESULTS**			
Study selection	16a	Describe the results of the search and selection process, from the number of records identified in the search to the number of studies included in the review, ideally using a flow diagram.	Page 13
	16b	Cite studies that might appear to meet the inclusion criteria, but which were excluded, and explain	Not applicable

Section and Topic	Item #	Checklist item	Location where item is reported
		why they were excluded.	
Study characteristics	17	Cite each included study and present its characteristics.	Supplementary table S5
Risk of bias in studies	18	Present assessments of risk of bias for each included study.	Supplementary table S6
Results of individual studies	19	For all outcomes, present, for each study: (a) summary statistics for each group (where appropriate) and (b) an effect estimate and its precision (e.g. confidence/credible interval), ideally using structured tables or plots.	Tables 1, 2
Results of syntheses	20a	For each synthesis, briefly summarise the characteristics and risk of bias among contributing studies.	Not applicable
	20b	Present results of all statistical syntheses conducted. If meta-analysis was done, present for each the summary estimate and its precision (e.g. confidence/credible interval) and measures of statistical heterogeneity. If comparing groups, describe the direction of the effect.	Page 14, Table 1,2
	20c	Present results of all investigations of possible causes of heterogeneity among study results.	Not applicable
	20d	Present results of all sensitivity analyses conducted to assess the robustness of the synthesized results.	Page 16, Table 3, 4, Figure 2
Reporting biases	21	Present assessments of risk of bias due to missing results (arising from reporting biases) for each synthesis assessed.	Not applicable
Certainty of evidence	22	Present assessments of certainty (or confidence) in the body of evidence for each outcome assessed.	Table 1
**DISCUSSION**			
Discussion	23a	Provide a general interpretation of the results in the context of other evidence.	Page 20
	23b	Discuss any limitations of the evidence included in the review.	Page 24
	23c	Discuss any limitations of the review processes used.	Page 24
	23d	Discuss implications of the results for practice, policy, and future research.	Page 25
**OTHER INFORMATION**			
Registration and protocol	24a	Provide registration information for the review, including register name and registration number, or state that the review was not registered.	Page 9
	24b	Indicate where the review protocol can be accessed, or state that a protocol was not prepared.	Page 9
	24c	Describe and explain any amendments to information provided at registration or in the protocol.	Not applicable
Support	25	Describe sources of financial or non-financial support for the review, and the role of the funders or sponsors in the review.	Page 3
Competing interests	26	Declare any competing interests of review authors.	Page 3
Availability of data, code and other materials	27	Report which of the following are publicly available and where they can be found: template data collection forms; data extracted from included studies; data used for all analyses; analytic code; any other materials used in the review.	Not provided

From: Page MJ, McKenzie JE, Bossuyt PM, Boutron I, Hoffmann TC, Mulrow CD, et al. The PRISMA 2020 statement: an updated guideline for reporting systematic reviews. BMJ 2021;372:n71. doi: 10.1136/bmj.n71

Supplementary Table S2. Demographic and clinical characteristics of serotonin syndrome cases precipitated with monotherapy vs. drug combinations (no suicide, no other reasons)

Variable	Monotherapy (n = 46)	Drug combinations (n = 599)	Adjusted p-value
**Demographic and clinical data:**			
[a] Age, median (IQR)	34.0 (25.0 – 54.5)	49.0 (36.0 – 64.0)	**0.02**
[b] Male sex, n (%)	21 (45.7)	266 (44.6)	1.0
Psychiatric diagnoses (disorders)			
Organic (ICD-10: F0), n (%)	1 (2.2)	14 (2.3)	1.0
Substance use (ICD-10: F1), n (%)	5 (10.9)	57 (9.5)	1.0
SCZ spectrum (ICD-10: F2), n (%)	1 (2.2)	19 (3.2)	1.0
Mood (ICD-10: F3), n (%)	30 (65.2)	399 (66.6)	1.0
Bipolar disorder (ICD-10: F31), n (%)	1 (2.2)	54 (8.9)	0.2
Depression (ICD-10: F32, F33), n (%)	28 (60.9)	346 (56.7)	0.6
Stress-related (ICD-10: F4), n (%)	6 (13.0)	121 (20.2)	0.7
Behavioral (ICD-10: F5), n (%)	0 (0.0)	19 (3.2)	0.8
Personality (ICD-10: F6), n (%)	1 (2.2)	10 (1.7)	0.9
Mental retardation (ICD-10: F7), n (%)	0 (0.0)	7 (1.2)	1.0
Developmental (ICD-10: F8), n (%)	1 (2.2)	5 (0.8)	0.8
With onset in childhood (ICD-10: F9), n (%)	0 (0.0)	5 (0.8)	1.0
Unknown / not reported, n (%)	3 (6.5)	62 (10.4)	0.9
Nonpsychiatric diagnoses (disorders)			
Endocrine (ICD-10: Ex), n (%)	2 (4.3)	100 (16.7)	0.1
Neurologic (ICD-10: Gx), n (%)	8 (17.4)	85 (14.2)	1.0
Cardiovascular (ICD-10: Ix), n (%)	2 (4.3)	137 (22.9)	**0.01**
Respiratory (ICD-10: Jx), n (%)	2 (4.3)	35 (5.8)	1.0
Gastrointestinal (ICD-10: Kx), n (%)	0 (0.0)	41 (6.8)	0.4
Urogenital (ICD-10: Nx), n (%)	1 (2.2)	45 (7.5)	0.7
Serotonin syndrome			
[c] Hunter's criteria fulfilled, n (%)	30 (66.7)	391 (65.3)	1.0
[c] Sternbach's criteria fulfilled, n (%)	41 (91.1)	506 (84.5)	0.7
[c] Both criteria fulfilled, n (%)	30 (66.7)	384 (64.1)	1.0
[c] Neither criterion fulfilled, n (%)	4 (8.9)	86 (14.4)	0.8
Clinical outcomes			
[d] Hospitalization, n (%)	34 (73.9)	524 (87.9)	**0.02**
[e] Intensive care unit, n (%)	10 (21.7)	218 (36.6)	0.1
[f] Death, n (%)	2 (4.3)	31 (5.2)	1.0
Serotonin syndrome onset after			
New AD introduction, n (%)	30 (65.2)	170 (28.4)	**<0.001**
AD dose increase, n (%)	7 (15.2)	46 (7.7)	0.3
Non-AD introduction or dose increase, n (%)	4 (8.7)	366 (61.1)	**<0.001**
Overdose, n (%)	2 (4.3)	11 (1.8)	0.8
Unknown / not described, n (%)	3 (6.5)	28 (4.7)	0.9

Bold font indicates significant differences after Benjamini-Hochberg multiple comparison correction.
AD: antidepressant
SCZ: schizophrenia
ICD-10: 10th revision of the International Classification of Diseases

a Data was not reported for 5 (0.8%) patients
b Data was not reported for 2 (0.3%) patients
c Data was not reported for 1 (0.2%) patients
d Data was not reported for 3 (0.5%) patients
e Data was not reported for 4 (0.6%) patients
f Data was not reported for 8 (1.2%) patients

Supplementary Table S3. Pharmacological clinical characteristics of serotonin syndrome cases precipitated with monotherapy vs. drug combinations (no suicide, no other reasons)

Variable	Monotherapy (n = 46)	Drug combinations (n = 599)	Adjusted p-value
Serotoninergic mechanism			
SRI, n (%)	39 (84.8)	554 (92.5)	0.1
Serotonin receptor agonists, n (%)	6 (13.0)	290 (48.4)	**<0.001**
Serotonin releasers, n (%)	1 (2.2)	90 (15.0)	**0.04**
MAO inhibitors, n (%)	3 (6.5)	185 (30.9)	**<0.001**
AD, n (%)	44 (95.7)	539 (90.0)	0.5
NSMRI, n (%)	4 (8.7)	77 (12.9)	0.6
SSRI, n (%)	26 (56.5)	348 (58.1)	0.9
SSNRI, n (%)	8 (17.4)	137 (22.9)	0.5
Non-selective MAO inhibitors, n (%)	1 (2.2)	32 (5.3)	0.6
MAO-A inhibitors, n (%)	2 (4.3)	16 (2.7)	0.7
Other AD, n (%)	3 (6.5)	124 (20.7)	**0.05**
Non-AD, n (%)	2 (4.3)	543 (90.7)	**<0.001**

Bold font indicates significant differences after Benjamini-Hochberg multiple comparison correction.
AD: antidepressant
MAO: Monoamine oxidase
NSMRI: Non-selective monoamine reuptake inhibitor
SRI: serotonin reuptake inhibitor;
SSNRI: selective serotonin-noradrenalin reuptake inhibitor;
SSRI: selective serotonin reuptake inhibitor;

Supplementary Table S4. Network analysis of drug combinations associated with serotonin syndrome

Substance	Degree centrality	Betweenness centrality	Eigenvector centrality
**Cluster 1**			
Amitriptyline	2	0.0	0.2
Aripiprazole	1	0.0	0.1
Bupropion	3	0.0	0.3
Buspirone	1	0.0	0.1
Citalopram	8	41.9	0.6
Clonazepam	5	7.9	0.3
Diazepam	1	0.0	0.1
Donepezil	1	0.0	0.1
Duloxetine	8	11.0	0.6
Escitalopram	6	7.4	0.4
Ethanol	1	0.0	0.1
Fentanyl	14	73.3	1.0
Fluoxetine	11	81.8	0.7
Gabapentine	3	0.7	0.3
Lamotrigine	1	0.0	0.1
Linezolid	6	14.4	0.5
Lithium	3	2.3	0.3
Lorazepam	3	1.5	0.2
Methylene blue	7	20.0	0.6
Midazolam	1	0.0	0.1
Mirtazapine	5	6.2	0.4
Morphine	1	0.0	0.1
Olanzapine	5	8.4	0.4
Ondansetron	6	2.4	0.5
Oxycodone	8	54.8	0.5
Paracetamol	3	1.8	0.3
Paroxetine	10	58.6	0.6
Quetiapine	4	5.4	0.3
Risperidone	1	0.0	0.1
Sertraline	8	43.2	0.6
Tramadol	14	79.1	0.9
Tranylcypromine	1	0.0	0.1
Trazodone	16	135.1	0.9
Valproate	3	1.5	0.3
Venlafaxine	11	89.4	0.5
**Cluster 2**			
Amantadine	2	0.0	0.0
Levodopa	3	2.0	0.0
Rasagiline	2	0.0	0.0
Ropinirole	1	0.0	0.0
**Cluster 3**			
Clomipramine	1	0.0	0.0
Moclobemide	1	0.0	0.0
**Cluster 4**			
Buprenorphine	1	0.0	0.0
Naloxone	1	0.0	0.0

Supplementary Table S5. Characteristics of included case reports

Authors	Year	Journal, Volume, Issue	Country	Age	Sex	Primary psychiatric diagnosis	Medication	Hunter's criteria fulfilled	Sternbach's criteria fulfilled	Hospital admission	ICU admission	Death
Abdelrahman A. et al	2018	Am. J. Respir. Crit. Care Med., 197.	USA	54	female	depression	citalopram, nortriptyline buprenorphine, naloxone	yes	yes	yes	yes	no
Abdu et al	2019	Crit. Care Med., 47(1)	USA	23	male	NA	tianeptine	yes	yes	yes	no	no
Adan-Manes et al	2006	Journal of Clinical Pharmacy and Therapeutics, 31(4)	Spain	71	female	recurrent depressive episodes	venlafaxine, lithium	yes	yes	yes	no	no
Adler et al	2015	A & A Case Reports, 5(9)	USA	20	male	depression	fluoxetine, fentanyl, metoclopramide, hydromorphone, methylene blue	yes	yes	yes	yes	no
Adson et al	2001	Annals of Pharmacotherapy, 35(11)	USA	22	male	depression	sertraline, trazodone	no	yes	yes	yes	no
Ailawadhi et al	2007	Journal of Clinical Pharmacy and Therapeutics, 32(2)	USA	65	female	depression	citalopram, fentanyl, rabeprazole, tolterodine, hydrocodone	yes	yes	yes	no	no
Akin et al	2008	Am. J. Case Rep., 9	Turkey	31	male	minor depression	sertraline	yes	yes	yes	yes	no
Alghamdi et al	2018	Neurologia i Neurochirurgia Polska, 52(2)	Saudi Arabia	52	male	depression	citalopram	NA	NA	yes	no	yes
Alibegović et al	2019	Forensic Science, Medicine and Pathology, 15(2)	Slovenia	30	female	depression	venlafaxine, duloxetine	no	yes	yes	yes	yes
Alkhatib et al	2010	Dig. Dis. Sci., 55(1)	USA	39	female	NA	sertraline, fentanyl, midazolam	no	yes	yes	yes	no
Almuwaqqat & Jokhadar	2018	J. Am. Coll. Cardiol., 71(11)	USA	31	female	depression	paroxetine, fluoxetine, amitriptyline	no	no	yes	no	no
Alnwick	2008	Physical Therapy, 88(6)	USA	42	female	depression	citalopram	no	yes	no	no	no
Alotaibi et al	2021	Clinical Neuropharmacology, 44(2)	Saudi Arabia	45	male	NA	mirtazapine, maprotiline linezolid, carbamazepine, fentanyl	yes	yes	yes	yes	no
Al-Radideh et al	2021	Cureus, 13(4)	USA	20	female	posttraumatic stress disorder	paroxetine, isotretinoin	yes	yes	yes	yes	no
Altman & Jahangiri	2010	Anesth. Analg, 110(2)	USA	44	female	major depression	duloxetine, fentanyl, lithium, clonazepam, ondansetron	no	yes	yes	yes	no
Altman & Manos	2007	Psychosomatics, 48(4)	USA	44	female	depression	citalopram, pethidine, hydromorphone, hydrocodone, promethazine, paracetamol	no	yes	yes	no	no
Ambarapu et al	2022	Annals of Indian Academy of Neurology, 25(5)	India	23	male	bipolar disorder	tranylcypromine, clomipramine, tryptophan	yes	no	yes	yes	no
Ameen & Praharaj	2013	J. Neuropsychiatry Clin. Neurosci, 25(1)	India	35	male	obsessive-compulsive disorder	sertraline, clomipramine lithium, risperidone	yes	yes	no	no	no
Aminiahidashti et al	2016	Chin. Med. J, 129(7)	Iran	23	male	NA	tramadol	yes	yes	yes	no	no
Ang et al	2015	J. Am. Geriatr. Soc, 63	USA	82	female	depression	duloxetine, tramadol, gabapentin	yes	yes	yes	no	no
Ankireddypalli et al	2020	Endocr. Pract, 26	USA	57	female	depression	amitriptyline, levocetirizine	no	yes	yes	yes	no

Ansermot et al	2014	Journal of Clinical Psychopharmacology, 34(4)	Switzerland	38	female	posttraumatic stress disorder	escitalopram, mirtazapine	yes	yes	no	no	no
Armitage et al	2015	Emergency Medicine Australasia, 28(1)	Australia	19	female	depression	amitriptyline, citalopram, pantoprazole, fentanyl, sevoflurane, propofol, midazolam, ondansetron, cefazolin, dexamethasone, parecoxib, bupivacaine, rocuronium	no	no	yes	yes	no
Atasoy et al	2008	Eur. Neuropsychopharmacol, 18	Turkey	23	female	major depressive disorder	venlafaxine, mirtazapine	yes	yes	yes	no	no
Attar-Herzberg et al, case 1	2009	The Israel Medical Association Journal, 11(6)	Israel	19	female	anxiety disorder	fluoxetine, metoclopramide,	yes	yes	yes	no	no
Attar-Herzberg et al, case 2	2009	The Israel Medical Association Journal, 11(6)	Israel	20	female	severe panic attacks	paroxetine, methylphenidate	yes	yes	yes	no	no
Attar-Herzberg et al, case 3	2009	The Israel Medical Association Journal, 11(6)	Israel	40	female	severe depression	phenelzine, fluoxetine	yes	yes	yes	yes	no
Attar-Herzberg et al, case 4	2009	The Israel Medical Association Journal, 11(6)	Israel	42	female	anxiety disorder	venlafaxine, metoclopramide	yes	yes	yes	no	no
Attar-Herzberg et al, case 5	2009	The Israel Medical Association Journal, 11(6)	Israel	50	female	severe depression	citalopram, fentanyl	yes	yes	yes	yes	no
Attar-Herzberg et al, case 6	2009	The Israel Medical Association Journal, 11(6)	Israel	64	female	NA	paroxetine, fentanyl	yes	yes	yes	no	no
Attar-Herzberg et al, case 7	2009	The Israel Medical Association Journal, 11(6)	Israel	74	female	depression	clomipramine	yes	yes	yes	no	no
Avarello & Cottone	2002	Neurological Sciences, 23(0)	Italy	72	male	depression	not specified SSRI, nortriptyline levodopa	yes	yes	yes	yes	yes
Ayoğlu et al	2009	Anatolian J. Clin. Invest, 3(3)	Turkey	22	male	depressive episode	paroxetine, paracetamol, diazepam, chlorpheniramine, pseudoephedrine	no	yes	yes	yes	no
Bach et al	2004	American Journal of Case Reports, 15	USA	59	male	anxiety disorder	paroxetine, methylene blue, ondansetron, alfentanil,	yes	yes	yes	no	no
Bachar et al	2021	Anesthesia & Analgesia, 99(5)	USA	31	female	anxiety disorder	paracetamol, chlorpheniramine	no	yes	yes	yes	no
Baetz & Malcolm	1995	J. Med. Case Rep, 15(1)	Canada	48	male	obsessive-compulsive disorder	fluvoxamine, haloperidol, valproate, buspirone	yes	yes	yes	no	no
Baigel	2003	The Canadian Journal of Psychiatry, 40(7)	UK	60	female	depression	paroxetine, risperidone	no	no	yes	yes	no
Bakirci et al	2017	Eur. J. Anaesthesiol, 20(7)	France	51	female	depression	paroxetine, clarithromycin	no	yes	yes	no	no
Baptista et al	2012	Clin. Pharmacol, 31	France	88	female	major depressive episode	escitalopram, miconazole, timolol, l-thyroxin, timolol, travoprost	yes	yes	yes	no	no
Basta	2021	International Psychogeriatrics, 24(5)	USA	64	male	depression	sertraline, methylene blue, fentanyl	yes	yes	yes	yes	no
Batista et al, case 1	2013	Clinical Toxicology (Philadelphia, Pa.), 51(2)	Belgium	38	female	depression	venlafaxine, benzodiazepines	yes	yes	yes	yes	yes
Batista et al, case 2	2013	Clinical Toxicology (Philadelphia, Pa.), 51(2)	Belgium	35	female	cocaine addiction	venlafaxine	yes	yes	yes	yes	yes
Batista et al, case 3	2013	Clinical Toxicology (Philadelphia, Pa.), 51(2)	Belgium	46	female	NA	venlafaxine	no	no	yes	yes	no

Beatty et al	2013	Journal of Clinical Anesthesia, 25(8)	USA	47	male	depression	duloxetine, trazodone fentanyl, gabapentin, ondansetron	yes	yes	yes	no	no
Behnoush et al	2013	Int. J. Med. Toxicol. Forensic Med, 3(2)	Iran	41	male	opium addiction	naltrexone, tramadol, benzodiazepine	no	yes	yes	yes	no
Benazzi	1998	International Journal of Geriatric Psychiatry, 13(7)	Italy	75	female	chronic major depressive disorder	mirtazapine, chlorpromazine, lorazepam	no	yes	no	no	no
Benazzi	2007	Pharmacopsychiatry, 29(04)	Italy	38	female	dysthymic disorder	fluoxetine, moclobemide	no	yes	no	no	no
Bergeron et al, case 1	2005	Annals of Pharmacotherapy, 39(5)	Canada	37	male	depression	citalopram, linezolid, clonazepam, hydromorphone, olanzapine	no	yes	yes	no	no
Bergeron et al, case 2	2005	Annals of Pharmacotherapy, 39(5)	Canada	38	female	NA	venlafaxine, linezolid, gabapentin, hydromorphone	no	yes	yes	no	no
Bernard et al	2003	Clinical Infectious Diseases, 36(9)	Switzerland	81	male	NA	citalopram, linezolid, prednisone, methotrexate, methotrexate, oxazepam, digoxin, nitroglycerin, torsemide, insulin	no	yes	yes	yes	yes
Bertolín-Guillén et al	2004	European Psychiatry, 19(7)	Austria	50	male	major depression	venlafaxine, maprotiline, reboxetine s-adenosylmethionine	yes	yes	yes	no	no
Bhanji	2000	Revue Canadienne de Psychiatrie, 45(10)	Canada	43	female	depression	sertraline, hydrochlorothiazide	yes	yes	yes	no	no
Bhatara et al	1998	Annals of Pharmacotherapy, 32(4)	USA	39	female	depression	fluoxetine, venlafaxine, trazodone, cimetidine	yes	yes	yes	no	no
Bhatia et al	2015	Prim. Care Companion J. Clin. Psych, 17(3)	India	63	female	depression	sertraline, cyproheptadine	yes	yes	no	no	no
Bianconi et al	2022	Clinical Toxicology, 60(3)	France	21	male	NA	paroxetine, tramadol, lorazepam, propranolol, sotalol	yes	yes	yes	yes	no
Bijl	2004	The Netherlands Journal of Medicine, 62(9)	Netherlands	50	male	major depression	fluoxetine, meprobamate, promethazine	no	yes	yes	no	no
Binder et al	2020	Journal of Investigative Medicine High Impact Case Reports, 8	USA	25	female	depression	buspirone, oxycodone, zolpidem, lamotrigine	no	yes	yes	yes	no
Birmes et al, case 1	2003	Canadian Medical Association Journal, 168(11)	France	50	male	depression	fluoxetine, meprobamate, acepromazine	no	yes	yes	no	no
Birmes et al, case 2	2003	Canadian Medical Association Journal, 168(11)	France	50	female	NA	citalopram, prazepam, meprobamate, acepromazine	yes	yes	yes	no	no
Bodner et al	1995	Neurology, 45(2)	USA	49	male	depression	trazodone, isocarboxazid methylphenidate	yes	yes	yes	no	no
Bogdanovic et al	2005	Annals of Pharmacotherapy, 39(10)	USA	56	male	depression	fluoxetine, buspirone, olanzapine	yes	yes	yes	yes	no
Bond et al	2007	Clinical Toxicology, 45(2)	USA	18	female	attention deficit hyperactivity disorder	venlafaxine, atomoxetine	yes	no	yes	no	no
Bonetto et al	2007	Cephalalgia, 27(12)	Italy	28	female	eating disorder with binge–purge behavior	fluoxetine, st john’s wort, eletriptan	no	yes	yes	no	no
Bosak & Skolnik, case 1	2014	J. Med. Toxicol, 10(4)	USA	23	female	NA	metaxalone, tramadol, naproxen	yes	yes	yes	yes	no
Bosak & Skolnik, case 2	2014	J. Med. Toxicol, 10(4)	USA	56	female	poly-substance abuse	metaxalone, clonazepam, oxycodone	yes	yes	yes	yes	no

Bosnjak Kuharic et al	2019	Psychiatria Danubina, 3(31)	Croatia	39	male	gamma-hydroxybutyric acid dependence	mirtazapine, valproate, diazepam	yes	yes	yes	yes	no
Bostankolu et al	2015	Therapeutic Advances in Psychopharmacology, 5(2)	Turkey	20	female	autism spectrum disorder	sertraline, fluoxetine aripiprazole	yes	yes	yes	no	no
Botros et al	2016	Chest, 150(4)	USA	42	male	posttraumatic stress disorder	paroxetine, fentanyl	yes	yes	yes	yes	no
Boucher et al	2015	Clinical Toxicology, 53(4)	France	29	male	schizophrenia	NBOME compounds	no	yes	yes	yes	no
Boudier-Revéret & Chang	2021	Yeungnam University Journal of Medicine, 38(4)	South Korea	36	female	major depression	duloxetine, tramadol, pregabalin, paracetamol, triazolam	yes	yes	yes	no	no
Boulé et al	2016	Eur. Geriatr. Med, 7	France	83	male	depression	escitalopram, tramadol	no	yes	yes	no	no
Brannan et al	1994	Journal of Clinical Psychopharmacology, 14(2)	USA	38	female	depression	isocarboxazid, sertraline	no	no	yes	no	no
Brazelton et al	1997	Annals of Emergency Medicine, 30(4)	USA	21	female	bipolar disorder	nefazodone, valproate	no	no	yes	no	no
Breivik	2014	Journal of Pain & Palliative Care Pharmacotherapy, 28(2)	Norway	55	female	NA	duloxetine, tramadol, ciprofloxacin	yes	yes	NA	NA	NA
Brendel et al	2000	Annals of Emergency Medicine, 36(5)	USA	51	female	major depression	sertraline	yes	yes	yes	yes	no
Bridgeman et al	2017	Clin. Toxicol, 55(7)	USA	49	male	depression	citalopram	no	yes	yes	yes	no
Brogdon et al	2022	Journal of Addiction Medicine, 16(5)	USA	36	male	naloxone/buprenorphine therapy	venlafaxine, buprenorphine, kratom, quetiapine, naloxone	no	no	yes	no	no
Brooks	1998	Revue Canadienne de Psychiatrie, 43(6)	Canada	34	female	posttraumatic stress disorder	fluoxetine, clomipramine	no	no	no	no	no
Brown	2004	The American Journal of Emergency Medicine, 22(6)	USA	23	male	major depressive disorder	paroxetine	yes	yes	yes	no	no
Brown & Skop	1996	Annals of Pharmacotherapy, 30(2)	USA	44	male	major depression	sertraline, morphine, neomycin, metronidazole	no	yes	yes	no	no
Brubacher & Basu	2011	Journal of General Internal Medicine, 26(S1)	USA	25	female	depression	escitalopram, sumatriptan, clonazepam	yes	yes	yes	no	no
Brubacher et al	1996	Veterinary and Human Toxicology, 38(5)	USA	23	male	depression	tranylcypromine, venlafaxine	yes	yes	yes	yes	no
Brvar et al	2007	Clinical Toxicology, 45(5)	Slovenia	35	female	depression	moclobemide, sertraline, citalopram	yes	yes	yes	yes	no
Brvar et al	2010	Clin. Toxicol, 48(3)	Slovenia	42	female	depression	venlafaxine	no	no	yes	yes	NA
Bryant & Kolodchak	2004	The American Journal of Emergency Medicine, 22(7)	USA	19	male	depression	st john's wort, tryptophan, MDMA	no	yes	yes	no	no
Buciuc et al	2023	Cureus, 15(9)	USA	48	female	schizophrenia	duloxetine, mirtazapine haloperidol	yes	yes	yes	no	no
Burklin & Cummings	2018	J. Hosp. Med, 13(4)	USA	47	female	depression	duloxetine, methylene blue	yes	yes	no	no	no
Bush et al	2006	Journal of Palliative Medicine, 9(6)	USA	69	female	depression	sertraline, methadone	no	no	yes	no	no
Butler et al	2010	Progress in Neuro-Psychopharmacology and Biological Psychiatry, 34(6)	Australia	30	male	major depressive disorder	sertraline, quetiapine	yes	yes	yes	no	no

Butzkueven	1997	Australian and New Zealand Journal of Medicine, 27(5)	Australia	44	male	anxiety disorder	moclobemide, alprazolam	yes	yes	yes	yes	no
Cacodcar et al	2022	Journal of the Academy of Consultation-Liaison Psychiatry, 63	USA	20	male	major depressive episode	bupropion	yes	yes	yes	no	no
Cagigal et al	2018	Eur. Psychiatry, 48	Portugal	65	female	insomnia	trazodone, quetiapine, biperiden	yes	yes	yes	no	no
Cameron	2006	Aust. Prescr, 29(3)	New Zealand	46	male	NA	citalopram, methadone, gabapentin, dextromethorphan	yes	yes	no	no	no
Canan et al	2008	The Primary Care Companion to The Journal of Clinical Psychiatry, 10(02)	Turkey	18	female	major depressive disorder	paroxetine	no	yes	yes	no	no
Casemore & Bickley	2016	Anaesthesia, 71	UK	47	male	schizophrenia	venlafaxine, olanzapine, zopiclone, diazepam	no	no	yes	yes	no
Chamula & Can	2020	Chest, 158(4)	USA	40	male	opioid dependence	duloxetine, antibiotics, fentanyl, propofol, nafcillin	yes	yes	yes	yes	no
Chan et al, case 1	1998	Medical Journal of Australia, 169(10)	Australia	18	female	NA	clomipramine, moclobemide	yes	yes	no	no	no
Chan et al, case 2	1998	Medical Journal of Australia, 169(10)	Australia	21	male	NA	desipramine, paroxetine	yes	yes	no	no	no
Chan et al, case 3	1998	Medical Journal of Australia, 169(10)	Australia	21	female	depression	venlafaxine	yes	yes	no	no	no
Chan et al, case 4	1998	Medical Journal of Australia, 169(10)	Australia	50	female	NA	amitriptyline, nefazodone, thioridazine	no	no	no	no	no
Chan et al, case 5	1998	Medical Journal of Australia, 169(10)	Australia	32	male	reactive depression	moclobemide, venlafaxine diazepam	yes	yes	no	no	no
Chan et al, case 6	1998	Medical Journal of Australia, 169(10)	Australia	82	female	NA	fluoxetine, moclobemide	no	no	no	no	no
Chang et al	2015	Am. Geriatr. Soc, 63	USA	68	male	depression	citalopram, nortriptyline morphine, fentanyl	yes	yes	yes	no	yes
Chechani, case 1	2002	Critical Care Medicine, 30(2)	USA	35	female	posttraumatic stress disorder	fluoxetine, guaifenesin, pseudoephedrine, ethanol	no	yes	yes	yes	no
Chechani, case 2	2002	Critical Care Medicine, 30(2)	USA	35	female	NA	citalopram, ethanol	no	yes	yes	no	no
Chen et al	2013	Clin. Toxicol, 51(4)	China	30	male	NA	venlafaxine, trazodone, moclobemide, zolpidem, diazepam	no	yes	yes	yes	no
Cheng et al	2008	The American Journal of Emergency Medicine, 26(1)	China	70	female	major depressive disorder	bupropion, trazodone, quetiapine	yes	yes	yes	no	no
Cheng et al	2015	Clinical Neuropharmacology, 38(3)	Taiwan	78	male	NA	amantadine, ropinirole, clonazepam, levodopa, benserazid	yes	yes	yes	yes	no
Chhangani	2017	Neurocrit. Care, 27(2)	USA	56	male	stress-induced anxiety disorder	sertraline, quetiapine	yes	yes	yes	yes	no
Chirwa et al	2008	Clinical Medicine, 8(1)	UK	71	male	depression	fluoxetine, amitriptyline fentanyl, simvastatin, allopurinol	yes	yes	yes	yes	no
Choong & Ghiculescu	2008	Aust. Fam. Phys, 37(8)	Australia	50	male	reactive depression	venlafaxine, tramadol, oxycodone, fentanyl, clonidine	yes	yes	yes	no	no

Chopra et al	2004	The World Journal of Biological Psychiatry, 5(2)	Australia	53	male	recurrent major depressive disorder	fluoxetine, olanzapine	yes	yes	yes	no	no
Chou & Averill	2019	PM R, 11	USA	64	female	depression	bupropion, duloxetine lamotrigine, pregabalin	yes	yes	yes	no	no
Choudhry	2014	Case Reports in Clinical Medicine, 03(02)	Australia	83	female	depression	sertraline, oxycodone, ondansetron, promethazine	yes	yes	yes	yes	yes
Choudhury et al	2011	Middle East Journal of Anaesthesiology, 21(1)	India	57	female	NA	fluoxetine, metoprolol, diazepam, morphine, promethazine	yes	yes	yes	no	no
Chowdhury et al	2022	J. Gen. Intern. Med, 37	USA	40	female	bipolar disorder	duloxetine, tramadol, lurasidone, lithium, alprazolam	no	no	yes	no	no
Cinderella et al	2022	Journal of the Academy of Consultation-Liaison Psychiatry, 63	USA	42	female	depression	duloxetine, nortriptyline metoclopramide	yes	yes	yes	no	no
Claassen & Gelissen	2005	New England Journal of Medicine, 352(23)	Netherlands	72	male	NA	tranylcypromine	no	no	yes	yes	no
Clark et al	2006	Pharmacotherapy: The Journal of Human Pharmacology and Drug Therapy, 26(2)	USA	47	female	depression	sertraline, atenolol, gabapentin, linezolid, clonazepam, clopidogrel, erythropoietin, famotidine, fosinopril, insulin, nateglinid, pioglitazone, simvastatin, benzonatate	yes	yes	yes	yes	no
Clevenger & McCabe	2020	The American Journal of Emergency Medicine, 38(5)	USA	21	female	suicide attempt	vilazodone	yes	yes	yes	yes	no
Collins & Hurley	2010	Clin. Toxicol, 48(6)	USA	45	male	NA	escitalopram, methylene blue, fentanyl	yes	yes	yes	yes	no
Congrete et al	2018	Am. J. Respir. Crit. Care Med, 197	Netherlands	63	male	schizophrenia	fluoxetine, olanzapine	yes	yes	yes	yes	no
Connor	2003	Journal of the Royal Society of Medicine, 96(5)	UK	56	male	depression	venlafaxine, amoxicillin, clavulanate	no	yes	no	no	no
Conte et al	2020	Chest, 158(4)	USA	62	female	anxiety disorder	bupropion, trazodone tramadol	yes	yes	yes	yes	no
Coplan & Gorman	1993	The American Journal of Psychiatry, 150(5)	USA	28	female	depression	fluoxetine, tranylcypromine	no	yes	yes	no	no
Corcoran et al	2019	Clin. Toxicol, 57(10)	USA	59	male	NA	amitriptyline, trazodone, fluoxetine, aripiprazole, alprazolam, quetiapine	no	yes	yes	yes	no
Corkeron	1995	The Medical Journal of Australia, 163(9)	Australia	39	male	NA	sertraline, tranylcypromine	yes	yes	yes	yes	no
Correia et al	2018	BMJ Case Reports, 2018	Portugal	77	male	NA	paroxetine, fluoxetine	no	no	yes	no	no
Corsini Campioli et al	2020	J. Clin. Pharm. Ther, 45(4)	USA	63	female	major depression	escitalopram, daptomycin, linezolid, fentanyl	no	yes	no	no	no
Coster et al	2010	Journal of Clinical Psychopharmacology, 30(4)	Netherlands	43	male	depression	sertraline, indomethacin	yes	yes	yes	yes	no
Curtis et al	2019	Clin. Toxicol, 57(6)	USA	36	male	NA	fentanyl, tramadol, methadone, methamphetamine	yes	yes	yes	yes	yes
Dagtekin et al	2011	Minerva Anestesiologica, 77(1)	Germany	44	female	major depression	venlafaxine, diazepam, lamotrigine	no	yes	yes	yes	no
Daniels	1998	Emergency Medicine Journal, 15(5)	Australia	28	male	depression	venlafaxine	no	yes	yes	no	no
Dannawi	2002	Journal of Psychopharmacology, 16(4)	Lebanon	27	female	generalized anxiety disorder	st john’s wort, buspirone, tyrosine	no	no	no	no	no

Dardis et al	2012	The Neurologist, 18(4)	USA	87	male	depression	sertraline, oxcarbazepine, linezolid	yes	yes	yes	yes	yes
Das & Kumar	2021	The Primary Care Companion For CNS Disorders, 23(3)	India	27	female	major depressive disorder	vilazodone	yes	yes	yes	yes	no
Davis et al	2013	Journal of Clinical Anesthesia, 25(1)	USA	23	male	depression	fluoxetine, fentanyl, remifentanil	yes	yes	no	no	no
Day & Jeanmonod	2008	The American Journal of Emergency Medicine, 26(9)	USA	27	female	mild depression	escitalopram, cyclobenzaprine, loratadine	yes	yes	yes	no	no
De Castro Julve et al	2020	Euro. J. Hosp. Pharm. Sci. Pra, 27(1)	Spain	34	male	depression	amitriptyline, paroxetine, trazodone, enalapril, tedizolid	no	no	yes	no	no
de Dios et al	2019	Medicina Intensiva, 43(9)	Spain	42	male	depression	citalopram, tryptophan, caffeine	yes	yes	yes	yes	yes
Debellis et al	2005	Journal of Intensive Care Medicine, 20(6)	USA	56	female	depression	citalopram, linezolid, ceftazidime, acyclovir, voriconazole	no	yes	yes	yes	no
Decoutere et al	2012	International Journal of Clinical Pharmacy, 34(5)	Belgium	85	female	severe depression	venlafaxine, mirtazapine	no	yes	yes	no	no
Degner et al	2010	Pharmacopsychiatry, 43(07)	Germany	53	female	recurrent depressive disorder	fluoxetine, sertraline, tranylcypromine, ethanol, aged cheese	no	yes	yes	yes	yes
Deka	2024	J Psychiatr Pract, 30(3)	USA	78	female	major depressive disorder	mirtazapine, duloxetine, ketamine	yes	yes	yes	no	no
Demers & Malone	2001	Annals of Pharmacotherapy, 35(10)	USA	26	female	anorexia nervosa	fluvoxamine, mirtazapine	no	yes	yes	no	no
Deng & Nedorost	2009	Dermatitis: Contact, Atopic, Occupational, Drug, 20(5)	USA	NA	female	NA	not specified SSRI, sumatriptan	no	no	no	no	no
Dernbach et al	2024	The Journal of Emergency Medicine, 66(5)	USA	55	female	depression	duloxetine, sertraline black cohosh	yes	yes	yes	yes	no
DeSilva et al, case 1	2001	AIDS, 15(10)	USA	42	male	depression	fluoxetine, ritonavir, fluconazole, lamivudine, acyclovir	no	yes	yes	no	no
DeSilva et al, case 2	2001	AIDS, 15(10)	USA	49	male	major depression	fluoxetine, bupropion ritonavir, clonazepam	no	yes	yes	no	no
DeSilva et al, case 3	2001	AIDS, 15(10)	USA	32	female	major depression	fluoxetine, didanosine, stavudine, hydroxyurea, efavirenz	no	no	no	no	no
DeSilva et al, case 4	2001	AIDS, 15(10)	USA	57	male	posttraumatic stress disorder	fluoxetine, trazodone grapefruits, benazepril, trimethoprim, sulfamethoxazole	no	yes	no	no	no
DeSilva et al, case 5	2001	AIDS, 15(10)	USA	40	male	bipolar affective disorder	fluoxetine, trazodone ritonavir, lithium	yes	no	no	no	no
Deuschle et al	2017	Pharmacopsychiatry, 50(1)	Germany	45	female	bipolar ii disorder	lithium, quetiapine	no	yes	yes	no	no
Diamond et al, case 1	1998	Neurology, 51(1)	USA	25	female	NA	venlafaxine	no	yes	no	no	no
Diamond et al, case 2	1998	Neurology, 51(1)	USA	49	female	NA	venlafaxine	no	yes	no	no	no
Diamond et al, case 3	1998	Neurology, 51(1)	USA	33	male	NA	venlafaxine	yes	yes	yes	no	no
Diamond et al, case 4	1998	Neurology, 51(1)	USA	29	female	NA	venlafaxine	yes	yes	no	no	no

Dike	1997	Journal of Neurology, Neurosurgery, and Psychiatry, 62(2)	USA	76	male	recurrent depression	sertraline, amantadine, levodopa, carbidopa	yes	yes	yes	no	no
Dimellis	2002	The World Journal of Biological Psychiatry, 3(3)	Greece	31	female	major depressive disorder	mirtazapine, venlafaxine	yes	yes	no	no	no
Dizdarevic & Bremer	2017	Pain Medicine, 18(6)	USA	25	male	NA	duloxetine, oxycodone, gabapentin, lamotrigine, ondansetron	yes	yes	yes	no	no
Dizdarevic & Patel	2012	Pain Pract, 12	USA	25	male	NA	duloxetine, gabapentin, lamotrigine, tizanidine, oxycodone, paracetamol	no	yes	yes	no	no
Dosi et al	2014	BMJ Case Reports, 2014	India	19	male	NA	valproate, lithium, olanzapine, risperidone	yes	yes	yes	yes	no
Dougherty et al	2002	Annals of Pharmacotherapy, 36(10)	USA	21	female	NA	venlafaxine, amitriptyline, pethidine, fluconazole, diphenhydramine, gabapentin, diphenhydramine, fluconazole, levofloxacin, trimethoprim, sulfamethoxazole, docusate, prednisolone, oxybutynin, cyclobenzaprine, salbutamol, paracetamol	no	yes	yes	yes	no
Dudum et al	2018	Am. J. Med, 131(12)	USA	60	female	bipolar disorder	sertraline, ziprasidone	yes	yes	yes	yes	no
Duggal & Fetchko	2002	American Journal of Psychiatry, 159(4)	USA	53	male	schizophrenia	mirtazapine, olanzapine, tramadol	yes	yes	yes	no	no
Duignan et al	2020	The American Journal of Emergency Medicine, 38(8)	USA	36	male	posttraumatic stress disorder	sertraline, hydroxyzine	yes	yes	yes	no	no
Dursun et al	1993	The Lancet, 342(8868)	UK	NA	female	affective disorder	fluoxetine, carbamazepine	no	no	no	no	no
Dursun et al, case 1	1997	European Psychiatry, 12(6)	UK	29	male	major depressive disorder	sertraline, sulpiride	no	yes	no	no	no
Dursun et al, case 2	1997	European Psychiatry, 12(6)	UK	44	female	major depressive disorder	paroxetine	no	yes	no	no	no
Dursun et al, case 3	1997	European Psychiatry, 12(6)	UK	24	female	major depressive disorder	fluoxetine	no	yes	no	no	no
Duval et al	2013	Movement Disorders, 28(10)	France	75	female	major depressive episode	sertraline, rasagiline, amantadine, carbidopa	yes	yes	yes	no	no
Dvir & Smallwood	2008	General Hospital Psychiatry, 30(3)	USA	53	female	depression	fluoxetine, venlafaxine, bupropion, olanzapine, methadone	yes	yes	yes	no	no
Dy et al	2017	BMJ Case Reports, 2017	USA	63	female	depression	escitalopram, amlodipine, labetalol, naltrexone, dextromethorphan, promethazine	no	no	yes	no	no
Ebert et al, case 1	1997	European Neuropsychopharmacology, 7(1)	Germany	35	male	dysthymia	fluvoxamine	no	no	yes	no	no
Ebert et al, case 2	1997	European Neuropsychopharmacology, 7(1)	Germany	38	male	dysthymia	fluvoxamine, lithium	no	no	yes	no	no
Ebert et al, case 3	1997	European Neuropsychopharmacology, 7(1)	Germany	35	male	major depression	fluvoxamine, codeine	no	no	yes	no	no

Egberts et al	1997	International Clinical Psychopharmacology, 12(3)	Netherlands	47	male	depressive disorder	paroxetine, tramadol	yes	yes	no	no	no
Ekşi et al	2019	World Neurosurg, 126	Turkey	66	male	alcohol use disorder	tramadol, gabapentin, insulin, telmisartan, hydrochlorothiazide, amlodipine, salbutamol	no	no	no	no	no
Ellison & Lyndon	2017	Journal of Clinical Psychopharmacology, 37(5)	USA	58	female	major depressive disorder	venlafaxine, amitriptyline	yes	yes	yes	no	no
El-Okdi et al	2014	Am. J. Ther, 21(4)	USA	67	male	bipolar disorder	tramadol, ziprasidone, amantadine, rasagiline	yes	yes	yes	yes	no
Elyasi & Azizi	2017	Iran. J. Psychiatr. Behav. Sci, 11(4)	Iran	66	female	anxiety disorder	fluoxetine, buspirone	yes	yes	yes	no	no
Erdem et al	2024	Cureus, 16(4)	USA	89	female	NA	venlafaxine, oxycodone	yes	yes	yes	yes	no
Erdoğan et al	2013	J. Neurosci. Rural Pract, 4(1)	Turkey	43	female	NA	escitalopram, isoniazid	no	yes	yes	no	no
Esquivel Lopez	2013	Mov. Disord, 28	Spain	66	female	depression	duloxetine, rasagiline, carbidopa, levodopa, amantadine	yes	yes	no	no	no
Esterov et al	2023	Archives of Rehabilitation Research and Clinical Translation, 5(3)	USA	22	NA	major depressive disorder	duloxetine, bupropion amantadine, gabapentin	yes	yes	yes	yes	no
Eudaley & Hamilton	2020	J.Am. Coll. Clin. Pharm, 3(1)	USA	63	male	depression	bupropion, desvenlafaxine, trazodone, buspirone, ziprasidone	yes	yes	yes	no	no
Eudaley et al	2023	J Pharm Pract, 36(6)	USA	63	male	major depressive disorder	desvenlafaxine, trazodone, bupropion, buspirone, ziprasidone, gabapentin, kratom	yes	yes	yes	yes	no
Evans & Sebastian	2007	Emergency Medicine Journal, 24(4)	Australia	38	female	NA	paroxetine, temazepam, diphenhydramine	yes	yes	yes	yes	no
Falls & Gurrera	2014	Psychosomatics, 55(3)	USA	62	male	depression	bupropion, trazodone oxycodone, tramadol	yes	yes	yes	no	no
Farkas et al	2018	Journal of Clinical Pharmacy and Therapeutics, 43(5)	USA	25	female	depression	escitalopram, lamotrigine	yes	yes	yes	yes	no
Farooq et al	2011	Crit. Care Med, 39	USA	73	female	depression	sertraline, ifosfamide, prochlorperazine, methylene blue	yes	yes	yes	yes	no
Fernández-Ferreiro et al	2016	Actas Esp Psiquiatr, 44(5)	Spain	66	female	dysthymia	venlafaxine, mirtazapine, amoxicillin, lorazepam, clavulanate, clonazepam, quetiapine, clorazepate, lormetazepam, irbesartan, simvastatin, simvastatin, lansoprazole	no	no	yes	no	no
Ferrer-Dufol et al	1998	Journal of Toxicology: Clinical Toxicology, 36(1–2)	Australia	29	male	NA	clomipramine, moclobemide clorazepate	no	yes	yes	yes	yes
Feychting et al	2012	Clin. Toxicol, 50(4)	Sweden	46	female	depression	venlafaxine, ethanol	yes	yes	yes	no	no
Fil et al	2014	Toxicol, 52(7)	USA	23	female	NA	DMT	yes	yes	yes	no	no
Finfgeld	2004	Journal of Psychosocial Nursing and Mental Health Services, 42(2)	USA	35	female	depression	fluoxetine, st john's wort, sumatriptan	yes	yes	no	no	no

Fink	2007	Pharmacopsychiatry, 29(04)	USA	59	female	bipolar disorder with psychosis	nortriptyline, trazodone levodopa	no	yes	yes	no	no
Fischer, case 1	1995	Journal of Clinical Psychopharmacology, 15(6)	Austria	66	female	refractory depression	moclobemide	no	yes	no	no	no
Fischer, case 2	1995	Journal of Clinical Psychopharmacology, 15(6)	Austria	71	female	depression	citalopram	yes	yes	yes	no	no
Fischer, case 3	1995	Journal of Clinical Psychopharmacology, 15(6)	Austria	82	female	Alzheimer's dementia	moclobemide	yes	yes	yes	no	no
Fisher & Davis, case 1	2002	Annals of Pharmacotherapy, 36(1)	Australia	72	female	major depression	sertraline, metoclopramide, morphine, celecoxib, hydrocortisone	yes	yes	yes	no	no
Fisher & Davis, case 2	2002	Annals of Pharmacotherapy, 36(1)	Australia	32	female	major depression	venlafaxine, metoclopramide, morphine, paracetamol, indomethacin	yes	yes	yes	no	no
FitzSimmons & Metha	1999	Emergency Medicine Journal, 16(4)	UK	28	male	suicide attempt	paroxetine, moclobemide paracetamol	yes	yes	yes	yes	no
Fontaine et al	2013	Crit. Care Med, 41(12)	USA	55	male	NA	escitalopram, trazodone linezolid, fentanyl	no	no	yes	yes	yes
Francescangeli et al	2016	Am. J. Case Rep, 17	USA	67	male	anxiety disorder	duloxetine, trazodone, tramadol, methylene blue, ondansetron, fentanyl, hydromorphone, paracetamol, promethazine	no	no	yes	yes	no
François et al	1997	Intensive Care Medicine, 23(1)	France	45	female	depression	clomipramine, moclobemide	no	yes	yes	yes	no
Frank	2008	Canadian Family Physician Medecin de Famille Canadien, 54(7)	Canada	80	male	depression	paroxetine	yes	yes	yes	no	no
Freeman & Chabolla	2005	Mayo Clinic Proceedings, 80(5)	USA	36	female	depression	mirtazapine, tramadol, gabapentin, zolpidem, levothyroxine	no	yes	yes	no	no
Fugate et al, case 1	2014	Resuscitation, 85(6)	USA	58	male	NA	sertraline, tramadol, fentanyl	yes	yes	yes	yes	no
Fugate et al, case 2	2014	Resuscitation, 85(6)	USA	36	male	NA	paroxetine, fentanyl	yes	yes	yes	yes	no
Fugate et al, case 3	2014	Resuscitation, 85(6)	USA	53	male	NA	fentanyl	yes	yes	yes	yes	no
Gaffney & Schreibman	2015	Case Reports in Gastroenterology, 9(2)	USA	59	female	depression	trazodone, duloxetine fentanyl	no	no	yes	yes	no
Gallardo Borge et al	2022	Psychiatry, 65	Spain	74	female	major depressive disorder	venlafaxine, tramadol, lithium, quetiapine	no	no	yes	no	NA
Ganetsky et al	2006	Ann. Intern. Med, 144(10)	USA	31	female	depression	duloxetine, clonazepam, quetiapine	yes	yes	yes	no	no
García-Murillo et al	2010	Eur. Psychiatry, 25	Spain	59	female	bipolar disorder type ii	trazodone, venlafaxine valpromide, lorazepam	yes	yes	yes	no	no
Garel et al	2021	Journal of Psychiatry and Neuroscience, 46(3)	Canada	30	male	depression	escitalopram, mirtazapine, tryptophan, tramadol	yes	yes	no	no	no
Garret	2004	Anaesth. Intensive Care, 32(4)	Australia	37	female	NA	tramadol, nitrazepam	yes	yes	yes	yes	no
Garrett & Sweeney	2010	BMJ Case Rep, 2010	Ireland	22	male	depression	fluoxetine, mephedrone, olanzapine	yes	yes	yes	no	no
Gelener et al	2011	Clinical Neuropharmacology, 34(3)	Turkey	29	female	NA	duloxetine	yes	yes	yes	no	no

George & Godleski	1996	Biological Psychiatry, 39(5)	USA	44	male	major depression	fluoxetine, trazodone	yes	yes	yes	no	no
Giblin	2019	Chest, 156(4)	USA	58	female	narcotic dependence	duloxetine, buprenorphine, cyclobenzaprine, gabapentin, naloxone	yes	yes	yes	yes	no
Gilbert & Akamune	2020	Journal of Pharmacy Practice, 33(5)	USA	29	male	major depression	venlafaxine, ifosfamide, morphine, metoclopramide, dexamethasone	no	yes	yes	yes	no
Gillman	1995	Medical Journal of Australia, 162(10)	USA	73	female	bipolar disorder, depressive	moclobemide, nortriptyline lithium, pethidine	yes	yes	yes	no	no
Gillman & Hodgens	1998	Hum. Psychopharmacol, 13(7)	Australia	18	female	NA	paroxetine	yes	yes	yes	no	no
Gnanadesigan et al, case 1	2005	Journal of the American Medical Directors Association, 6(4)	USA	86	female	NA	sertraline, oxycodone	yes	yes	yes	no	no
Gnanadesigan et al, case 2	2005	Journal of the American Medical Directors Association, 6(4)	USA	88	female	NA	escitalopram, oxycodone	no	no	yes	no	no
Gnanadesigan et al, case 3	2005	Journal of the American Medical Directors Association, 6(4)	USA	90	female	NA	escitalopram, hydrocodone	no	no	no	no	no
Gnanadesigan et al, case 4	2005	Journal of the American Medical Directors Association, 6(4)	USA	85	female	NA	mirtazapine, tramadol	no	no	no	no	no
Go et al, case 1	2010	Dmdi, 25(1–4)	USA	52	female	depressive disorder	duloxetine, escitalopram vancomycin, daptomycin, linezolid	no	no	yes	no	no
Go et al, case 2	2010	Dmdi, 25(1–4)	USA	54	male	NA	sertraline, linezolid, caspofungin, imipenem, cilastatin	no	no	yes	yes	no
Goffin et al	2020	Journal of Rehabilitation Medicine – Clinical Communications, 3(1)	Belgium	40	female	sleep disturbances	trazodone, paroxetine baclofen	no	no	no	no	no
Gogineni et al	2013	Chest, 144(4)	USA	19	female	bipolar disorder	sertraline	no	yes	yes	yes	no
Gokcinar et al	2009	Acta Anaesthesiologica Scandinavica, 53(5)	Turkey	40	female	depression	paroxetine, fentanyl, thiopental, vecuronium, nitroglycerin, diclofenac	no	no	yes	no	no
Goldberg & Huk	1992	Psychosomatics, 33(2)	USA	74	male	major depression	trazodone, buspirone, haloperidol	no	no	yes	no	no
Gollapudy et al	2012	Journal of Clinical Anesthesia, 24(3)	USA	68	female	anxiety disorder	paroxetine, duloxetine, bupropion, gabapentin, oxycodone, tiagabine, clonazepam, propofol, fentanyl, ondansetron, hydromorphone	no	yes	yes	yes	no
Gollapudy et al	2017	Journal of Cardiothoracic and Vascular Anesthesia, 31(4)	USA	57	female	depression	citalopram, linezolid, tramadol, fluconazole	yes	yes	yes	yes	no
Gomez & Mazurkiewicz	2021	J. Gen. Intern. Med, 36	USA	21	male	bipolar depression	fluoxetine, lithium, levetiracetam, antibiotics, fentanyl	yes	yes	yes	yes	no
Gómez-Esteban et al	2009	Clinical Neuropharmacology, 32(5)	Spain	24	female	major depressive episode with psychosis	fluoxetine	yes	yes	yes	yes	yes
Gould et al	2021	BMJ Case Reports, 14(2)	UK	54	male	NA	sertraline, amitriptyline tapentadol, pregabalin, ketamine	yes	yes	yes	yes	no

Graudins et al, case 1	1998	The Journal of Emergency Medicine, 16(4)	Australia	42	female	depression	sertraline, moclobemide diazepam	yes	yes	yes	no	no
Graudins et al, case 2	1998	The Journal of Emergency Medicine, 16(4)	Australia	69	female	depression	moclobemide, paroxetine	no	yes	no	no	no
Graudins et al, case 3	1998	The Journal of Emergency Medicine, 16(4)	Australia	43	female	depression	moclobemide, paroxetine	yes	yes	yes	no	no
Graudins et al, case 4	1998	The Journal of Emergency Medicine, 16(4)	Australia	43	female	NA	venlafaxine	yes	yes	no	no	no
Gray et al	2018	Eur. Thyroid J, 7	UK	29	male	depression	fluoxetine, sertraline tramadol	no	no	yes	no	no
Gressier et al	2014	American Journal of Psychiatry, 171(8)	France	48	male	drug-resistant major depression	venlafaxine	yes	yes	yes	no	no
Grigorescu et al	2022	Journal of the Pakistan Medical Association, 72(4)	Romania	36	female	NA	tramadol, granisetron, fentanyl	no	no	yes	yes	no
Gueits & Witkin	2020	Curr. Psychiatry, 19(3)	USA	41	female	major depressive disorder	fluoxetine, warfarin, diphenhydramine, ketamine	no	yes	yes	no	no
Gunnervik & Lindeman	2020	Clin. Toxicol, 58(6)	Sweden	70	female	suicide attempt	citalopram, amlodipine, oxazepam, candesartan, propiomazine	yes	yes	yes	yes	no
Guo et al	2009	British Journal of Anaesthesia, 103(3)	China	41	male	NA	pethidine	yes	yes	yes	no	no
Guo et al	2018	American Journal of Case Reports, 19	USA	50	female	NA	duloxetine, tramadol, ondansetron, promethazine	no	yes	yes	no	no
Guzé & Baxter	1986	Journal of Clinical Psychopharmacology, 6(2)	USA	30	female	rapid cycling bipolar disorder	isocarboxazid, tryptophan lithium alprazolam, levothyroxine	yes	yes	yes	no	no
Haacker et al	2018	Psychosomatics, 59(5)	USA	42	male	attention deficit hyperactivity disorder	paroxetine, bupropion, lisdexamfetamine, metoprolol, enoxaparin	yes	yes	yes	no	no
Hachem et al, case 1	2003	Clinical Infectious Diseases, 37(1)	USA	36	male	NA	sertraline, linezolid, morphine, thalidomide	no	yes	yes	no	no
Hachem et al, case 1	2003	Clinical Infectious Diseases, 37(1)	USA	56	female	depression	citalopram, linezolid	yes	yes	yes	no	yes
Hadikusumo & Ng	2009	The Australian and New Zealand Journal of Psychiatry, 43(6)	Australia	70	female	major depressive disorder	duloxetine, lithium	yes	yes	yes	no	no
Haffenden & Patel	2018	Prog. Neurol. Psychiatry, 22(1)	UK	53	male	major depression	citalopram, tramadol, ondansetron	yes	yes	yes	no	no
Hagahmed et al	2017	Clin. Toxicol, 55(7)	USA	54	male	obsessive-compulsive disorder	fluoxetine, lithium, clozapine, oseltamivir	yes	yes	yes	yes	no
Haggerty & Curtis	2010	Clinical Toxicology, 48(3)	USA	40	female	NA	carisoprodol, oxycodone, oxycodone, paracetamol	yes	yes	yes	no	no
Hammond et al	2022	Obstetric Med, 15(1)	USA	36	female	bipolar disorder type i	sertraline, lurasidone, buspirone, clonazepam	yes	yes	yes	no	no
Hanekamp et al	2005	The Netherlands Journal of Medicine, 63(8)	Netherlands	21	female	depression	venlafaxine	no	yes	yes	yes	no
Hanna & Clark	2014	A A Case Rep, 2(9)	USA	62	male	depression	escitalopram, methylene blue, fentanyl	no	yes	yes	yes	no
Hansbauer & Strauss	2021	Eur. J. Psychiatry, 35(3)	Germany	41	male	moderate depression	mirtazapine, duloxetine	yes	yes	yes	yes	no
Harada et al	2017	Internal Medicine, 56(6)	Japan	40	female	bipolar disorder	mirtazapine, metoclopramide, lamotrigine, aripiprazole, lorazepam	yes	yes	yes	no	no

Haringer & Warner	2016	Neurology, 86(16)	USA	68	female	depression	paroxetine, risperidone, dexamphetamine	yes	yes	yes	no	no
Harmouche et al	2018	Clinical Toxicology, 56(6)	USA	22	male	bipolar disorder	clozapine	no	yes	yes	yes	no
Harmouche et al	2019	Clin. Toxicol, 57(6)	USA	20	female	depression	metaxalone	yes	yes	yes	yes	yes
Hasani et al	2019	Anesthesia: Essays and Researches, 13(1)	India	82	female	major depressive disorder	sertraline, linezolid	yes	yes	yes	yes	no
Hashmi et al	2019	American Journal of Therapeutics, 26(6)	USA	49	male	major depressive disorder	citalopram, methylene blue, hydralazine, carvedilol	no	yes	yes	yes	no
Haslett & Kumar	2002	Psychiatry and Clinical Neurosciences, 56(5)	New Zealand	47	female	bipolar affective disorder	citalopram, olanzapine, lithium	yes	yes	yes	no	no
Hassan et al	2023	The American Journal of the Medical Sciences, 365	USA	25	male	methamphetamine abuse	methamphetamine	yes	yes	yes	yes	no
Hébant et al	2016	Revue Neurologique, 172(12)	France	72	male	major depressive disorder	paroxetine, rasagiline, rotigotine, levodopa, entacapone, carbidopa, alprazolam	no	yes	yes	no	no
Heisler et al	1996	Annals of Pharmacotherapy, 30(1)	USA	46	male	major depression	phenelzine, venlafaxine, valproate	yes	yes	yes	yes	no
Hencken et al	2016	Journal of Cardiac Surgery, 31(4)	USA	50	female	depression	citalopram, methylene blue	yes	yes	yes	no	no
Heritier Barras et al	2010	Journal of Neurology, Neurosurgery & Psychiatry, 81(12)	Switzerland	77	female	depression	clomipramine, methylene blue, quinapril, fentanyl	yes	yes	yes	yes	no
Hernández et al	2002	Annals of Pharmacotherapy, 36(4)	Spain	75	male	major depressive disorder	mirtazapine, salbutamol, ipratropium bromide, nimodipine	yes	yes	yes	no	no
Hillman et al	2015	Pharmacotherapy, 35(1)	USA	36	male	bipolar disorder	fentanyl, methadone, oxycodone, haloperidol	yes	yes	yes	yes	no
Himmighoffen et al	2011	Pharmacopsychiatry, 44(02)	Switzerland	60	female	recurrent depressive disorder	duloxetine, olanzapine, zolpidem, pregabalin	yes	yes	yes	no	no
Hinds et al	2000	Journal of Neurology, 247(10)	UK	48	female	severe depression	nortriptyline, selegiline, trihexyphenidyl	yes	yes	yes	yes	no
Hodgman et al	1997	Human & Experimental Toxicology, 16(1)	USA	60	female	depression	tranylcypromine, venlafaxine liothyronine	yes	yes	yes	yes	no
Hodnett et al	2006	Ir. J. Psychol. Med, 23(2)	Ireland	63	male	anxiety disorder	venlafaxine, biperiden, sulpirid, lorazepam	yes	yes	yes	yes	no
Hoes	1996	Pharmacopsychiatry, 29(02)	Netherlands	70	female	depression	clomipramine, tranylcypromine	no	yes	yes	no	no
Hoffer et al	2023	Cureus, 15(11)	USA	42	male	schizophrenia,	trazodone, ondansetron, olanzapine, risperidone, haloperidol	yes	yes	yes	no	no
Honoré et al	2018	Acta Neurologica Belgica, 118(1)	Belgium	66	male	NA	sertraline, methylene blue	no	no	yes	no	no
Horn & Hansten	2018	Pharm. Times, 2018	USA	39	male	NA	darunavir, methadone, linezolid, amoxicillin, lamivudine, ceftriaxone, clindamycin, tenofovir, ritonavir	yes	yes	yes	no	NA
Houlihan	2004	Annals of Pharmacotherapy, 38(3)	USA	47	male	major depressive disorder	venlafaxine, tramadol	yes	yes	yes	no	no
Hsieh et al	2024	American Journal of Therapeutics, 31(3)	China	67	male	alcohol dependence	paroxetine, risperidone, quetiapine	yes	yes	yes	no	no

Hudd et al	2020	Journal of Pharmacy Practice, 33(2)	USA	21	female	generalized anxiety disorder	paroxetine, fluoxetine hydroxyzine, ondansetron	no	no	yes	no	no
Huffman & Bacon	2022	Critical Care Medicine, 50(1)	USA	43	male	bipolar disorder	duloxetine, buprenorphine, lamotrigine, naloxone	yes	yes	yes	yes	no
Huffman & Bacon	2022	Crit. Care Med, 50(1)	Romania	21	male	severe depression	trazodone, olanzapine, valproate, aripiprazole, lorazepam	yes	yes	yes	yes	no
Hundal et al	2021	Crit. Care Med, 49(1)	USA	22	female	depression	fluoxetine, oxycodone	yes	yes	yes	no	no
Hunter et al	2006	Anesth. Analg, 102(5)	Argentina	66	female	NA	tranylcypromine, trifluoperazine, remifentanil	yes	yes	yes	yes	no
Huska et al	2007	CNS Spectrums, 12(4)	USA	24	female	NA	escitalopram	yes	yes	yes	yes	no
Huskey et al	2013	Annals of Pharmacotherapy, 47(7–8)	USA	57	female	depression	milnacipran, fluoxetine, doxepin, tramadol, alprazolam, zolpidem	no	yes	yes	no	no
Hussain et al	2016	Crit. Care Med, 44(12)	Netherlands	60	female	generalized anxiety disorder	escitalopram, methylene blue	yes	yes	yes	yes	no
Hussein et al	2022	J. Invest. Med, 70(7)	USA	48	female	major depression	duloxetine, escitalopram scopolamine, promethazine	yes	yes	yes	no	no
Hwang & Shim	2019	Am. J. Respir. Crit. Care Med, 199(9)	South Korea	51	female	NA	isoniazid, lorazepam, metoclopramide	no	yes	yes	no	no
Igneri et al	2013	Crit. Care Med, 41(12)	USA	52	female	NA	citalopram, metaxalone	yes	yes	yes	yes	no
Inoue et al	2023	Cureus, 15(12)	Japan	53	male	depression	paroxetine, trazodone flunitrazepam, zopiclone, quetiapine, aripiprazole	yes	yes	yes	yes	yes
Insel et al, case 1	1982	The American Journal of Psychiatry, 139(7)	USA	30	male	NA	clomipramine, clorgyline	no	no	yes	no	no
Insel et al, case 2	1982	The American Journal of Psychiatry, 139(7)	USA	35	female	obsessive-compulsive disorder	clomipramine, clorgyline	yes	yes	yes	no	no
Isaac et al	2022	Crit. Care Med, 50(1)	USA	34	female	depression	venlafaxine, tianeptine	yes	yes	yes	no	no
Isenberg et al	2008	Am. J. Emerg. Med, 26(7)	USA	54	male	NA	doxepin, amitriptyline, buprenorphine, naloxone, ramipril, doxazosin, tolterodine, gabapentin, metformin, repaglinide, morphine	yes	yes	yes	yes	no
Ishida et al	2020	Clinical Neuropharmacology, 43(3)	Japan	75	female	somatoform disorder	milnacipran, duloxetine perospirone, bromazepam	yes	yes	yes	yes	no
Ishii et al	2008	Psychiatry and Clinical Neurosciences, 62(2)	Japan	62	female	major depressive episode	sertraline, methylphenidate	no	yes	yes	no	no
Izdes et al	2014	A A Case Rep, 2(9)	Turkey	31	male	depression	escitalopram, methylene blue, esmolol	yes	yes	yes	yes	no
Jaber et al	2006	The American Journal of Medicine, 119(4)	USA	36	female	mild anxiety	paroxetine, clarithromycin	no	no	no	no	no
Jahr et al	1994	Anesthesia & Analgesia, 79(1)	USA	18	female	depression	sertraline, lidocaine, fentanyl, midazolam, cefamandole	no	no	yes	yes	no
Jain et al	2007	Germ. J. Psychiatry, 10(3)	India	56	male	bipolar affective disorder	sertraline, trazodone, escitalopram, lithium, amlodipine, phenytoin	yes	yes	yes	yes	no
Jang et al	2019	Medicine, 98(13)	South Korea	55	male	NA	venlafaxine, tianeptine, methylphenidate, ropinirole, levodopa, bromocriptine, carbidopa	no	yes	yes	no	no
Jayadevi	2021	Indian J. Crit. Care Med, 25	India	30	male	NA	tramadol, ondansetron, metoclopramide	yes	yes	yes	no	no

Jellestad et al	2016	International Journal of Medical and Pharmaceutical Case Reports, 7(5)	Switzerland	45	female	posttraumatic stress disorder	paroxetine, trazodone, bupropion, pregabalin, methadone, paliperidone	yes	yes	yes	no	no
Jeon et al	2017	Clinical Neuropharmacology, 40(4)	South Korea	55	male	NA	atomoxetine, methylphenidate, levodopa, benserazide	yes	yes	yes	no	no
Jin & Stokes	2021	Journal of Medical Case Reports, 15(1)	Australia	30	female	depression	fluvoxamine, sumatriptan, pregabalin, tranexamic acid	yes	yes	yes	no	no
Jindal et al	2019	Indian J. Crit. Care Med, 23(8)	India	60	male	major depressive disorder	escitalopram, paroxetine, metoprolol, olmesartan, acetylsalicylic acid, atorvastatin, clonazepam, metformin, metformin	yes	yes	yes	yes	no
John et al	1997	Annals of Emergency Medicine, 29(2)	Canada	51	female	bipolar affective disorder	nefazodone, paroxetine, valproate	yes	yes	yes	yes	no
John et al, case 1	2013	Headache, 53(9)	USA	57	female	depression	bupropion, mirtazapine, citalopram, buprenorphine	no	yes	yes	yes	no
John et al, case 2	2013	Headache, 53(9)	USA	45	female	depression	escitalopram, hydromorphone, dihydroergotamine	yes	yes	yes	yes	yes
Johnson et al	2012	J. Intensive Care Soc, 13(3)	UK	75	female	NA	citalopram, methylene blue, tramadol, fentanyl, ondansetron	yes	yes	yes	yes	no
Jones	2004	Journal of Antimicrobial Chemotherapy, 54(1)	Australia	85	male	depression	venlafaxine, linezolid	yes	yes	yes	no	no
Kabil et al	2024	A&A Practice, 18(6)	USA	32	male	NA	propofol, remifentanil	yes	yes	yes	yes	no
Kaneda et al	2001	General Hospital Psychiatry, 23(2)	Japan	51	female	bipolar ii, currently depressed	clomipramine, sulpiride, bromazepam, thioridazine	no	yes	yes	no	no
Kaneda et al	2002	The International Journal of Neuropsychopharmacology, 5(01)	Japan	23	female	depression	paroxetine	yes	yes	yes	no	no
Káňová et al	2017	Ceska Slov. Neurol. Neurochir, 80(6)	Czech Republic	51	female	NA	paroxetine, trazodone tramadol, chlorprothixene, bromazepam	yes	yes	yes	yes	no
Kapadia et al	2016	Urol. Case Rep, 9	USA	74	female	depression	fluoxetine, duloxetine, methylene blue, fentanyl, ondansetron, ropinirole, levodopa, carbidopa, entacapone	no	yes	yes	yes	no
Kapil et al	2019	Crit. Care Med, 47(1)	USA	36	male	depression	sertraline, aripiprazole, propofol, midazolam, hydromorphone, pethidine	no	yes	yes	yes	no
Karki & Masood, case 1	2003	Annals of Pharmacotherapy, 37(3)	USA	86	male	depression	paroxetine, risperidone, simvastatin, quinapril	yes	yes	yes	no	yes
Karki & Masood, case 2	2003	Annals of Pharmacotherapy, 37(3)	USA	78	female	bipolar ii disorder, currently depressed	venlafaxine, paroxetine risperidone, isosorbide dinitrate lisinopril, glyburide	yes	yes	yes	no	no
Karunatilake & Buckley	2006	Annals of Pharmacotherapy, 40(1)	Australia	70	female	depression	fluvoxamine, oxycodone, diclofenac, raloxifene, calcium carbonate, diltiazem, simvastatin	yes	yes	yes	no	no
Kata et al	2022	Chest, 162(4)	USA	53	female	depression	venlafaxine	no	yes	yes	yes	no
Kaufman et al	2006	Annals of Clinical Psychiatry, 18(3)	USA	23	female	bipolar i disorder	venlafaxine, topiramate, valproate, risperidone, carbamazepine	no	no	yes	yes	no
Keltner	2009	Perspectives in Psychiatric Care, 30(4)	USA	55	female	panic disorder	phenelzine, sertraline	yes	yes	yes	yes	yes

Kesavan & Sobala	1999	Journal of the Royal Society of Medicine, 92(9)	UK	31	female	NA	fluoxetine, tramadol	no	yes	yes	no	no
Khan et al	2007	Annals of The Royal College of Surgeons of England, 89(2)	UK	66	female	depression	clomipramine, alverine citrate	yes	yes	yes	no	no
Khan et al	2022	Cureus, 14(3)	USA	37	female	bipolar disorder	mirtazapine, trazodone oxycodone, fentanyl, paracetamol	yes	yes	yes	yes	no
Khan et al	2024	Case Reports in Psychiatry, 2024(1)	USA	34	female	major depressive disorder	duloxetine, buspirone, clonazepam	no	yes	yes	yes	no
Khoury et al	2018	BMJ Case Reports, 2018	USA	67	male	generalized anxiety disorder	escitalopram, trazodone linezolid, clonazepam	yes	yes	yes	no	no
Kim et al	2007	Journal of Clinical Neurology, 3(3)	South Korea	19	male	mental retardation	sertraline, risperidone, quetiapine, propranolol, benztropine, clonazepam	yes	yes	yes	no	no
Kinoshita et al	2011	Geriatr. Gerontol. Int, 11(1)	Japan	77	male	NA	dextromethorphan	yes	yes	yes	no	no
Kinzie & Meltzer-Brody	2005	General Hospital Psychiatry, 27(3)	USA	40	male	schizoaffective disorder	citalopram, clozapine, ziprasidone, valproate	yes	yes	yes	no	no
Kirk et al	2016	Crit. Care Med, 44(12)	USA	61	male	NA	paroxetine, buprenorphine, naloxone	yes	yes	yes	no	no
Kirschner & Donovan, case 1	2010	J. Emerg. Med, 38(4)	USA	59	female	NA	escitalopram, trazodone fentanyl, oxycodone	yes	yes	yes	yes	no
Kirschner & Donovan, case 2s	2010	J. Emerg. Med, 38(4)	USA	46	female	depression	sertraline, fentanyl, midazolam	yes	yes	yes	yes	no
Kitson & Carr	2005	Anaesthesia, 60(9)	UK	79	female	depression	amitriptyline, tramadol, rofecoxib, morphine, dextropropoxyphene, paracetamol	yes	yes	yes	yes	yes
Klaassen et al, case 1	1998	Psychiatry Research, 79(3)	Netherlands	46	female	major depression	meta-chlorophenylpiperazine, bromazepam	no	yes	yes	no	no
Klaassen et al, case 2	1998	Psychiatry Research, 79(3)	Netherlands	28	male	obsessive-compulsive disorder	meta-chlorophenylpiperazine	yes	yes	yes	no	no
Klaassen et al, case 3	1998	Psychiatry Research, 79(3)	Netherlands	28	male	social phobia and dysthymia	meta-chlorophenylpiperazine	no	yes	yes	no	no
Kłys et al	2009	Forensic Science International, 184(1–3)	Poland	21	female	depression	moclobemide, venlafaxine, cocaine	no	yes	yes	yes	yes
Klysner et al	1995	The Lancet, 346(8985)	Denmark	43	male	depression	isocarboxazid, venlafaxine	yes	yes	yes	no	no
Klysner et al	2014	Case Reports in Psychiatry, 2014	Denmark	31	female	major depressive disorder	fluoxetine, quetiapine	no	no	yes	no	no
Koekkoek & Tjan	2017	Netherlands Journal of Critical Care, 25(3)	Netherlands	55	female	chronic depression	sertraline, olanzapine, lorazepam, paracetamol	no	no	yes	yes	yes
Kohen et al, case 1	2007	CNS Spectrums, 12(8)	USA	69	male	depression with psychosis	sertraline, trazodone risperidone metoprolol	yes	yes	yes	no	no
Kohen et al, case 2	2007	CNS Spectrums, 12(8)	USA	72	female	depression with psychotic features	phenelzine, quetiapine	yes	yes	yes	no	no
Kojima et al	1993	The American Journal of Psychiatry, 150(12)	Japan	59	male	depression	clomipramine, lithium, levomepromazine, flunitrazepam	yes	yes	yes	no	no
Kolecki	1997	The Journal of Emergency Medicine, 15(4)	USA	34	male	major depression	venlafaxine, phenelzine	yes	yes	yes	no	no

Kolecki	1997	Journal of Toxicology: Clinical Toxicology, 35(2)	USA	26	male	depression	venlafaxine	yes	yes	yes	yes	no
Kotwal & Cutrona	2015	Med, 2015	USA	24	female	bipolar disorder	aripiprazole, cocaine, lamotrigine	yes	yes	yes	no	no
Kovacic et al	2009	Eur. J. Psychiatry, 23(1)	Croatia	28	female	depression	fluvoxamine, paracetamol, propyphenazone, caffeine	yes	yes	yes	no	no
Kraai & Seifert	2015	Journal of Medical Toxicology, 11(2)	USA	35	female	depression	citalopram, THC	no	yes	yes	yes	yes
Krishnamoorthy et al	2016	Journal of the Intensive Care Society, 17(3)	UK	44	male	NA	venlafaxine, quetiapine	yes	yes	yes	yes	no
Kudo et al, case 1	1997	Psychiatry and Clinical Neurosciences, 51(1)	Japan	23	female	depression	clomipramine	yes	yes	yes	no	no
Kudo et al, case 2	1997	Psychiatry and Clinical Neurosciences, 51(1)	Japan	42	female	depression	clomipramine	no	no	no	no	no
Kuisma	1995	Annals of Emergency Medicine, 26(1)	Finland	26	female	NA	moclobemide, clomipramine, amitriptyline	no	no	yes	yes	yes
Kulkarni & Kulkarni	2013	Indian Journal of Psychological Medicine, 35(4)	India	65	female	depressive disorder	escitalopram, linezolid	yes	yes	yes	yes	no
Kumai et al	2020	Case Reports in Oncology, 13(1)	Japan	47	female	NA	duloxetine, methadone, oxycodone	no	yes	yes	no	no
Kumar et al	2011	Indian J. Psychiatry, 53(4)	India	50	male	recurrent depressive disorder	sertraline, venlafaxine	no	yes	yes	no	no
Kumar Yada et al, case 1	2021	Clin. Neurophysiol, 132(8)	India	69	male	NA	multiple antipsychotics	no	no	yes	no	no
Kumar Yada et al, case 1	2021	Clin. Neurophysiol, 132(8)	India	70	male	NA	multiple antipsychotics	no	no	yes	no	no
Kumar Yada et al, case 1	2021	Clin. Neurophysiol, 132(8)	India	58	male	psychosis	escitalopram, amitriptyline amantadine, rasagiline, olanzapine, clonazepam	no	no	yes	no	no
Kung & Ng	2007	Hong Kong J. Emerg. Med, 14(1)	China	29	male	NA	tramadol, dextromethorphan, theophylline, domperidone	yes	yes	yes	no	no
Kuwana et al	2024	Journal of Personalized Medicine, 14(3)	Japan	NA	male	depression	dextromethorphan, flunitrazepam	yes	yes	yes	yes	no
Laban et al	2021	Rev. Neurol, 177(8)	France	62	male	depression	fluoxetine, oxazepam	no	no	yes	no	no
Lad et al	2020	Indian J. Forensic Med. Toxicol, 14(3)	India	82	male	adjustment disorder	escitalopram, zolpidem, voglibose SR, amlodipine	no	no	yes	yes	no
Lalkin et al	1998	Can. J. Clin. Pharmacol, 5(1)	Canada	27	male	personality disorder	paroxetine, perphenazine	yes	yes	yes	yes	no
Lam et al	2012	Hum. Exp. Toxicol, 31(4)	China	21	female	suicide attempt	sibutramine, phenolphthalein, caffeine	no	yes	yes	yes	no
Lamberg & Gordin	2014	Pain Medicine, 15(8)	USA	41	female	anxiety disorder	amitriptyline, duloxetine carisoprodol, cyclobenzaprine, gabapentin, tramadol	yes	yes	no	no	no
Lancaster et al	2022	Cardiol, 2022	USA	26	female	autism spectrum disorder	fluoxetine, levetiracetam, buspirone, zonisamide	no	yes	yes	no	no
Lange-Asschenfeldt et al	2002	Journal of Clinical Psychopharmacology, 22(4)	Germany	44	female	dysthymia	fluoxetine, tramadol	yes	yes	yes	no	no
Lantz et al, case 1	1998	International Journal of Geriatric Psychiatry, 13(5)	USA	78	female	recurrent major depression	paroxetine, tramadol	no	yes	yes	no	no

Lantz et al, case 2	1998	International Journal of Geriatric Psychiatry, 13(5)	USA	88	female	bipolar disorder	paroxetine, tramadol, valproate, quinapril, aspirin	no	yes	yes	no	no
Lappin & Auchincloss	1994	New England Journal of Medicine, 331(15)	USA	26	female	depression	isocarboxazid, sertraline, trazodone	yes	yes	yes	no	no
Larson et al	2015	Journal of Clinical Anesthesia, 27(3)	USA	60	male	seasonal affective disorder	fluoxetine, methylene blue, fentanyl	yes	yes	yes	no	no
Lattanzi et al	2008	Bipolar Disorders, 10(5)	Italy	39	female	bipolar disorder type ii	phenelzine	yes	yes	yes	no	no
Lavery et al	2001	Psychosomatics, 42(5)	USA	45	male	schizoaffective disorder	sertraline, bupropion, trazodone, linezolid, lithium, metronidazole	no	yes	yes	no	no
Lavry & Mostin	2019	Clin. Toxicol, 57(6)	Belgium	52	female	depression	paroxetine, bisoprolol, atorvastatin, alprazolam, methylprednisolone, zoledronate, methotrexate, etanercept, rivaroxaban	no	no	yes	yes	no
Lawver et al	2010	Military Medicine, 175(12)	Iraq	33	male	adjustment disorder	fluoxetine, sertraline, diazepam	yes	yes	yes	no	no
Lee et al	2009	Psychosomatics, 50(6)	USA	61	male	major depressive disorder	venlafaxine, methadone, hydromorphone, oxycodone	yes	yes	yes	no	no
Lejoyeux et al	1992	The American Journal of Psychiatry, 149(10)	France	30	male	major depression	clomipramine	yes	yes	yes	yes	no
Lenzi et al	1993	Pharmacopsychiatry, 26(3)	Italy	34	male	obsessive-compulsive disorder	fluvoxamine	no	yes	yes	no	no
Levine et al	2011	Journal of Medical Toxicology, 7(4)	USA	59	female	depression	milnacipran, paroxetine methylphenidate, carisoprodol, methadone	yes	yes	yes	yes	no
Li & Mi	2023	Cureus, 15(6)	China	69	female	depressive disorder	sertraline, duloxetine, levamlodipine	yes	yes	yes	no	no
Li & Mi	2023	Cureus, 15(6)	China	69	female	depressive episode	sertraline, duloxetine, levamlodipine	yes	yes	yes	no	no
Liau et al	2006	Psychiatry and Clinical Neurosciences, 60(1)	China	69	female	major depressive disorder	venlafaxine trazodone	yes	yes	no	no	no
Liberek et al	2006	Therapies, 61(6)	Switzerland	41	male	bipolar i disorder	venlafaxine, lithium, valproate	yes	yes	yes	no	no
Lim & Arshad	2020	American Journal of Case Reports, 21	USA	79	male	NA	sertraline	no	yes	yes	yes	no
Lipscomb et al	2022	Crit. Care Med, 50(1)	USA	55	female	depression	amitriptyline, duloxetine, methylene blue	yes	yes	yes	yes	NA
Little et al	2018	American Journal of Case Reports, 19	USA	24	male	depression	fluoxetine, trazodone, bupropion, cyproheptadine, olanzapine, risperidone	yes	yes	yes	yes	no
Liu et al	2009	Journal of the American Geriatrics Society, 57(12)	USA	85	male	bipolar disorder, depression	duloxetine, fluoxetine	no	no	no	no	no
Liu et al	2024	Clinical Case Reports, 12(5)	China	85	female	NA	linezolid	no	no	yes	no	no
Liu et al., case 1	2019	Medicine, 98(19)	China	49	male	depression	paroxetine, citalopram olanzapine, donepezil, estazolam	yes	yes	yes	yes	no
Liu et al., case 2	2019	Medicine, 98(19)	China	49	male	depression	citalopram, olanzapine, donepezil	yes	yes	yes	yes	no
Lizer & Masters	2006	J. Pharm. Technol, 22(2)	USA	23	female	bipolar depression	venlafaxine, methylphenidate	no	yes	yes	yes	no
Lorenzini et al	2012	AIDS, 26(18)	Switzerland	46	female	depressive disorder	escitalopram, darunavir, ritonavir, esomeprazole	yes	yes	yes	no	no

Lu et al	2006	Journal of the Formosan Medical Association, 105(1)	China	68	male	generalized anxiety disorder	venlafaxine, buspirone, amantadine, clonazepam	no	yes	yes	yes	no
Lynch & Dale	2019	J. Am. Geriatr. Soc, 67	USA	88	female	dementia	sertraline, trazodone clonazepam, valproate, lorazepam, tramadol	yes	yes	yes	no	no
Ma et al	2013	Psychiatry and Clinical Neurosciences, 67(6)	China	98	female	depression	citalopram, amiodarone, linezolid	yes	yes	yes	yes	no
Macovei et al	2010	Clin. Toxicol, 48(3)	Romania	46	male	bipolar depression	venlafaxine	no	no	yes	yes	no
Madan et al	2023	Case Reports in Psychiatry, 2023	USA	32	male	autism spectrum disorder	sertraline	yes	yes	yes	no	no
Maddry et al	2013	The American Journal of Emergency Medicine, 31(2)	USA	19	male	NA	citalopram, aripiprazole, lamotrigine, lorazepam	yes	yes	yes	yes	no
Madsen & Curtis	2010	Clin. Toxicol, 48(3)	USA	22	female	NA	doxylamine, diphenhydramine, paracetamol, ethanol	no	yes	yes	yes	no
Mahlberg et al	2004	American Journal of Psychiatry, 161(6)	Germany	70	female	recurrent depressive disorder	citalopram, tramadol	no	yes	no	no	no
Majithia & Stearns	2006	The Journal of Laryngology & Otology, 120(2)	UK	52	female	depression	venlafaxine, methylene blue	no	no	yes	no	no
Maktabi et al	2024	Mental Health Clinician, 14(1)	USA	56	male	depression, unspecified mood disorder	vortioxetine, trazodone lamotrigine, lurasidone, oxycodone	no	yes	yes	yes	no
Malik & Junglee	2015	Case Reports in Medicine, 2015	UK	27	female	depression	phenelzine, mirtazapine, propranolol	yes	yes	yes	yes	no
Malik & Kumar	2012	Clin. Med. Insights. Case. Rep., 5	USA	20	male	depression	escitalopram, quetiapine, clonazepam, cocaine, oxycodone, paracetamol	yes	yes	yes	yes	no
Malik et al	2020	Case Reports in Neurology, 12(1)	USA	41	female	bipolar disorder	venlafaxine, buspirone	yes	yes	yes	yes	no
Malley et al	2021	J. Med. Toxicol, 17	USA	33	female	NA	duloxetine, hydroxyzine, linaclotide, dicyclomine, paracetamol	no	no	yes	yes	no
Manikinda et al	2019	Ann. Indian Acad. Neurol, 22	India	58	male	NA	escitalopram, levodopa, amantadine, rasagiline, clonazepam, carbidopa, olanzapine	yes	yes	yes	yes	no
Manos	2000	Annals of Pharmacotherapy, 34(7–8)	USA	37	male	generalized anxiety disorder	fluoxetine, buspirone	yes	yes	yes	no	no
Mantilla et al	2020	Chest, 158(4)	Netherlands	20	male	depression	amitriptyline, phenylethylamine, perphenazine	yes	yes	yes	yes	no
Margolese & Chouinard	2000	American Journal of Psychiatry, 157(6)	Canada	60	female	depression	nefazodone, trazodone, irbesartan	yes	yes	yes	no	no
Marino et al	2018	J. Med. Toxicol, 14(1)	USA	35	male	depression	paroxetine, hydroxyzine	yes	yes	yes	yes	no
Marino et al	2021	Crit. Care Med, 49(1)	USA	54	female	depression	fluoxetine, buspirone	yes	yes	yes	no	no
Marquetand et al	2020	The Journal of Critical Care Medicine, 6(2)	Germany	27	female	major depressive disorder	venlafaxine, ethanol	no	yes	yes	yes	no
Martindale & Stedeford	2003	Anaesthesia, 58(10)	UK	60	female	depression	fluoxetine, methylene blue	yes	yes	yes	yes	no
Martini et al, case 1	2015	Clinical Toxicology, 53(3)	USA	22	female	drug abuse	paroxetine, amitriptyline metaxalone, clonazepam, hydrocodone, paracetamol	yes	yes	yes	yes	no

Martini et al, case 2	2015	Clinical Toxicology, 53(3)	USA	27	male	posttraumatic stress disorder	escitalopram, metaxalone, clonazepam	yes	yes	yes	yes	no
Mas Serrano et al, case 1	2020	Journal of the Neurological Sciences, 415	Spain	66	male	bipolar disorder	duloxetine, lopinavir, hydroxychloroquine, lithium, haloperidol, ritonavir	yes	yes	yes	yes	no
Mas Serrano et al, case 2	2020	Journal of the Neurological Sciences, 415	Spain	78	male	NA	lopinavir, hydroxychloroquine, risperidone, morphine, ritonavir	yes	yes	yes	yes	no
Mason & Blackburn	1997	Annals of Pharmacotherapy, 31(2)	USA	42	female	major depression	sertraline, metaproterenol, pravastatin, chlorzoxazone, metaproterenol, tramadol, nabumetone, theophylline, naphazoline, omeprazole, terfenadine	no	yes	yes	yes	no
Mason et al	2008	Orthopedics, 31(11)	UK	58	female	severe depression	venlafaxine, linezolid	no	no	yes	no	no
Mason et al, case 1	2000	Medicine, 79(4)	USA	37	female	depression	venlafaxine, trazodone olanzapine, lorazepam	no	yes	yes	yes	no
Mason et al, case 2	2000	Medicine, 79(4)	USA	31	male	major depression	venlafaxine, olanzapine	yes	yes	yes	no	no
Mastroianni & Ravaglia	2017	Le Infezioni in Medicina, 25(3)	Italy	39	male	opioid dependence	linezolid, methadone, lamivudine, tenofovir, darunavir, ritonavir	yes	yes	yes	no	no
Matchanov & Nelson	2020	Neurodiagnostic Journal, 60(3)	USA	67	female	NA	midazolam, propofol, methadone, lidocaine, tramadol, remifentanil, ketamine, gabapentin, diazepam	no	no	yes	no	no
Mateo-Carrasco et al	2015	Pharmacotherapy, 35(6)	UK	57	female	moderate depression	phenelzine, morphine	yes	yes	yes	no	no
Mathew et al	2006	Anaesthesia, 61(6)	UK	65	male	NA	citalopram, methylene blue, fentanyl	no	yes	yes	yes	no
Mathew et al, case 1	1996	Cephalalgia, 16(5)	USA	31	female	NA	amitriptyline, metoclopramide, dihydroergotamine, prochlorperazine, propranolol	no	yes	yes	no	no
Mathew et al, case 2	1996	Cephalalgia, 16(5)	USA	43	female	anxiety disorder	paroxetine, imipramine lithium, dihydroergotamine	yes	yes	yes	no	no
Mathew et al, case 3	1996	Cephalalgia, 16(5)	USA	44	female	NA	sertraline, lithium, methysergide, sumatriptan	yes	yes	yes	no	no
Mathew et al, case 4	1996	Cephalalgia, 16(5)	USA	25	female	NA	sertraline, metoclopramide, dihydroergotamine	no	yes	yes	no	no
Mathew et al, case 5	1996	Cephalalgia, 16(5)	USA	48	female	depression	sertraline, propranolol, sumatriptan	no	yes	yes	no	no
Mathew et al, case 6	1996	Cephalalgia, 16(5)	USA	44	female	NA	sumatriptan	no	yes	yes	no	no
Matsumura et al	2024	Cureus, 16(7)	Japan	66	male	NA	fentanyl, morphine	yes	yes	yes	yes	no
Mazhar et al	2016	Case Reports in Medicine, 2016	Saudi Arabia	64	male	mild depression	fluoxetine, linezolid, olanzapine, metoclopramide	yes	yes	yes	no	no
McClean et al	2011	Irish Journal of Medical Science, 180(1)	Ireland	67	male	NA	citalopram, linezolid, metformin, rosiglitazone	yes	yes	yes	no	no
McCue & Joseph	2001	American Journal of Psychiatry, 158(12)	USA	50	male	recurrent depression	venlafaxine, trazodone, methadone	yes	yes	yes	no	no
McDaniel, case 1	2001	The Annals of Pharmacotherapy, 35(7–8)	USA	43	female	posttraumatic stress disorder	fluoxetine, mirtazapine sumatriptan, tramadol	yes	yes	yes	no	no
McDaniel, case 2	2001	The Annals of Pharmacotherapy, 35(7–8)	USA	43	female	posttraumatic stress disorder	nefazodone, valproate, sumatriptan, tramadol	yes	yes	yes	no	no

McDaniel, case 3	2001	The Annals of Pharmacotherapy, 35(7–8)	USA	47	female	treatment-resistant depression	venlafaxine, trazodone valproate, diazepam, lithium	yes	yes	yes	yes	no
McDaniel, case 4	2001	The Annals of Pharmacotherapy, 35(7–8)	USA	47	female	bipolar disorder	nefazodone, fluvoxamine olanzapine, cocaine, gabapentin	no	yes	yes	no	no
McIntosh	2000	Revue Canadienne de Psychiatrie, 45(6)	Canada	43	female	posttraumatic stress disorder	fluvoxamine	no	no	no	no	no
McIntyre et al	1997	Journal Of Forensic Sciences, 5(42)	Australia	22	male	clinical depression	moclobemide, sertraline, pimozide	no	no	yes	no	yes
Meggs & Bernard	2010	Clin. Toxicol, 48(3)	USA	45	male	depression	paroxetine, citalopram	no	no	yes	yes	yes
Meggs et al	2019	Clin. Toxicol, 57(6)	USA	25	female	depression	fluoxetine, lithium	no	no	NA	NA	yes
Mehta et al	2011	Tex. Heart Inst. J, 38(5)	USA	46	female	depression	isocarboxazid, phenethylamine, lithium	no	no	yes	yes	no
Mekler & Woggon	1997	Pharmacopsychiatry, 30(06)	Switzerland	50	female	bipolar affective disorder	clomipramine, venlafaxine, lithium	yes	yes	yes	no	no
Melani et al	2009	Epilepsy & Behavior, 14(4)	Italy	49	male	mild mental retardation	duloxetine, topiramate	yes	yes	yes	no	no
Milano et al	2017	Clinical Psychopharmacology and Neuroscience, 15(3)	Italy	70	female	major depressive disorder	venlafaxine, codeine, diazepam, rizatriptan	yes	yes	yes	no	no
Miller & Lovell	2011	The Journal of Emergency Medicine, 40(1)	USA	36	female	bipolar disorder, depression	venlafaxine, imipramine lithium, linezolid	yes	yes	yes	yes	no
Miller et al	1991	Journal of Clinical Psychopharmacology, 11(4)	USA	18	female	borderline personality disorder	tranylcypromine, fluoxetine	yes	yes	yes	yes	no
Misselbrook & Shekhar	2011	Acute Medicine, 10(4)	UK	89	female	NA	fluoxetine, clarithromycin, ramipril	yes	yes	yes	no	no
Mittino et al	2004	Clinical Neuropharmacology, 27(3)	Italy	75	female	depression	sertraline, tramadol	yes	yes	yes	no	no
Miyaoka & Kamijima	1995	International Clinical Psychopharmacology, 10(4)	Japan	46	male	depression	amitriptyline, diazepam, nitrazepam, flunitrazepam,	yes	yes	yes	no	no
Moffitt et al	2019	J. Invest. Med, 67(2)	Netherlands	34	female	major depressive disorder	venlafaxine	no	yes	yes	yes	no
Molaie	1997	Headache: The Journal of Head and Face Pain, 37(8)	USA	30	female	obsessive-compulsive disorder	fluoxetine, clomipramine	no	yes	yes	no	no
Montané et al	2009	Journal of Clinical Pharmacy and Therapeutics, 34(4)	Spain	74	male	depression	fluoxetine, reboxetine, citalopram, enalapril, diazepam, sulpiride, ciprofloxacin, oxybutynin	yes	yes	yes	no	no
Montañes-Rada et al	2005	Journal of Clinical Psychopharmacology, 25(1)	Spain	40	female	depression	venlafaxine, lorazepam	yes	yes	yes	yes	no
Monte & Waksman	2010	Journal of Clinical Psychopharmacology, 30(5)	USA	46	female	bipolar disorder, depression	citalopram, olanzapine, trimethoprim, zolpidem, sulfamethoxazole	yes	yes	yes	yes	no
Monte et al	2010	British Journal of Clinical Pharmacology, 70(6)	USA	19	male	borderline personality disorder	dextromethorphan, chlorpheniramine	yes	yes	yes	no	no
Monteiro et al	2023	Cureus, 15(10)	Portugal	42	female	depression	escitalopram, venlafaxine	yes	yes	yes	no	no
Montenij et al	2009	J. Crit. Care, 13(1)	Netherlands	25	female	depression	citalopram	yes	yes	yes	yes	no
Morales & Vermette	2005	Psychosomatics, 46(3)	USA	39	female	major depression	fluoxetine, linezolid, buspirone, cyclobenzaprine	yes	yes	yes	yes	no

Morarasu et al	2022	J. Pers. Med, 12(12)	Romania	21	male	major depression	trazodone, olanzapine, lorazepam, aripiprazole, valproate	yes	yes	yes	yes	no
Morrison & Rowe	2012	J. Clin. Pharm. Ther, 37(5)	USA	28	male	NA	buspirone, linezolid	no	yes	yes	yes	yes
Moseson & Nichols	2013	Crit. Care Med, 41(12)	USA	69	female	depression	not specified SSRI, pethidine, fentanyl	yes	yes	yes	yes	no
Mostel et al	2022	American Journal of Case Reports, 23(1)	USA	70	female	dementia	paroxetine, quetiapine	yes	yes	yes	no	no
Mueller & Korey	1998	Annals of Emergency Medicine, 32(3)	USA	20	female	NA	MDMA	no	yes	yes	yes	yes
Mulroy et al, case 1	2012	Mov. Disord, 27	Ireland	35	female	depression	phenelzine, phenylephrine hydrochloride, valproate	yes	yes	yes	yes	no
Mulroy et al, case 2	2012	Mov. Disord, 27	Ireland	57	female	depression	duloxetine, amitriptyline	no	yes	yes	yes	yes
Mulroy et al, case 3	2012	Mov. Disord, 27	Ireland	69	female	NA	venlafaxine, amitriptyline	no	no	yes	no	no
Muly et al	1993	The American Journal of Psychiatry, 150(10)	USA	36	female	major depressive episode	fluoxetine, lithium	yes	yes	yes	no	no
Munhoz	2004	Clinical Neuropharmacology, 27(5)	Brazil	62	female	depression	venlafaxine, sertraline, piracetam	yes	yes	yes	no	no
Murray et al	2021	The Journal of Emergency Medicine, 60(4)	USA	19	female	depression	bupropion	yes	yes	yes	yes	no
Mushtaq et al	2018	J. Gen. Intern. Med, 33(2)	USA	40	male	NA	duloxetine, fentanyl, ondansetron, tramadol	yes	yes	yes	yes	no
Muzyk et al	2010	Psychosomatics, 51(5)	USA	55	male	attention deficit hyperactivity disorder	paroxetine	yes	yes	yes	yes	yes
Nagamine	2022	Aust. New Zealand J. Psychiatry, 56(7)	Japan	74	male	depression	fluvoxamine, mirtazapine	yes	yes	yes	no	no
Nagarsheth et al	2009	Crit. Care Med, 37(12)	USA	39	male	bipolar disorder	citalopram, metoclopramide	no	no	yes	yes	no
Nagy et al	2023	Clinical Case Reports, 11(7)	USA	61	female	NA	fluoxetine, bupropion methylene blue, pethidine	yes	yes	yes	yes	no
Naik & Rincon-Aznar	2015	The Journal of Critical Care Medicine, 1(4)	UK	58	male	severe depression	venlafaxine, methylene blue, fentanyl, morphine, ondansetron	no	yes	yes	yes	no
Nair & Chandy	2015	Indian Journal of Anaesthesia, 59(4)	India	43	male	NA	fentanyl, tramadol	no	no	yes	yes	no
Nakayama et al, case 1	2014	Neuropsychiatr Dis Treat, 10	Japan	64	female	psychotic disorder	paroxetine, perospirone	yes	yes	yes	no	no
Nakayama et al, case 2	2014	Neuropsychiatr Dis Treat, 10	Japan	81	female	dementia with psychotic symptoms	paroxetine, perospirone	yes	yes	yes	no	no
Nardell	2015	J. Gen. Intern. Med, 30	USA	25	male	NA	fluoxetine, methylene blue, haloperidol	yes	yes	yes	yes	no
Nash et al	2021	Toxicol	Australia	31	male	depression	escitalopram	yes	yes	yes	yes	no
Nasser et al	2023	Cureus, 15(8)	USA	40	female	bipolar ii disorder	buspirone, clonazepam, kratom, lamotrigine, quetiapine	yes	yes	yes	no	no
Navarro et al	2006	General Hospital Psychiatry, 28(1)	USA	22	male	bipolar i disorder	fluoxetine, lithium, clonazepam, dextromethorphan	yes	yes	yes	no	no
Navarroza et al, case 1	2018	Int. J. Rheum. Dis, 21	Philippines	36	female	NA	vancomycin, piperacillin, tramadol, linezolid, tazobactam	yes	yes	yes	no	no

Navarroza et al, case 2	2018	Int. J. Rheum. Dis, 21	Philippines	36	female	NA	linezolid, tramadol, isoniazid, rifampicin, ethambutol, pyrazinamide, amoxicillin, clindamycin, cefixime, vancomycin, clavulanate	yes	yes	yes	no	no
Nayyar	2009	Indian Journal of Psychiatry, 51(1)	USA	55	male	major depressive disorder	sertraline, trazodone, tramadol	yes	yes	yes	no	no
Necpál & Skorvanek	2017	Journal of the Neurological Sciences, 372	Slovakia	28	female	posttraumatic stress disorder	venlafaxine, ethanol	no	yes	yes	yes	no
Nefcy et al	2013	Clinical Toxicology, 51(7)	USA	19	male	NA	NBOME compounds	yes	yes	yes	yes	no
Nemet et al	2023	Cureus, 15(3)	Serbia	71	female	depression	duloxetine, levodopa, benserazide, entacapone, ropinirole, rasagiline	yes	yes	yes	yes	yes
Neuvonen et al, case 1	1993	The Lancet, 342(8884)	Finland	19	female	NA	moclobemide, clomipramine	no	yes	yes	yes	yes
Neuvonen et al, case 2	1993	The Lancet, 342(8884)	Finland	23	male	NA	moclobemide, clomipramine	no	no	yes	yes	yes
Newey et al, case 1	2011	Psychosomatics, 52(3)	USA	33	female	major depressive disorder	venlafaxine, cyclosporine	yes	yes	yes	no	no
Newey et al, case 2	2011	Psychosomatics, 52(3)	USA	61	female	major depressive disorder	venlafaxine, tacrolimus	no	yes	yes	no	no
Ng et al, case 1	2008	Canadian Journal of Anesthesia, 55(1)	New Zealand	58	female	obsessive-compulsive disorder	paroxetine, omeprazole, simvastatin, methylene blue, midazolam, fentanyl, propofol, rocuronium	yes	yes	yes	yes	no
Ng et al, case 2	2008	Canadian Journal of Anesthesia, 55(1)	New Zealand	58	female	obsessive-compulsive disorder	paroxetine, methylene blue, fentanyl	no	yes	yes	no	no
Nguyen & Sin	2015	The Consultant Pharmacist, 30(8)	USA	86	male	depression	citalopram, trazodone, linezolid	no	yes	yes	no	no
Nguyen et al	2023	Cureus, 15(11)	USA	79	male	posttraumatic stress disorder	sertraline, duloxetine, atomoxetine	no	yes	no	no	no
Nicolaou & Lee	2016	Canadian Journal of Anesthesia, 63(7)	Canada	50	female	depression	paroxetine, methylene blue, hydromorphone, ondansetron	yes	yes	yes	yes	no
Nierenberg & Semprebon, case 1	1993	Clinical Pharmacology and Therapeutics, 53(1)	UK	41	female	depression	clomipramine, phenelzine haloperidol, benztropine, clonazepam	yes	yes	yes	yes	no
Nierenberg & Semprebon, case 2	1993	Clinical Pharmacology and Therapeutics, 53(1)	UK	28	female	depression	phenelzine, dextromethorphan	yes	yes	yes	yes	no
Nijhawan et al	1996	Critical Care Medicine, 24(6)	USA	48	male	alcohol use disorder	clomipramine, fluoxetine buspirone, ethanol	yes	yes	yes	yes	no
Nikolaou et al	2015	Annals of General Psychiatry, 14(1)	Greece	33	male	autistic spectrum disorder	clomipramine, mirtazapine, risperidone	yes	yes	yes	no	no
Nishi et al	2014	J. Am. Coll. Cardiol, 63(12)	Japan	57	male	schizophrenia	fluvoxamine, duloxetine	no	no	yes	no	no
Nisijima	2015	Asian Journal of Psychiatry, 18	Japan	62	male	major depressive disorder	imipramine, lithium, metoclopramide	yes	yes	yes	yes	no
Nisijima et al	1996	International Clinical Psychopharmacology, 11(4)	Japan	59	male	bipolar disorder	trazodone, amitriptyline lithium, levomepromazine, nitrazepam	yes	yes	yes	no	no
Njoku et al	2022	Journal of the Academy of Consultation-Liaison Psychiatry, 63	USA	60	male	major depressive disorder	duloxetine, fluoxetine, mirtazapine, methylene blue, gabapentin	yes	yes	yes	yes	NA

Noe et al	2024	Journal of Clinical Psychopharmacology, 44(3)	USA	42	female	major depressive disorder	nortriptyline, duloxetine buspirone, metoclopramide	yes	yes	yes	no	no
Nordstrom et al	2016	Journal of Emergency Medicine, 50(1)	USA	72	female	NA	citalopram, tramadol, methylphenidate	yes	yes	NA	NA	NA
Nunes & Botas	2020	The Primary Care Companion For CNS Disorders, 22(4)	Portugal	47	female	bipolar i disorder	escitalopram, buspirone, lithium, diazepam	yes	no	yes	no	no
O'Brien et al	2021	Antimicrob. Agents Chemother, 65(1)	USA	35	female	depression	mirtazapine, isoniazid, pyridoxine	no	no	yes	no	no
Öhman & Spigset	1993	Pharmacopsychiatry, 26(06)	Sweden	53	female	bipolar disorder	fluvoxamine, lithium	no	no	yes	no	no
Ohta & Sano	2022	Cureus, 14(2)	Japan	65	female	depression	paroxetine, trazodone levodopa, caffeine, carbidopa	yes	yes	yes	no	no
Okamoto et al	2012	Case Reports in Psychiatry, 2012	Japan	67	female	major depressive disorder with psychotic features	paroxetine	yes	yes	yes	no	no
Okamoto et al, case 1	2010	J. Clin. Psychopharmacol, 30(3)	Japan	67	male	recurrent depression	paroxetine	yes	yes	no	no	no
Okamoto et al, case 2	2010	J. Clin. Psychopharmacol, 30(3)	Japan	66	male	major depression	paroxetine, clomipramine	yes	yes	no	no	no
Oliver et al, case 1	2002	Human & Experimental Toxicology, 21(8)	UK	24	female	NA	venlafaxine, ethanol	no	no	yes	yes	no
Oliver et al, case 2	2002	Human & Experimental Toxicology, 21(8)	UK	21	female	depression	venlafaxine, citalopram codeine, paracetamol	no	no	yes	yes	no
Ölmeztoprak et al	2017	Psychiatry and Clinical Psychopharmacology, 27(sup1)	Turkey	62	female	psychotic depression	fluoxetine	no	yes	yes	no	no
Ong & Vasanwala	2018	The Primary Care Companion For CNS Disorders, 20(3)	Singapore	69	female	late-onset psychosis	vortioxetine, propranolol	no	yes	yes	no	no
Ooi	1991	Anaesthesia, 46(6)	UK	72	female	depression	fluoxetine, tranylcypromine, trifluoperazine	no	yes	yes	yes	no
Oroian et al	2019	Chest, 156(4)	USA	20	female	bipolar i disorder	lamotrigine	no	no	yes	yes	no
Ortés-Gómez et al	2016	Eur. Geriatr. Med, 7	Spain	84	female	depression	paroxetine, fentanyl, quetiapine	yes	yes	yes	yes	yes
Ott et al	2019	Ther. Adv. Psychopharmacol, 9	Sweden	73	female	chronic depression	escitalopram, venlafaxine furosemide, aspirin, erythromycin	yes	yes	yes	yes	yes
Otte et al	2003	European Psychiatry, 18(5)	Netherlands	60	male	major depressive episode	imipramine, tranylcypromine, lithium	yes	yes	yes	yes	yes
Ozdemir et al	2008	Progress in Neuro-Psychopharmacology and Biological Psychiatry, 32(3)	Turkey	66	male	major depression	sertraline	yes	yes	yes	no	no
Packer & Berman	2007	The American Journal of Psychiatry, 164(2)	USA	30	female	major depressive disorder	venlafaxine, linezolid	no	yes	no	no	no
Palekar & Eisman	2013	The Journal of Neuropsychiatry and	USA	30	female	NA	sertraline, ziprasidone	yes	yes	yes	no	no

		Clinical Neurosciences, 25(2)										
Palimaru et al	2021	Psychiatry, 2021	France	38	female	nonverbal autism	sertraline, valproate, diazepam, melatonin, sodium alginate/sodium bicarbonate, lansoprazole, phloroglucinol	no	yes	yes	yes	no
Pan & Shen	2003	Annals of Pharmacotherapy, 37(2)	China	29	female	major depressive disorder	venlafaxine, imipramine	yes	yes	no	no	no
Pandya et al	2020	Case Reports in Psychiatry, 2020(1)	USA	74	male	depression	venlafaxine, trazodone, valproate, quetiapine	yes	yes	yes	yes	no
Papuchis et al	2018	PM R, 10(9)	USA	57	NA	depression	escitalopram, tramadol	no	no	yes	no	no
Park & Jung	2010	Progress in Neuro-Psychopharmacology and Biological Psychiatry, 34(4)	South Korea	18	male	major depression	paroxetine, methylphenidate	yes	yes	yes	no	no
Parker et al	2001	The Canadian Journal of Psychiatry, 46(1)	Australia	40	male	anxiety disorder	st john's wort, clonazepam	no	yes	no	no	no
Paruchuri et al	2006	American Journal of Therapeutics, 13(6)	USA	80	female	major depressive disorder	paroxetine	yes	yes	yes	no	no
Passmore et al	2004	The Canadian Journal of Psychiatry, 49(1)	Canada	74	female	major depressive disorder	trazodone, clonazepam	yes	yes	yes	no	no
Patel & Marzella	2017	American Journal of Case Reports, 18	USA	28	male	bipolar disorder	sertraline, bupropion, bupropion, valproate, prazosin, hydroxyzine, sildenafil, cyclobenzaprine	yes	yes	yes	yes	no
Patil & Ghanate	2019	Indian J. Psychiatry, 61(9)	India	45	female	bipolar affective disorder	escitalopram, fluoxetine, valproate, olanzapine	yes	yes	yes	no	no
Paul et al	2017	J. Gen. Intern. Med, 32(2)	USA	47	female	depression	vortioxetine, duloxetine, sertraline	no	no	yes	no	no
Peacock & Wright	2011	Age and Ageing, 40(4)	UK	78	female	NA	citalopram, tramadol	no	yes	yes	no	no
Pearce et al	2009	The Consultant Pharmacist, 24(1)	USA	79	female	major depressive disorder	paroxetine, mirtazapine, donepezil	no	yes	yes	no	no
Perry	2000	Postgraduate Medical Journal, 76(894)	UK	75	male	depression	venlafaxine	no	no	yes	no	no
Pettit et al	2016	J. Clin. Pharm. Ther, 41(1)	USA	89	female	NA	linezolid, carbidopa, levodopa	yes	yes	yes	no	no
Pfisterer & Yonclas	2011	PM R, 3(10)	USA	42	female	NA	nortriptyline, tramadol	no	yes	no	no	no
Phillips & Holden	2020	Critical Care Medicine, 48(1)	USA	25	male	depression	escitalopram, topiramate, lamotrigine, THC	no	yes	yes	yes	no
Phuditshinnapatra et al	2019	Clin. Toxicol, 57(12)	Thailand	21	female	major depressive disorder	fluoxetine	yes	yes	yes	no	no
Picco et al	2019	Palliat. Med. Prac, 13(3)	Poland	56	female	depression disorder	paroxetine, mirtazapine tramadol, paracetamol	yes	yes	yes	no	no
Pinel-Ríos et al	2016	Actas Españolas de Psiquiatría, 44(5)	Spain	54	male	moderate depressive episode	venlafaxine, clomipramine levomepromazine, clorazepate, lormetazepam, enalapril	yes	yes	yes	yes	no
Poeschla et al	2011	General Hospital Psychiatry, 33(3)	USA	79	male	severe major depression with somatic delusions	venlafaxine, mirtazapine donepezil, quetiapine	yes	yes	yes	no	no

Power et al	1995	Anaesthesia and Intensive Care, 23(4)	Australia	40	female	suicidal attempt	moclobemide, clomipramine, fluoxetine, clonazepam	no	yes	yes	yes	yes
Prakash et al	2016	Neurol. India, 64(6)	India	28	male	depression	fluoxetine, citalopram tramadol, paracetamol	yes	yes	yes	no	no
Prakash et al	2019	Neurol India, 67(1)	India	19	male	seizures	valproate, carbamazepine	yes	yes	yes	no	no
Prakash et al	2020	BMJ Case Rep, 13(12)	India	37	male	major depression	amitriptyline, paroxetine	yes	yes	yes	no	no
Prakash et al	2021	BMJ Case Rep, 14(8)	India	21	male	bipolar disorder	escitalopram, tramadol, risperidone, dextromethorphan, azithromycin, paracetamol	yes	yes	yes	yes	no
Prakash et al	2024	BMJ Case Rep, 17(4)	India	35	male	anxiety disorder	sertraline, amitriptyline tramadol, paracetamol	yes	yes	yes	no	no
Prakash et al , case 1	2014	Cephalalgia, 34(2)	India	22	male	NA	tramadol, paracetamol	yes	no	no	no	no
Prakash et al, case 1	2014	Gen. Hosp. Psychiatry, 36(4)	India	46	male	NA	sertraline, tramadol	no	yes	no	no	no
Prakash et al, case 1	2016	Neurol. Sci., 37(9)	India	19	male	NA	dextromethorphan, tramadol, ondansetron	yes	yes	yes	no	no
Prakash et al, case 1	2016	Indian J Crit Care Med, 20(2)	India	43	male	NA	paroxetine, tramadol, ondansetron, paracetamol	yes	yes	yes	no	no
Prakash et al, case 1	2017	BMJ Case Rep	India	49	male	NA	amitriptyline, lithium	yes	yes	yes	no	no
Prakash et al, case 1	2021	Ann Indian Acad Neurol, 24(3)	India	47	male	NA	amoxicillin, dextromethorphan, paracetamol, tramadol	yes	yes	yes	no	no
Prakash et al, case 1	2023	BMJ Case Rep, 17(1)	India	30	female	NA	tramadol, sumatriptan, valproate, topiramate	yes	yes	no	no	no
Prakash et al, case 2	2013	Cephalalgia, 34(2)	India	46	male	NA	fluoxetine, tramadol, ondansetron, paracetamol	yes	yes	no	no	no
Prakash et al, case 2	2014	Gen. Hosp. Psychiatry, 36(4)	India	54	female	NA	fluoxetine, tramadol	no	no	no	no	no
Prakash et al, case 2	2016	Neurol. Sci., 37(9)	India	32	male	depression	fluoxetine, tramadol	yes	yes	yes	no	no
Prakash et al, case 2	2016	Indian J Crit Care Med, 20(2)	India	62	male	depression	sertraline, clonazepam	yes	yes	yes	no	no
Prakash et al, case 2	2017	BMJ Case Rep	India	37	male	NA	amitriptyline, valproate	yes	yes	yes	no	no
Prakash et al, case 2	2021	Ann Indian Acad Neurol, 24(3)	India	34	male	bipolar disorder	fluoxetine, amitriptyline	yes	yes	yes	no	no
Prakash et al, case 2	2023	BMJ Case Rep, 17(1)	India	35	female	NA	amitriptyline, sumatriptan, rizatriptan, tramadol, ergotamine	yes	yes	no	no	no
Prakash et al, case 3	2013	Cephalalgia, 34(2)	India	36	female	NA	fluoxetine, tramadol, paracetamol	yes	yes	no	no	no
Prakash et al, case 3	2014	Gen. Hosp. Psychiatry, 36(4)	India	58	male	NA	sertraline, tramadol	no	yes	no	no	no
Prakash et al, case 3	2016	Neurol. Sci., 37(9)	India	36	male	NA	amitriptyline, valproate	yes	yes	yes	no	no
Prakash et al, case 4	2013	Cephalalgia, 34(2)	India	34	male	depression	paroxetine, clonazepam	yes	yes	no	no	no
Prakash et al, case 4	2014	Gen. Hosp. Psychiatry, 36(4)	India	65	male	NA	fluoxetine, tramadol	no	yes	no	no	no
Prakash et al, case 5	2014	Gen. Hosp. Psychiatry, 36(4)	India	48	male	NA	paroxetine	no	yes	no	no	no

Précourt et al	2005	The Annals of Pharmacotherapy, 39(1)	Canada	53	male	NA	venlafaxine, quetiapine	yes	yes	yes	yes	no
Price et al	1986	The Journal of Clinical Pharmacology, 26(1)	USA	63	male	major depression with psychotic features	tranylcypromine, tryptophan, lithium	no	yes	yes	no	no
Primeau et al	2012	The Journal of Neuropsychiatry and Clinical Neurosciences, 24(1)	USA	64	male	NA	citalopram, aripiprazole	yes	yes	yes	no	no
Prior et al	2002	Medical Journal of Australia, 176(5)	USA	32	male	attention deficit hyperactivity disorder	venlafaxine, dexamphetamine	yes	yes	no	no	no
Proudfoot & Gormley	2013	BMJ Case Reports, 2013	UK	NA	male	mild depression	fluoxetine, codeine	yes	yes	yes	yes	no
Quantrille & Kleinman	2021	J. Invest. Med, 69(2)	USA	31	female	depression	escitalopram, buspirone	yes	yes	yes	no	no
Rachid et al	2006	The World Journal of Biological Psychiatry, 7(4)	Switzerland	39	female	chronic major depression	paroxetine, amitriptyline, opipramol prazepam, methadone	yes	yes	yes	no	no
Ragsdell et al	2021	The Primary Care Companion For CNS Disorders, 23(4)	USA	37	female	major depression	fluoxetine, dexbrompheniramine, metoclopramide, uncaria tomentosa, tramadol, phenylephrine	yes	yes	yes	no	no
Rai et al	2014	Chest, 146(4)	USA	41	male	bipolar disorder	desvenlafaxine, MDMA, risperidone	yes	yes	yes	yes	no
Rajapakse et al	2010	Journal of Clinical Psychopharmacology, 30(5)	Sri Lanka	43	male	depression	venlafaxine, levodopa, amantadine, trihexyphenidyl, ropinirole, carbidopa	yes	yes	yes	yes	no
Ramachandran et al	2018	Baylor University Medical Center Proceedings, 31(1)	USA	52	female	bipolar disorder, depressive	fluoxetine, trazodone aripiprazole, cyclobenzaprine	yes	yes	yes	no	no
Rang et al	2008	Canadian Journal of Anesthesia, 55(8)	UK	60	female	depression	paroxetine, fentanyl	yes	yes	yes	yes	no
Rao	1997	International Journal of Geriatric Psychiatry, 12(1)	UK	73	male	mixed anxiety/depressive disorder	trazodone, temazepam, chlorpromazine	yes	yes	yes	no	yes
Rao	2017	J. Am. Geriatr. Soc, 65	USA	65	female	depression	venlafaxine, trazodone, ondansetron	yes	no	yes	yes	NA
Rastogi et al, case 1	2011	Anesthesiology, 115(6)	USA	58	male	depression	citalopram, mirtazapine fentanyl, oxycodone, celecoxib, zolpidem, paracetamol	no	yes	no	no	no
Rastogi et al, case 2	2011	Anesthesiology, 115(6)	USA	45	male	depression	duloxetine, desipramine, methadone	yes	yes	no	no	no
Reeves & Bullen	1995	Psychosomatics, 36(2)	USA	29	female	first depressive episode	trazodone, paroxetine	yes	yes	yes	no	no
Reeves et al	2002	Annals of Pharmacotherapy, 36(3)	USA	24	female	mental retardation	fluvoxamine, risperidone	yes	yes	yes	yes	no
Reinert et al	2022	Critical Care Medicine, 50(1)	USA	63	female	depression	sertraline, lisinopril	no	no	yes	yes	no
Rella & Hoffman	1998	Journal of Toxicology: Clinical Toxicology, 36(3)	USA	39	female	anxiety disorder	paroxetine, clonazepam	no	yes	no	no	no
Ren et al	2012	Parkinsonism Relat. Disord, 18	China	50	female	depression	citalopram, selegiline, levodopa	no	no	no	no	no
Rim & Gitlin	2010	Journal of Clinical Psychopharmacology, 30(4)	USA	41	female	chronic major depression with atypical features	bupropion, duloxetine, tranylcypromine, ziprasidone	yes	yes	yes	yes	no

Rosebraugh et al	2001	The Journal of Clinical Pharmacology, 41(2)	USA	34	male	NA	sertraline, oxycodone, methylprednisolone, cyclosporin, fluconazole	no	no	yes	no	no
Roxanas & Machadot	1998	Medical Journal of Australia, 168(10)	Australia	34	male	depression	venlafaxine, moclobemide ethanol, temazepam	yes	yes	yes	yes	no
Roy & Massie	2011	J. Gen. Intern. Med, 26	USA	39	female	depression	citalopram, amitriptyline, methadone, trimethoprim, buspirone, quetiapine, levothyroxine, sulfamethoxazole, zonisamide, gabapentin, metformin, pravastatin, meclizine, promethazine, prochlorperazine	yes	yes	yes	yes	no
Rubin et al	2018	Cureus, 10(11)	USA	64	female	anxiety disorder	citalopram, paroxetine oxycodone, clonazepam, lamotrigine, gabapentin	yes	yes	yes	no	no
Rudisill et al	2011	Community Oncol, 8(1)	USA	52	female	mild depression	duloxetine, pethidine, midazolam, promethazine	yes	yes	no	no	no
Ruiz	1994	Annals of Emergency Medicine, 24(5)	USA	48	male	depression	tranylcypromine, fluoxetine	yes	yes	yes	yes	no
Ruiz de Villa et al	2021	Cureus, 13(11)	USA	79	female	major depressive episode	citalopram, trazodone tramadol, oxycodone, gabapentin	no	yes	yes	no	no
Rutkowski et al	2022	Journal of the Academy of Consultation-Liaison Psychiatry, 63	Israel	26	female	depression	venlafaxine, citalopram, nortriptyline	no	no	no	no	no
Samara & Warner	2017	BMJ Case Reports, 2017	USA	68	female	depression	paroxetine, trazodone omeprazole, dexamphetamine, hydromorphone, risperidone, zendocrine	yes	yes	yes	yes	no
Samartzis et al	2013	Case Reports in Psychiatry, 2013	Cyprus	68	female	major depression	amitriptyline, fentanyl, linezolid	yes	yes	yes	yes	no
Samavedam et al	2016	Crit. Care Med, 44(12)	USA	19	male	depression	bupropion	no	no	yes	yes	no
Sanyal et al	2010	Indian Journal of Pharmacology, 42(6)	India	73	female	depression	escitalopram, selegiline	yes	yes	yes	yes	no
Sasaki et al	2013	Journal of Cardiology Cases, 7(1)	Japan	65	female	depression	maprotiline, dextromethorphan	yes	yes	yes	yes	no
Sato et al	2004	Mayo Clinic Proceedings, 79(11)	Japan	39	male	panic disorder	paroxetine, digoxin, ticlopidine, disopyramide phosphate, omeprazole	yes	yes	yes	no	no
Sato et al	2015	Neuropsychiatric Disease and Treatment, 11	Japan	78	male	major depressive disorder	escitalopram, risperidone	no	yes	yes	no	no
Satoh et al	2006	Fukushima Journal Of Medical Science, 52(1)	Japan	18	male	depressive episode	fluvoxamine, clonazepam, brotizolam, etizolam	yes	yes	yes	no	no
Schep et al	2013	Clinical Toxicology, 51(4)	New Zealand	26	female	suicide attempt	citalopram, tramadol	yes	yes	yes	no	NA
Schindzielorz, case 1	2022	Psychiatry, 2022	USA	18	female	unspecified anxiety disorder	fluoxetine	yes	yes	yes	no	no
Schindzielorz, case 1	2022	Psychiatry, 2022	USA	42	male	bipolar i disorder	lithium, olanzapine	yes	yes	yes	yes	no
Schnitzer et al	2015	PM R, 7(9)	USA	56	female	NA	milnacipran, tramadol, pregabalin	no	no	no	no	no
Schuch et al	2016	Military Medicine, 181(9)	USA	29	male	posttraumatic stress disorder	citalopram, mirtazapine morphine, fentanyl, morphine, ondansetron	no	yes	yes	yes	no

Schult et al	2019	The American Journal of Emergency Medicine, 37(10)	USA	25	female	depression	citalopram, fentanyl	yes	yes	yes	yes	no
Schumacher et al	2017	Journal of Thoracic and Cardiovascular Surgery, 154(3)	USA	60	male	depression	citalopram, methylene blue, fentanyl	no	yes	yes	yes	no
Schwartz et al	2008	Clinical Toxicology, 46(8)	USA	20	male	depression	escitalopram, dextromethorphan, benztropine, aripiprazole	yes	yes	yes	yes	no
Schwiebert et al	2009	Anaesthesia, 64(8)	UK	NA	female	NA	paroxetine, methylene blue	yes	yes	yes	NA	NA
Scoccimarro et al	2018	Journal of Medical Toxicology, 14(1)	USA	36	male	NA	loperamide	no	yes	yes	yes	no
Seet & Rotella	2021	Emergency Medicine Australasia, 33(5)	Australia	29	male	anxiety disorder	sertraline, lavandula angustifolia, budesonide,formoterol	yes	yes	yes	no	no
Sener et al	2005	Indian J. Crit. Care Med, 9(3)	Turkey	34	female	NA	moclobemide	yes	yes	yes	yes	yes
Sethi et al	2012	The Primary Care Companion For CNS Disorders, 14(6)	USA	46	male	depression	sertraline, dextromethorphan	yes	yes	yes	yes	no
Shah & Jain	2016	Indian Journal of Pharmacology, 48(1)	India	58	male	obsessive-compulsive disorder	fluoxetine, sertraline, fluvoxamine, linezolid, fentanyl	yes	yes	yes	yes	no
Shahani	2012	The Journal of Neuropsychiatry and Clinical Neurosciences, 24(4)	USA	62	male	major depressive episode	citalopram, bupropion, tramadol	yes	yes	yes	no	no
Shahani	2012	The Journal of Neuropsychiatry and Clinical Neurosciences, 24(3)	USA	42	female	major depressive disorder	venlafaxine, lithium	yes	yes	yes	yes	no
Shahbazi & Shojaei, case 1	2025	Current Drug Safety, 20(2)	Iran	49	male	NA	linezolid, fentanyl, fentanyl, tacrolimus, mycophenolate mofetil, prednisolone, amlodipine, insulin, cefepime, methylprednisolone, remdesivir	yes	yes	yes	yes	no
Shahbazi & Shojaei, case 2	2025	Current Drug Safety, 20(2)	Iran	52	female	NA	doxepin, clomipramine, linezolid, amlodipine, amlodipine, valsartan, clonazepam, acetylsalicylic acid, insulin	no	no	yes	yes	no
Shaikh et al	2011	The Annals of The Royal College of Surgeons of England, 93(8)	UK	77	male	mild depression	fluoxetine, linezolid	yes	yes	yes	yes	no
Shakoor et al	2014	American Journal of Case Reports, 15	USA	44	female	major depression	citalopram, tramadol	yes	yes	yes	no	no
Shanmugam et al	2008	Interactive CardioVascular and Thoracic Surgery, 7(4)	Canada	49	female	depression	paroxetine, fentanyl, clonazepam, quetiapine	yes	yes	yes	yes	no
Sharma	2016	Prim. Care Companion J. Clin. Psych, 18(6)	India	63	female	bipolar ii disorder	tramadol, lithium, quetiapine	no	no	yes	no	no
Sharma et al	2023	Cureus, 15(10)	USA	60	male	depression	venlafaxine, cobicistat, fentanyl, fluconazole, oxycodone	yes	yes	yes	yes	no
Shergill et al	2022	Chest, 162(4)	USA	18	male	depression	paroxetine	yes	yes	yes	yes	no

Shioda et al	2004	Human Psychopharmacology: Clinical and Experimental, 19(5)	Japan	30	female	major depression	paroxetine, caffeine	yes	yes	yes	yes	no
Shprecher et al	2013	BMJ Case Reports, 2013	USA	55	male	NA	cyclobenzaprine, oxycodone	no	no	no	no	no
Shuster	2008	Hosp. Pharm, 43(4)	USA	78	male	NA	amantadine	yes	yes	yes	no	no
Siddiqui et al	2023	A&A Practice, 17(11)	USA	21	female	intellectual disability	fluoxetine, clozapine, valproate, fentanyl	yes	yes	yes	yes	no
Sim & Sun	2016	New England Journal of Medicine, 375(18)	China	46	female	NA	venlafaxine, alprazolam, estazolam	yes	yes	yes	yes	yes
Singh	2019	J. Basic Clin. Physiol. Pharmacol, 30(2)	India	75	female	NA	tramadol, rabeprazole, ondansetron	no	no	yes	no	no
Singh & Johnson	2024	The Journal of Clinical Psychiatry, 85(2)	USA	68	male	major depressive disorder	fluoxetine, bupropion, dextromethorphan	yes	yes	yes	no	no
Singh et al	2019	Chest, 156(4)	USA	28	male	NA	LSD	yes	yes	yes	yes	no
Skop et al	1994	The American Journal of Emergency Medicine, 12(6)	USA	51	male	posttraumatic stress disorder	paroxetine, doxylamine, diazepam, diltiazem, nitroglycerin	yes	yes	yes	yes	no
Smilkstein et al	1987	Journal of Toxicology: Clinical Toxicology, 25(1–2)	USA	50	male	depression	phenelzine, MDMA, cimetidine, diazepam	yes	yes	yes	yes	no
Smischney et al	2018	American Journal of Case Reports, 19	USA	70	male	NA	venlafaxine, fentanyl, pethidine	yes	yes	yes	no	no
Smith & Wenegrat	2000	The Journal of Clinical Psychiatry, 61(2)	USA	50	male	major depressive disorder	fluoxetine, nefazodone, alpha interferon	yes	yes	yes	no	no
Smith et al	2014	Chest, 146(4)	USA	42	female	major depressive disorder	citalopram, temazepam, clonazepam	yes	yes	yes	yes	no
Smith et al	2015	Journal of Pharmacy Practice, 28(2)	USA	59	female	borderline personality disorder	venlafaxine, trazodone, methylene blue	yes	yes	yes	yes	no
Smith et al, case 1	2010	Clin. Toxicol, 48(6)	USA	33	female	depression	sertraline, methylene blue	yes	yes	yes	no	no
Smith et al, case 2	2010	Clin. Toxicol, 48(6)	USA	42	female	depression	venlafaxine, methylene blue	yes	yes	yes	yes	no
Snyder et al	2017	Cancer Control, 24(5)	USA	58	female	generalized anxiety disorder	paroxetine, methylene blue, ondansetron, metoclopramide, oxycodone	yes	yes	yes	yes	no
Sobanski et al	1997	Pharmacopsychiatry, 30(03)	Germany	59	female	bipolar disorder	paroxetine, lithium	no	yes	yes	no	no
Solomons et al	2005	American Journal of Psychiatry, 162(6)	Canada	39	female	posttraumatic stress disorder	fluvoxamine, clonazepam	no	no	no	no	no
Song	2013	Pain Physician, 16(5)	South Korea	75	female	NA	oxycodone, pregabalin	yes	yes	yes	no	no
Sorscher	2002	Journal of Psychopharmacology, 16(2)	USA	49	female	NA	sertraline, dolasetron, doxorubicin, cyclophosphamide	no	no	no	no	no
Spigset & Adielsson	1997	International Clinical Psychopharmacology, 12(1)	Sweden	69	female	depression	citalopram, buspirone	yes	yes	yes	no	no
Spigset et al	1993	BMJ, 306(6872)	Sweden	76	female	depression	clomipramine, moclobemide levodopa, bromocriptine, dextropropoxyphene, triazolam, benserazide	no	yes	yes	no	no

Srinivasa et al	2019	Crit. Care Med, 47(1)	USA	36	female	posttraumatic stress disorder	venlafaxine, zolpidem	no	yes	yes	yes	no
Srisuma et al	2015	Clinical Toxicology, 53(8)	Thailand	62	male	obsessive-compulsive disorder	sertraline, clozapine, clonazepam	yes	yes	yes	no	no
Stanford & Stanford	1999	Journal of Psychopharmacology, 13(3)	UK	49	female	depression	paroxetine, indomethacin, ondansetron, propofol, morphine, diclofenac	yes	yes	yes	yes	no
Steinberg & Morin	2007	American Journal of Health-System Pharmacy, 64(1)	USA	23	male	bipolar disorder, depression	fluoxetine, linezolid, methadone, voriconazole, quetiapine	no	no	yes	no	no
Stevenson et al	2013	JAMA Neurology, 70(8)	USA	47	female	schizophrenia	citalopram, clozapine, ondansetron	no	yes	yes	yes	no
Stinnett & Neill	2009	Crit. Care Med, 37(12)	USA	41	male	NA	venlafaxine, sertraline morphine, linezolid, oxycodone	yes	yes	yes	yes	no
Stream & Jang	2018	J. Hosp. Med, 13(4)	USA	23	female	generalized anxiety disorder	duloxetine, fluoxetine	yes	yes	yes	no	no
Strouse et al	2006	Journal of Clinical Psychopharmacology, 26(6)	USA	55	female	major depression	duloxetine, fentanyl, gabapentin, linezolid	yes	yes	yes	no	yes
Sugaya et al	2024	Medicine, 103(15)	Japan	30	male	depression	mirtazapine, venlafaxine, lurasidone	yes	yes	yes	no	no
Sundar et al	2023	Eur. J. Mol. Clin. Med, 10(1)	India	47	female	NA	escitalopram	yes	yes	yes	no	no
Suphanklang et al	2015	Journal of the Medical Association of Thailand, 98(12)	Thailand	77	male	depression	escitalopram, rasagiline, levodopa, clonazepam, carbidopa, entacapone, pramipexole	no	yes	yes	yes	no
Suratos et al	2020	Neurodegenerative Disease Management, 10(4)	Philippines	NA	female	depression	escitalopram, rasagiline, carbidopa, levodopa, carbidopa	yes	yes	yes	yes	no
Surmaitis et al	2016	Am. J. Emerg. Med, 34(2)	USA	65	male	depression	venlafaxine, metaxalone, quetiapine	yes	yes	yes	yes	no
Suzuki & Otani	2019	Clinical Neuropharmacology, 42(3)	Japan	26	male	major depressive disorder	escitalopram, clomipramine ethanol, flunitrazepam	yes	yes	yes	no	no
Szakaly & Strauss	2008	Journal of Oral and Maxillofacial Surgery, 66(9)	USA	43	male	depression	bupropion, clindamycin	yes	yes	yes	yes	no
Szólics et al	2012	Journal of Neuroradiology, 39(4)	United Arab Emirates	28	male	depression	fluoxetine	no	yes	yes	yes	no
Tahir	2004	Journal of the American Medical Directors Association, 5(2)	USA	85	female	major depressive disorder	citalopram, linezolid	yes	yes	yes	no	no
Takata et al	2019	JA Clinical Reports, 5(1)	Japan	31	female	depression	duloxetine, mirtazapine, fentanyl	yes	yes	yes	yes	no
Talarico et al, case 1	2011	Neurological Sciences, 32(3)	Italy	75	male	Alzheimer's dementia	citalopram, cimetidine, donepezil, quetiapine, ticlopidine	yes	yes	yes	no	no
Talarico et al, case 2	2011	Neurological Sciences, 32(3)	Italy	65	female	mild cognitive impairment	citalopram, topiramate	yes	yes	yes	no	no
Tancer & Uduman	2021	Gen. Intern. Med, 36	USA	33	male	schizoaffective disorder	escitalopram	yes	yes	yes	yes	no
Terao & Hikichi	2007	Progress in Neuro-Psychopharmacology and	Japan	65	female	major depressive disorder	paroxetine, etizolam, zolpidem, valsartan, verapamil	yes	yes	yes	yes	no

		Biological Psychiatry, 31(1)										
Thumtecho et al	2021	Toxicology Reports, 8	Thailand	20	female	major depression	vortioxetine, escitalopram, bupropion, lamotrigine, lurasidone	yes	yes	yes	yes	no
Tiamfook et al	2005	The Journal of Emergency Medicine, 28(2)	USA	51	male	bipolar disorder	sertraline, tramadol	yes	yes	yes	yes	no
Timmerman & Hoek-Mensink	2009	Eur. Neuropsychopharmacol, 19	Netherlands	89	female	bipolar disorder	citalopram, tryptophan, valproate, tramadol, temazepam	no	no	yes	no	no
Tissot	2003	Anesthesiology, 98(6)	USA	41	male	NA	fluoxetine, pethidine, rosiglitazone, rosiglitazone, rosiglitazone, fenofibrate, midazolam	no	yes	yes	no	no
Toffel et al	2022	Journal of the Academy of Consultation-Liaison Psychiatry, 63	USA	23	male	polysubstance abuse	trazodone, bupropion, dextromethorphan	yes	yes	yes	NA	NA
Tomaselli & Modestin	2004	Pharmacopsychiatry, 37(5)	Switzerland	64	female	depression	sertraline, oxazepam	no	yes	yes	no	no
Top et al	2014	The Netherlands Journal of Medicine, 72(3)	Netherlands	70	female	mood disorder	venlafaxine, methylene blue	yes	yes	yes	yes	yes
Torres et al	2021	J. Gen. Intern. Med, 36	USA	46	female	depression	trazodone, paracetamol, gabapentin, clonazepam, pentoxifylline, zolpidem, tramadol, oxycodone	yes	yes	no	no	no
Troya et al	2019	International Journal of STD & AIDS, 30(10)	Spain	26	male	psychotic episodes	mephedrone, methamphetamine, linezolid, rilpivirine, emtricitabine, tenofovir	yes	yes	yes	yes	yes
Trungu et al	2019	Minerva Anestesiol, 85(5)	Belgium	65	female	NA	amitriptyline, mirtazapine, bupropion, amisulpride, aripiprazole, tramadol, nefopam	no	yes	yes	yes	yes
Tsamatsoulis et al	2018	Interactive CardioVascular and Thoracic Surgery, 26(5)	Greece	65	male	depression	citalopram, omeprazole	no	yes	yes	yes	no
Tschoe et al	2020	Neurology, 94(15)	Netherlands	63	male	depression	duloxetine, ropinirole	yes	yes	yes	no	no
Tseng et al	2005	The Kaohsiung Journal of Medical Sciences, 21(7)	China	32	male	major depressive disorder	citalopram, flupentixol	no	yes	no	no	no
TUREDI et al	2007	NeuroToxicology, 28(6)	Turkey	40	male	depression	citalopram	yes	yes	yes	no	no
Ubogu & Katirji	2003	Clinical Neuropharmacology, 26(2)	USA	85	female	major depressive disorder	mirtazapine, salmeterol, losartan, acetylsalicylic acid, fluvastatin, fluticasone	no	yes	yes	no	no
Varatharaj & Moran	2014	BMJ Case Reports, 2014	UK	54	female	suicide attempt	venlafaxine	no	no	yes	no	no
Vari & Beckson	2007	Journal of Clinical Psychopharmacology, 27(2)	USA	35	female	depression	escitalopram, mirtazapine buprenorphine, aripiprazole	yes	yes	yes	no	no
Varma et al	2022	Cureus, 14(8)	USA	54	female	bipolar disorder	trazodone, sertraline, memantine, donepezil, lorazepam	yes	yes	yes	no	no
Veilleux & Leung	2016	Crit. Care Med, 44(12)	USA	30	female	NA	venlafaxine, methylene blue	no	yes	yes	yes	no
Velez et al	2004	Annals of Pharmacotherapy, 38(2)	USA	57	male	depression	paroxetine, ethanol	yes	yes	yes	yes	no
Vena et al	2006	The Journal of Emergency Medicine, 30(3)	USA	21	male	depression	fluoxetine, olanzapine	no	no	yes	no	no

Verre et al	2008	Minerva Anestesiologica, 74(1–2)	Italy	51	male	depression	clomipramine, olanzapine, candesartan, insulin	yes	yes	yes	yes	no
Vizcaychipi et al	2007	Br. J. Anaesth, 99(6)	UK	49	male	NA	tramadol, morphine	no	no	yes	no	no
Vollman et al	2014	Clin. Toxicol, 52(7)	USA	25	male	NA	DMT	yes	yes	yes	no	no
Wagle et al	2019	Cureus, 11(5)	USA	22	NA	depression	fluoxetine, bupropion, ethanol	yes	yes	yes	yes	no
Walczyk et al	2016	Hospital Pharmacy, 51(4)	USA	48	male	major depressive disorder	duloxetine, amitriptyline tapentadol, oxycodone	yes	yes	yes	yes	no
Walia et al	2018	Journal of the American Geriatrics Society, 66(S2)	USA	69	female	anxiety disorder	escitalopram, trazodone, mirtazapine, olanzapine, lorazepam, gabapentin	no	no	yes	no	no
Walter et al	2012	Case Reports in Oncological Medicine, 2012	Australia	77	female	NA	citalopram, oxycodone, docusate, temazepam, esomeprazole	no	yes	no	no	no
Wang et al	2024	BMC Geriatrics, 24(1)	China	75	female	depression	escitalopram, levodopa, quetiapine, benserazide	yes	yes	yes	no	no
Waring et al	2006	Human & Experimental Toxicology, 25(12)	UK	35	male	NA	olanzapine, paracetamol	no	yes	yes	no	no
Warner et al, case 1	2017	Canadian Journal of Anesthesia, 64(9)	USA	72	male	depression	fluoxetine, fentanyl, acyclovir, digoxin, lisinopril	yes	yes	no	no	no
Warner et al, case 2	2017	Canadian Journal of Anesthesia, 64(9)	USA	19	male	major depression	fluoxetine, trazodone, fentanyl	yes	yes	no	no	no
Warrick et al	2012	Journal of Medical Toxicology, 8(1)	USA	24	female	NA	methylone, butylone	yes	yes	yes	yes	yes
Watson	2018	J. Intensive Care Soc, 19(2)	USA	61	male	NA	citalopram, meropenem, linezolid, oxycodone	yes	yes	yes	yes	no
Watts & Yousaf	2000	Psychiatr. Bull, 24(11)	UK	53	male	recurrent depressive episodes	paroxetine, lithium	yes	yes	no	no	no
Weibrecht & Boyer	2010	Clin. Toxicol, 48(3)	USA	27	female	NA	fluoxetine, MDMA	yes	yes	yes	yes	no
Weiler et al	2013	BMJ Case Reports, 2013	Switzerland	45	male	depression	venlafaxine, codeine, sumatriptan	yes	yes	yes	no	no
Weiner et al	1998	Pharmacotherapy, 18(2)	USA	44	female	anorexia nervosa	phenelzine, venlafaxine, alprazolam	yes	yes	yes	yes	no
Weis et al	2014	J. Med. Toxicol, 10(1)	USA	38	female	depression	paroxetine, methylene blue, dapsone	yes	yes	yes	no	no
Weiss	1995	The Journal of the American Board of Family Practice, 8(5)	USA	50	male	Parkinson's disease	sertraline, carbidopa, selegiline, levodopa	yes	yes	yes	no	no
Whipp & Waterfield	2004	Palliative Medicine, 18(1)	UK	85	male	depression	sertraline, morphine, metoclopramide, celecoxib, goserelin, dantron, docusate, metoprolol, lisinopril, furosemide, amiloride, megestrol, isosorbide mononitrate, fluconazole, goserelin	yes	yes	yes	no	no
Wiegand, case 1	2013	Clin. Toxicol, 51(4)	USA	27	male	methylphenidate abuse	bupropion	yes	yes	yes	no	no
Wiegand, case 2	2013	Clin. Toxicol, 51(4)	USA	51	male	polysubstance abuse	bupropion	yes	yes	yes	no	no
Wigen & Goetz	2002	Clinical Infectious Diseases, 34(12)	USA	56	female	depression	paroxetine, linezolid, morphine, felodipine, terazosin, lisinopril, insulin, methocarbamol, ibuprofen	no	yes	yes	no	no

Wilson et al	2012	Am. J. Otolaryngol. Head Neck Med. Surg, 33(3)	USA	42	female	depressive disorder	citalopram, ondansetron, amphetamine, quetiapine	yes	yes	yes	no	no
Winograd et al	2018	J. Med. Toxicol, 14(1)	USA	23	male	NA	tianeptine	yes	yes	yes	yes	no
Wolvetang et al	2016	Journal of Cardiothoracic and Vascular Anesthesia, 30(4)	Netherlands	64	female	depression	paroxetine, methylene blue, clonazepam, quetiapine	no	yes	yes	no	no
Wong et al	2002	JRSM, 95(6)	Taiwan	53	male	depression	sertraline, cyclosporine	yes	yes	yes	yes	no
Wood et al	2007	Clinical Toxicology, 45(7)	UK	25	female	depression	fluvoxamine	no	no	yes	yes	yes
Wu & Deng	2009	Journal of the Chinese Medical Association, 72(8)	China	53	female	depression	paroxetine, moclobemide midodrine, biperiden, ginkgo-flavone	yes	yes	yes	no	no
Wu & Deng	2011	Chang Gung Medical Journal, 34(6)	China	36	female	major depression	moclobemide, fluoxetine propranolol, estazolam	yes	yes	yes	yes	yes
Wu & Hill	2024	Canadian Journal of Hospital Pharmacy, 77(1)	Canada	47	male	treatment-refractory schizophrenia	clozapine, zuclopenthixol decanoate	yes	yes	yes	no	no
Wu et al	2015	Acta Neurol Taiwan, 24(4)	China	34	male	schizophrenic disorder	mirtazapine, olanzapine	yes	yes	yes	yes	no
Wu et al	2023	Frontiers in Psychiatry, 14	China	74	female	schizophrenia	escitalopram, clozapine, clopidogrel	yes	yes	yes	no	no
Yacoub et al	2010	Neurology, 74(8)	USA	76	male	depression	paroxetine, milnacipran	no	yes	yes	no	no
Yalamarti & Lee	2019	Chest, 156(4)	Netherlands	68	female	NA	fentanyl	no	yes	yes	yes	no
Yamada et al	2021	Clinical Parkinsonism & Related Disorders, 5	Japan	41	female	depression	paroxetine, tramadol	yes	no	no	no	no
Yates et al	2011	Therapeutic Advances in Psychopharmacology, 1(4)	UK	42	female	major depression	phenelzine, venlafaxine, lithium	no	yes	yes	no	no
Yazdi et al	2023	Cureus, 15(5)	USA	41	female	bipolar disorder	mirtazapine, escitalopram, sumatriptan	no	yes	yes	yes	no
Yee & Wijdicks	2010	Neurocritical Care, 12(2)	USA	74	male	Alzheimer's dementia	citalopram, prochlorperazine, risperidone, memantine, donepezil	yes	yes	yes	no	no
Yeoh	2018	Anesthesiol Case Rep, 1(1)	USA	35	female	depression	duloxetine, methylene blue, fentanyl	no	no	yes	yes	no
Yoo et al	2018	J. Am. Geriatr. Soc, 66	USA	67	male	depression	citalopram, bupropion tramadol, fentanyl, clonazepam, ondansetron	yes	yes	yes	yes	no
Yoshida et al	2015	Forensic Toxicol, 33(2)	Japan	20	male	NA	NBOME compounds	yes	yes	yes	yes	yes
Zand et al	2010	Mayo Clin. Proc, 85(10)	USA	74	female	Alzheimer's disease	citalopram, risperidone, memantine, donepezil, sulfamethoxazole, trimethoprim	yes	yes	yes	no	no
Zayac et al	2016	Crit. Care Med, 44(12)	USA	74	female	major depressive disorder	paroxetine, trazodone	yes	yes	yes	yes	no
Zerjav-Lacombe & Dewan	2001	Annals of Pharmacotherapy, 35(2)	Canada	44	male	undifferentiated schizophrenia	clomipramine, clozapine, clonazepam	no	yes	yes	no	no
Zhan et al	2021	Psychiatr. Danub, 33(2)	China	18	male	major depression	venlafaxine, valproate	yes	yes	yes	no	no
Zhang et al	2016	BMJ Case Rep, 2016	Canada	39	male	NA	bupropion, escitalopram olanzapine, primidone	yes	yes	yes	yes	no
Zick et al	2019	Curr. Psychiatry, 21(3)	USA	55	male	major depressive disorder	sertraline, trazodone lisinopril, sumatriptan	yes	yes	yes	yes	no

Zimmerschied & Harry	1998	Journal of Forensic Sciences, 43(1)	USA	46	male	major depressive disorder	paroxetine, desipramine haloperidol, hydroxyzine, benztropine	yes	yes	yes	no	no

NA: not available

Supplementary Table S6. Summary of PHARMA checklists of included cases

Category	N Fulfilled (%)
Demographics reported	758 (99.2)
Current health status reported	764 (100)
Medical history reported	694 (90.8)
Physical examination reported	563 (73.7)
Patient disposition reported	753 (98.6)
Drug identified	764 (100)
Drug dose reported	598 (78.3)
Drug administration reported	598 (78.3)
Drug-reaction interface reported	623 (81.5)
Concomitant therapies reported	764 (100)
Adverse event description provided	764 (100)
Discussion provided	764 (100)

Supplementary material S7: Search strategy

EMBASE:
('serotonin syndrome'/exp OR 'serotonin syndrome' OR 'serotonin toxicity'/exp OR 'serotonin toxicity') AND ('tricyclic antidepressant agent'/exp OR 'tricyclic antidepressant agent' OR 'serotonin uptake inhibitor'/exp OR 'serotonin uptake inhibitor' OR 'serotonin noradrenalin reuptake inhibitor'/exp OR 'serotonin noradrenalin reuptake inhibitor' OR 'desipramine' OR 'imipramine' OR 'clomipramine' OR 'opipramol' OR 'trimipramine' OR 'nortrimipramine' OR 'nortriptyline' OR lofepramine OR 'dibenzepin' OR 'amitriptyline' OR 'protriptyline' OR 'doxepin' OR 'iprindole' OR 'melitracen' OR 'butriptyline' OR 'dosulepin' OR 'amoxapine' OR dimetracrine OR 'amineptine' OR 'maprotiline' OR 'quinupramine' OR 'zimeldine' OR 'fluoxetine' OR 'citalopram' OR 'escitalopram' OR 'paroxetine' OR 'sertraline' OR 'alaproclate' OR 'fluvoxamine' OR 'etoperidone' OR 'isocarboxazid' OR 'nialamide' OR 'phenelzine' OR 'tranylcypromine' OR 'iproniazid' OR 'iproclozide' OR 'moclobemide' OR 'toloxatone' OR oxitriptan OR 'tryptophan' OR 'mianserin' OR 'nomifensine' OR 'trazodone' OR 'nefazodone' OR 'minaprine' OR 'bifemelane' OR 'viloxazine' OR 'oxaflozane' OR 'mirtazapine' OR bupropion OR 'medifoxamine' OR 'tianeptine' OR 'pivagabine' OR 'venlafaxine' OR 'milnacipran' OR 'reboxetine' OR 'gepirone' OR 'duloxetine' OR 'agomelatine' OR 'desvenlafaxine' OR 'vilazodone' OR 'hypericum' OR 'vortioxetine' OR 'esketamine') AND ('case report'/it OR 'case series'/it)

MEDLINE:

("Serotonin Syndrome"[MeSH] OR "serotonin syndrome"[tiab] OR "serotonin toxicity"[tiab]) AND ("Antidepressive Agents, Tricyclic"[MeSH] OR "Serotonin Uptake Inhibitors"[MeSH] OR "Serotonin and Noradrenaline Reuptake Inhibitors"[MeSH] OR "desipramine"[tiab] OR "Desipramine"[MeSH] OR "imipramine"[tiab] OR "Imipramine"[MeSH] OR "clomipramine"[tiab] OR "Clomipramine"[MeSH] OR "opipramol"[tiab] OR "trimipramine"[tiab] OR "Trimipramine"[MeSH] OR "nortrimipramine"[tiab] OR "nortriptyline"[tiab] OR "Nortriptyline"[MeSH] OR "lofepramine"[tiab] OR "dibenzepin"[tiab] OR "amitriptyline"[tiab] OR "Amitriptyline"[MeSH] OR "protriptyline"[tiab] OR "Protriptyline"[MeSH] OR "doxepin"[tiab] OR "Doxepin"[MeSH] OR "iprindole"[tiab] OR "melitracen"[tiab] OR "butriptyline"[tiab] OR "dosulepin"[tiab] OR "amoxapine"[tiab] OR "Amoxapine"[MeSH] OR "dimetracrine"[tiab] OR "amineptine"[tiab] OR "maprotiline"[tiab] OR "Maprotiline"[MeSH] OR "quinupramine"[tiab] OR "zimeldine"[tiab] OR "fluoxetine"[tiab] OR "Fluoxetine"[MeSH] OR "citalopram"[tiab] OR "Citalopram"[MeSH] OR "escitalopram"[tiab] OR "Escitalopram"[MeSH] OR "paroxetine"[tiab] OR "Paroxetine"[MeSH] OR "sertraline"[tiab] OR "Sertraline"[MeSH] OR "alaproclate"[tiab] OR "fluvoxamine"[tiab] OR "Fluvoxamine"[MeSH] OR "etoperidone"[tiab] OR "isocarboxazid"[tiab] OR "Isocarboxazid"[MeSH] OR "nialamide"[tiab] OR "phenelzine"[tiab] OR "Phenelzine"[MeSH] OR "tranylcypromine"[tiab] OR "Tranylcypromine"[MeSH] OR "iproniazid"[tiab] OR "iproclozide"[tiab] OR "moclobemide"[tiab] OR "Moclobemide"[MeSH] OR "toloxatone"[tiab] OR "oxitriptan"[tiab] OR "tryptophan"[tiab] OR "Tryptophan"[MeSH] OR "mianserin"[tiab] OR "Mianserin"[MeSH] OR "nomifensine"[tiab] OR "Nomifensine"[MeSH] OR "trazodone"[tiab] OR "Trazodone"[MeSH] OR "nefazodone"[tiab] OR "Nefazodone"[MeSH] OR "minaprine"[tiab] OR "bifemelane"[tiab] OR "viloxazine"[tiab] OR "Viloxazine"[MeSH] OR "oxaflozane"[tiab] OR "mirtazapine"[tiab] OR "Mirtazapine"[MeSH] OR "bupropion"[tiab] OR "Bupropion"[MeSH] OR "medifoxamine"[tiab] OR "tianeptine"[tiab] OR

"pivagabine"[tiab] OR "venlafaxine"[tiab] OR "Venlafaxine Hydrochloride"[MeSH] OR "milnacipran"[tiab] OR "reboxetine"[tiab] OR "gepirone"[tiab] OR "duloxetine"[tiab] OR "Duloxetine Hydrochloride"[MeSH] OR "agomelatine"[tiab] OR "desvenlafaxine"[tiab] OR "Desvenlafaxine Succinate"[MeSH] OR "vilazodone"[tiab] OR "Vilazodone Hydrochloride"[MeSH] OR "hypericum"[tiab] OR "Hypericum"[MeSH] OR "vortioxetine"[tiab] OR "Vortioxetine"[MeSH] OR "esketamine"[tiab]) AND ("Case Reports"[pt] OR "case series"[tiab])
